# Synthesis and In Vitro Anticancer Activity of Novel Dehydroabietic Acid-Based Acylhydrazones

**DOI:** 10.3390/molecules22071087

**Published:** 2017-06-29

**Authors:** Fang-Yao Li, Xiu Wang, Wen-Gui Duan, Gui-Shan Lin

**Affiliations:** 1 School of Chemistry and Chemical Engineering, Guangxi University, Nanning 530004, Guangxi, China; lifangyao@glmc.edu.cn (F.-Y.L.); gslin@gxu.edu.cn (G.-S.L.); 2 College of Pharmacy, Guilin Medical University, Guilin 541004, Guangxi, China; wangxiumt2015@163.com

**Keywords:** dehydroabietic acid, acylhydrazone, anticancer activity, synthesis

## Abstract

In order to develop novel chemotherapeutic agents with potent anticancer activities, a series of dehydroabietic acid (DHA) derivatives bearing an acylhydrazone moiety were designed and synthesized by the condensation between dehydroabietic acylhydrazide (**3**) and a variety of substituted arylaldehydes. The inhibitory activities of these compounds against CNE-2 (nasopharynx), HepG2 (liver), HeLa (epithelial cervical), and BEL-7402 (liver) human carcinoma cell lines were evaluated by 3-(4,5-dimethylthiazol-2-yl)-2,5-diphenyl tetrazolium bromide (MTT) assay in vitro. The screening results revealed that many of the compounds showed moderate to high levels of anticancer activities against the tested cancer cell lines and some displayed similar potent inhibitory activities to the commercial anticancer drug cisplatin, while they exhibited lower cytotoxicity against normal human liver cell (HL-7702). Particularly, compound **4w**, *N*’-(3,5-difluorobenzylidene)-2-(dehydroabietyloxy)acetohydrazide, with an IC_50_ (50% inhibitory concentration) value of 2.21 μM against HeLa cell, was about 17-fold more active than that of the parent compound, and showed remarkable cytotoxicity with an IC_50_ value of 14.46 μM against BEL-7402 cell. These results provide an encouraging framework that could lead to the development of potent novel anticancer agents.

## 1. Introduction

Cancer is among the leading causes of death on a global scale and currently the mortality rate has shown an increase in the recent past [1,2,3]. Chemotherapy represents one of the most important therapeutic strategies against various kinds of cancer; however, the available anticancer drugs usually cause toxicity to non-malignant tissues and lead to the development of resistance. Therefore, there is an urgent need for novel molecules with higher selectivity and more potent anticancer activities. Natural products have played a dominant role in drug discovery, especially since around 60% of all anticancer approved drugs are derived from natural resources [4]. Modification of the biologically active natural products has led to the development of potentially important bioactive molecules, leads, and drugs [5]. Encouraged by these results, our interest in investigating natural products for their potential therapeutic effects has recently encouraged us to examine the influences of dehydroabietic acid (DHA) derivatives for their anticancer activity.

DHA is a naturally occurring tricyclic diterpenic resin acid, which can easily be obtained from *Pinus* rosin or commercial disproportionated rosin [6]. Recently, DHA and its derivatives were claimed to possess a wide range of biological and pharmacological activities, such as antiprotozoal [7], antiulcer [8], anti-inflammatory [9], immunomodulatory, antiviral [10], antimicrobial [11,12], antifungal [13], anxiolytic [14], anti-aging [15], gastroprotective [16], and BK channel-opening [17] activities. In addition, in recent years a number of DHA derivatives have been reported to have anticancer activity in many human cancer cells such as cervical carcinoma cells, hepatocarcinoma cells, lung cancer cells, prostate cancer cells, ovarian cancer cells, and breast cancer cells [18,19,20]. These results suggest that dehydroabietic acid is a promising starting material for the synthesis of new anticancer agents.

On the other hand, acylhydrazone derivatives have been claimed to possess various bioactivities. 8-Hydroxyquinoline containing an acylhydrazone subunit has been reported as a definite potential therapeutic agent against Parkinson’s disease [21]. Some acylhydrazone derivatives bearing 1,3,4-oxadiazole showed excellent antiproliferative activity [22]. *N′*-(4-Arylidene)-1*H*-benzo[d]imidazole-2-carbohydrazides demonstrated in vitro inhibitory activity against human tumor cell lines in the low micromolar range [23]. Salicylaldehyde 1-arylmethyl-3-1*H*-pyrazole-5-carbohydrazide hydrazine derivatives behaved as iron chelators especially in A549 lung cancer cells and this was correlated to their cytotoxic activity [24]. In particular, (*E*)-2-hydroxybenzaldehyde-5-(2,4-difluorophenyl)-2-furoyl hydrazone (50% inhibitory concentration (IC_50_) = 16.4 μM) exhibited better anticancer activity than doxorubicin (IC_50_ = 53.3 μM) against human promyelocytic leukemic cells (HL-60) [25].

Furthermore, a series of dehydroabietic acid and α-pinene derivatives were synthesized and some of them exhibited good antifungal [26,27,28], herbicidal [29,30], and insecticidal [31] activities. Prompted by the aforementioned findings and in continuation of our ongoing research in the field of design, synthesis, and biological evaluation of DHA derivatives, we present in this paper the synthesis and evaluation of a new series of acylhydrazone derivatives from DHA as potential anticancer agents against CNE-2 (Nasopharynx), HepG2 (liver), HeLa (epithelial cervical), and BEL-7402 (liver) human cancer cell lines and HL-7702 normal human liver cell line.

## 2. Results and Discussion

### 2.1. Synthesis 

**Scheme 1:**
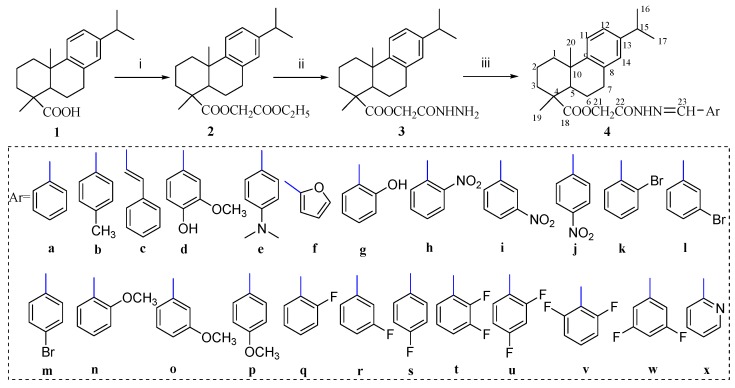
Synthesis of target compounds. Reagents and conditions: (i) ClCH_2_COOC_2_H_5_, K_2_CO_3_, Dimethylformamide (DMF), 40 °C, 4 h, yield 85%; (ii) NH_2_NH_2_·H_2_O, EtOH, 80 °C, 3 h, yield 77%; (iii) Ar-CHO, EtOH, HOAc, 80 °C, 6 h, yield 54–90%.

The synthesis of new DHA derivatives **4a**–**x** was carried out according to the protocol shown in Scheme 1. All these derivatives are new compounds not previously reported. Esterification of dehydroabietic acid (**1**) with ethyl chloroacetate in the presence of potassium carbonate in dimethylformamide (DMF) gave dehydroabietic ethyl acetate (**2**). High yields of acylhydrazide (**3**) were achieved upon aminolysis in an ethanolic solution of **2** and hydrazine hydrate by stirring at 80 °C for 3 h. Acylhydrazones **4a**–**x** were obtained in good to excellent yield by coupling the acylhydrazide **3** with the appropriate arylaldehydes in ethanol.

### 2.2. Evaluation of Anticancer Activity

Compounds **4a**–**x** were evaluated for their cytotoxic activity in vitro against the human cancer cell lines, CNE-2, HepG2, HeLa, and BEL-7402 by using a 3-(4,5-dimethylthiazol-2-yl)-2,5-diphenyl tetrazolium bromide (MTT) colorimetric assay. Cisplatin was included in the experiments as a positive control, and the IC_50_ of DHA is also presented to compare the anticancer activities. The calculated IC_50_ values were reported differently according to the different cancer cells. The results are summarized in Table 1.

As shown in Table 1, most of the target compounds showed moderate to high anticancer activity, revealing that introduction of the acylhydrazone on the skeleton of DHA could markedly improve the anticancer activity. In CNE-2 cells, most of the compounds (such as **4a**–**g**, **4i**, **4k**–**l,** and **4n**–**x**) displayed better cytotoxicity than DHA (IC_50_ = 88.64 μM) with IC_50_ in the range of 11.45–86.46 μM. Among these compounds, compound **4l** showed the best cytotoxicity, with IC_50_ of 11.45 μM. It was noted that changing Ar = phenyl compound **4a** to 2-pyridyl (**4x**) markedly increased this activity. The substituents in phenyl of compound **4** have important influence on the cytotoxic inhibition and the introduction of electron donor substituents may result in the decrease of cytotoxicity.

In HepG2 cells, most of the compounds (except **4q** and **4s**) exhibited better inhibition than DHA (IC_50_ = 80.36 μM) with IC_50_ in the range of 7.62–57.84 μM. From the data, compounds **4e**, **4k**, and **4x** showed the best inhibition with IC_50_ of 7.62, 8.47 and 8.07, respectively. We found that introduction of methyl, dimethylamino, hydroxyl, and difluoro groups into benzene group of acylhydrazone moiety showed a positive influence on antitumor activities in the HepG2 assay, while nitro, methoxyl, and fluoro groups exhibited a negative effect. 

In HeLa cells, all compounds showed better cytotoxicity than DHA (IC_50_ = 37.40 μM), with IC_50_ in the range of 2.21–34.92 μM. In particular, compound **4w** showed the highest cytotoxicity with the lowest IC_50_ value of 2.21 μM on HeLa cells, which possessed almost the same potency as that of positive drug cisplatin (IC_50_ = 1.94 μM) and was 16.92 times more active than DHA. The antitumor activities of tested were found to be in the order of cisplatin > **4w** > **4x** > **4k** > **4u** > **4b** > **4c** > **4t** > **4d** > **4r** > **4m** > **4j** > **4i** > **4v** > **4h** > **4a** > **4l** > **4g** > **4q** > **4s** > **4o** > **4f** > **4p** > **4e** > DHA > **4n**. Evidently, methyl at *para*, bromo at *ortho*, as well as bifluoro at *ortho* and *para* or at *meta* position in benzene group of acylhydrazone moiety may result in the enhancement of antitumor activity, while the presence of dimethylamino, hydroxyl, and methoxyl in benzene lead to the decrease of antitumor activity. In addition, displacement of benzene with pyridine appeared to have a positive influence on the antitumor activity in this assay.

In BEL-7402 cells, IC_50_ values of all compounds ranged from 14.46 to 48.16 μM, while the IC_50_ value of the parent compound DHA is 46.70 μM. Compounds **4o** and **4w** displayed similar potent inhibitory activities (IC_50_ = 14.77 and 14.46 μM) compared with positive control cisplatin (IC_50_ = 12.68 μM). Based on the results, it could be summarized that methyl and dimethylamino at *para*, and methoxyl and bifluoro at *meta* positions in the benzene group of the acylhydrazone moiety improve the antitumor activity against the BEL-7402 cell line, while the presence of a nitro group in benzene showed a negative effect.

## 3. Experimental Section

### 3.1. General Information

All the chemicals and reagents were commercially available and used without further purification. Routine thin-layer chromatography (TLC) was performed on silica gel plates (silica gel GF254 from Qingdao Haiyang Chemical Co., Ltd., Qingdao, China), preparative flash column chromatography was performed on the 200–300 mesh silica gel (Qingdao Haiyang Chemical Co., Ltd., Qingdao, China). Melting points were recorded on an X-4 microscope melting point apparatus (Beijing Tech Instrument Co., Ltd., Beijing, China) without calibration. ^1^H- and ^13^C-NMR spectra were recorded on a Bruker Avance III HD 600 MHz spectrometer (Bruker Co., Ltd., Zurich, Switzerland) at room temperature with tetramethylsilane (TMS) as an internal standard and CDCl_3_ as solvents. Chemical shifts are expressed in (ppm) and coupling constants (*J*) in Hz. Mass spectra were recorded with a liquid chromatograph mass spectrometer (Shimazu Co., Ltd., Kyoto, Japan). Infrared spectra (IR) were performed on Prestige-21 FTIR spectrometer (Shimadzu Co., Ltd., Nakagyo-ku, Japan). NMR, IR, and mass spectra of compounds **2**, **3**, and **4a**–**x** can be found at Supplementary Materials.

### 3.2. Synthesis of 2-(Dehydroabietyloxy) Aceticether *(**2**)*

To a solution of dehydroabietic acid (3.00 g, 10.0 mmol) and ethyl chloroacetate (2 mL, 14.1 mmol) in DMF (20 mL), anhydrous potassium carbonate 2.8 g (4.0 mmol) was added, and the reaction mixture was stirred at 40 °C for 4 h. The progress of the reaction was monitored by TLC. Upon completion, the reaction mixture was poured into iced water and extracted with ethyl acetate (3 × 40 mL). The separated organic phase was washed with brine solution, dried over anhydrous sodium sulfate, and then concentrated under reduced pressure. The crude products were purified by silica gel column chromatography (PET/EtOAc = 10:1, *v/v*) to obtain colorless oily liquid compound **2**. Yield 85%. IR (KBr, cm^−1^): 2954, 2872, 1762 (C=O), 1735 (C=O), 1498, 1463, 1382, 1209, 1168, 1120, 1029, 823. ^1^H-NMR (CDCl_3_, 600 MHz): δ 7.18 (d, *J* = 8.4 Hz, 1H, H-11), 7.00 (d, *J* = 8.4 Hz, 1H, H-12), 6.91 (s, 1H, H-14), 4.62 (d, *J* = 14.0 Hz, 1H, H-21), 4.55 (d, *J* = 14.0 Hz, 1H, H-21), 4.22 (q, *J* =7.0 Hz, 2H, OCH_2_), 2.81–3.00 (m, 3H, H-9 and H-17), 2.32 (d, *J* = 12.0 Hz, 2H, H-6 and H_e_-4), 1.52–1.87 (m, 7H, H-2, H-3, Ha-4 and H-10), 1.34 (s, 3H, CH_3_, H-19), 1.29 (t, *J* = 6.0 Hz 3H, CH_2_CH_3_), 1.24 (s, 6H, H-16 and H-17), 1.23 (s, 3H, H-20). ^13^C-NMR (151 MHz, CDCl_3_) δ 179.44 (C=O), 169.40 (C=O), 148.27, 147.12, 136.26, 128.35, 125.54, 125.28, 62.71, 62.21, 49.01, 46.04, 39.35, 38.33, 38.04, 34.89, 31.42, 26.61, 25.43, 23.00, 19.97, 17.93, 15.55.

### 3.3. Synthesis of 2-(Dehydroabietyloxy) Acetohydrazide *(**3**)*

A mixture of 2-(dehydroabietyloxy) aceticether (1.93 g, 5.0 mmol), and 80% hydrazine hydrate (2 mL, 32.9 mmol) in EtOH (20 mL) was refluxed for 3 h. After cooling, the formed precipitate was filtered off, washed with water and then recrystallized from ethanol to give white solid. Yield 77%. m.p. 115.2–116.2 °C. IR (KBr, cm^−1^): 3464, 3190 (N–H), 3122, 2956, 1732 (C=O), 1680 (C=O), 1421, 1238, 1170, 1124, 823. ^1^H-NMR (600 MHz, CDCl_3_) δ 9.46 (s, 1H, CONH), 7.18 (d, *J* = 8.2 Hz, 1H, H-11), 7.00 (d, *J* = 9.3 Hz, 1H, H-12), 6.89 (s, 1H, H-14), 4.62–5.00 (m, 2H, H-21), 2.91–2.99 (m, 1H, H-15), 2.81–2.89 (m, 2H, H-7), 2.31–2.35 (m, 2H, H_e_-1, H-5), 2.00 (s, 2H, NH_2_), 1.50–1.98 (m, 7H, H_a_-1, H-2, H-3 and H-6), 1.33 (s, 3H, H-19), 1.24 (s, 6H, H-16 and H-17), 1.23 (s, 3H, H-20). ^13^C-NMR (151 MHz, CDCl_3_) δ 178.40 (C=O), 169.54 (C=O), 147.10, 145.78, 135.09, 127.09, 124.30, 123.96, 61.74, 47.73, 44.77, 38.10, 37.08, 33.62, 30.22, 25.53, 25.39, 24.17, 21.74, 18.76, 16.73. MS (ESI) *m/z* 356.84 ([M − H]^−^).

### 3.4. General Procedure for the Synthesis of Dehydroabietic Acid-Based Acylhydrazones ***4a**–**x***

A mixture of acylhydrazide **3** (2 mmol), the appropriate arylaldehyde (2.2 mmol) and glacial acetic acid (0.25 mL) in EtOH (20 mL) was refluxed for 6 h. After cooling, the formed precipitate was filtered off and purified by crystallization from anhydrous ethanol to afford the acylhydrazone derivatives.

*N’-(Benzylidene)-2-(dehydroabietyloxy)acetohydrazide* (**4a**): White solid; yield 87%, m.p. 155.1–156.7 °C. IR (KBr, cm^−1^): 3446, 3197 (N–H), 2951, 2864, 2366, 1732 (C=O), 1697 (C=O), 1614, 1492, 1411, 1296, 1232, 1174, 1118, 1022, 947, 819, 754, 690. ^1^H-NMR (600 MHz, CDCl_3_) δ 10.70 (s, 1H, CONH), 7.84 (s, 1H, N=CH), 7.64 (d, *J* = 9.2 Hz, 2H, H-2′ and H-6′), 7.40 (d, *J* = 6.2 Hz, 3H, H-3′, H-4′ and H-5′), 7.22 (d, *J* = 8.2 Hz, 1H, H-11), 7.03 (d, *J* = 8.2 Hz, 1H, H-12), 6.89 (s, 1H, H-14), 5.19 (q, *J* = 16.0 Hz, 2H, H-21), 3.05 (m, 1H, H-15), 2.82–2.91 (m, 2H, H-7), 2.42 (dd, *J* = 12.3, 1.7 Hz, 1H, H_e_-1), 2.36 (d, *J* = 12.9 Hz, 1H, H-5), 1.52–1.93 (m, 7H, H_a_-1, H-2, H-3 and H-6), 1.37 (s, 3H, H-19), 1.27 (s, 3H, H-20), 1.21 (d, *J* = 6.9 Hz, 6H, H-16 and H-17). ^13^C-NMR (151 MHz, CDCl_3_) δ 178.51 (C=O), 170.17 (C=O), 147.08, 145.85, 145.52, 135.02, 133.66, 130.53, 128.95, 127.37, 127.13, 124.36, 124.04, 61.54, 47.86, 44.88, 38.14, 37.12, 36.98, 33.62, 30.32, 25.46, 24.18, 21.82, 18.81, 16.81. MS (ESI) *m/z* 458.86 ([M − H]^−^).

*N’-(4-Methylbenzylidene)-2-(dehydroabietyloxy)acetohydrazide* (**4b**): White solid; yield 89%, m.p. 170.0–172.1 °C. IR (KBr, cm^−1^): 2951, 2929, 1737 (C=O), 1691 (C=O), 1610, 1498, 1417, 1311, 1236, 1172, 1124, 954, 891, 813. ^1^H-NMR (600 MHz, CDCl_3_) δ 10.62 (s, 1H, CONH), 7.80 (s, 1H, N=CH), 7.53 (d, *J* = 8.1 Hz, 2H, H-2′ and H-6′), 7.21 (d, *J* = 12.4 Hz, 3H, H-3′, H-5′ and H-11), 7.03 (d, *J* = 8.1 Hz, 1H, H-12), 6.88 (s, 1H, H-14), 5.17 (q, *J* = 16.0 Hz, 2H, H-21), 3.01 (d, *J* = 7.1 Hz, 1H, H-15), 2.79–2.92 (m, 2H, H-7), 2.42 (dd, *J* = 12.5, 2.0 Hz, 1H, H_e_-1), 2.40 (s, 3H, Ph-CH_3_), 2.35 (d, *J* = 13.2 Hz, 1H, H-5), 1.53–2.01 (m, 7H, H_a_-1, H-2, H-3 and H-6), 1.37 (s, 3H, H-19), 1.27 (s, 3H, H-20), 1.21 (d, *J* = 6.9 Hz, 6H, H-16 and H-17). ^13^C-NMR (151 MHz, CDCl_3_) δ 178.50 (C=O), 170.00 (C=O), 147.09, 145.82, 145.57, 140.83, 135.05, 130.97, 129.67, 127.33, 127.13, 124.34, 124.01, 61.56, 47.85, 44.88, 38.13, 37.12, 36.97, 33.61, 30.32, 25.46, 24.16, 21.81, 21.70, 18.81, 16.81. MS (ESI) *m/z* 472.88 ([M − H]^−^).

*N’-(3-Phenylallylidene)-2-(dehydroabietyloxy)acetohydrazide* (**4c**): light yellow solid; yield 89%, m.p. 185.7–187.3 °C. IR (KBr, cm^−1^): 2924, 1734 (C=O), 1687 (C=O), 1496, 1417, 1296, 1238, 1170, 1124, 972, 750, 690, 599. ^1^H-NMR (600 MHz, CDCl_3_) δ 10.45 (s, 1H, CONH), 7.63 (d, *J* = 7.1 Hz, 1H, 1H, N=CH), 7.46 (d, *J* = 7.4 Hz, 2H, H-2′ and H-6′), 7.38 (t, 2H, H-3′ and H-5’), 7.34 (d, *J* = 7.1 Hz, 1H, H-4′), 7.21 (d, *J* = 8.2 Hz, 1H, H-11), 7.02 (d, *J* = 8.9 Hz, 1H, H-12), 6.90 (s, 1H, H-14), 6.85 (s, 2H, HC=CH), 5.09 (q, *J* = 15.9 Hz, 2H, H-21), 2.99–3.05 (m, 1H, H-15), 2.81–2.93 (m, 2H, H-7), 2.41 (dd, *J* = 12.5, 2.0 Hz, 1H, H_e_-1), 2.36 (d, *J* = 12.9 Hz, 1H, H-5), 1.54–1.99 (m, 7H, H_a_-1, H-2, H-3 and H-6), 1.37 (s, 3H, H-19), 1.27 (s, 3H, H-20), 1.22 (d, *J* = 6.9, 2.0 Hz, 6H, H-16 and H-17). ^13^C-NMR (151 MHz, CDCl_3_) δ 178.47 (C=O), 169.84 (C=O), 147.47, 147.06, 145.86, 139.99, 135.86, 135.00, 129.28, 129.03, 127.25, 127.14, 124.68, 124.33, 124.03, 61.47, 47.85, 44.88, 38.13, 37.11, 36.95, 33.61, 30.30, 25.44, 24.15, 21.82, 18.81, 16.81. MS (ESI) *m/z* 484.91 ([M − H]^−^).

*N’-(3-Methoxy-4-hydroxylbenzylidene)-2-(dehydroabietyloxy)acetohydrazide* (**4d**): yellow solid; yield 85%, m.p. 117.6–119.0 °C. IR (KBr, cm^−1^): 3446, 3230 (N–H), 2954, 1730 (C=O), 1687 (C=O), 1598, 1516, 1462, 1425, 1282, 1238, 1170, 1122, 1029, 950, 862, 819, 756, 623. ^1^H-NMR (600 MHz, CDCl_3_) δ 10.35 (s, 1H, CONH), 7.72 (s, 1H, N=CH), 7.23 (s, 1H, H-6’), 7.19 (d, *J* = 8.2 Hz, 1H, H-2’), 7.01 (d, *J* = 8.0 Hz, 2H, H-5′ and H-11), 6.90 (d, *J* = 8.1 Hz, 1H, H-12), 6.87 (s, 1H, H-14), 5.15 (q, *J* = 15.9 Hz, 2H, H-21), 3.93 (s, 3H, OCH_3_), 2.98–3.02 (m, 1H, H-15), 2.76–2.93 (m, 2H, H-7), 2.39 (dd, *J* = 12.3, 1.5 Hz, 1H, H_e_-1), 2.33 (d, *J* = 12.7 Hz, 1H, H-5), 1.48–2.03 (m, 7H, H_a_-1, H-2, H-3, H-6), 1.37 (s, 3H, H-19), 1.26 (s, 3H, H-20), 1.23 (d, *J* = 6.9 Hz, 6H, H-16 and H-17). ^13^C-NMR (151 MHz, CDCl_3_) δ 178.58 (C=O), 169.65 (C=O), 148.24, 147.18, 147.04, 145.85, 145.66, 134.99, 127.10, 126.11, 124.31, 124.01, 122.89, 114.63, 107.78, 61.53, 56.11, 53.70, 47.86, 38.10, 37.09, 36.96, 33.60, 30.25, 25.42, 24.15, 21.81, 18.78, 16.79. MS (ESI) *m/z* 504.87 ([M − H]^−^).

*N’-(4-Dimethylaminobenzylidene)-2-(dehydroabietyloxy)acetohydrazide* (**4e**): brown red solid, yield 66%, m.p. 163.4–164.7 °C. IR (KBr, cm^−1^): 2953, 1735 (C=O), 1687 (C=O), 1606, 1531, 1417, 1359, 1298, 1234, 1174, 1116, 1047, 948, 815. ^1^H-NMR (600 MHz, CDCl_3_) δ 10.00 (s, 1H, CONH), 7.69 (s, 1H, N=CH), 7.50 (d, *J* = 8.5 Hz, 2H, H-2’ and H-6’), 7.20 (d, *J* = 8.2 Hz, 1H, H-11), 7.02 (d, *J* = 8.2 Hz, 1H, H-12), 6.89 (s, 1H, H-14), 6.70 (d, *J* = 8.5 Hz, 2H, H-3’ and H-5’), 5.14 (q, *J* =16.2 Hz, 2H, H-21), 3.03 (s, 6H, N(CH_3_)_2_), 2.94–3.00 (m, 1H, H-15), 2.80–2.90 (m, 2H, H-7), 2.40 (dd, *J* = 12.3, 1.4 Hz, 1H, H_e_-1), 2.31 (d, *J* = 12.7 Hz, 1H, H-5), 1.50–1.98 (m, 7H, H_a_-1, H-2, H-3, H-6), 1.37 (s, 3H, H-19), 1.26 (s, 3H, H-20), 1.22 (d, *J* = 6.9 Hz, 6H, H-16 and H-17). ^13^C-NMR (151 MHz, CDCl_3_) δ 178.48 (C=O), 169.22 (C=O), 147.12, 146.94, 145.91, 145.76, 134.86, 128.77, 127.14, 124.35, 124.31, 123.94, 112.10, 61.64, 47.83, 44.78, 40.49, 38.04, 37.11, 36.92, 33.62, 30.30, 25.42, 24.16, 21.78, 18.74, 16.68. MS (ESI) *m/z* 501.89 ([M − H]^−^).

*N’-((Furan-2-yl)methylene)-2-(dehydroabietyloxy)acetohydrazide* (**4f**): yellow solid; yield 58%, m.p. 125.7–126.4 °C. IR (KBr, cm^−1^): 2968, 2868, 1730 (C=O), 1683 (C=O), 1625, 1541, 1469, 1419, 1386, 1334, 1284, 1228, 1170, 1124, 1012, 974, 937, 883, 837, 750. ^1^H-NMR (600 MHz, CDCl_3_) δ 10.68 (s, 1H, CONH), 7.71 (s, 1H, N=CH), 7.50 (s, 1H, H-4’), 7.20 (d, *J* = 8.2 Hz, 1H, H-11), 7.01 (d, *J* = 8.1 Hz, 1H, H-12), 6.89 (s, 1H, H-14), 6.67 (d, *J* = 3.4 Hz, 1H, H-4’), 6.48 (s, 1H, H-4’), 5.12 (q, *J* = 16.1 Hz, 2H, H-21), 2.96–3.02 (m, 1H, H-15), 2.81–2.91 (m, 2H, H-7), 2.39 (dd, *J* = 12.3, 1.6 Hz, 1H, H_e_-1), 2.34 (d, *J* = 12.9 Hz, 1H, H-5), 1.52–1.98 (m, 7H, H_a_-1, H-2, H-3 and H-6), 1.37 (s, 3H, H-19), 1.27 (s, 3H, H-20), 1.23 (d, *J* = 6.9 Hz, 6H, H-16 and H-17). ^13^C-NMR (151 MHz, CDCl_3_) δ 178.44 (C=O), 170.16 (C=O), 149.15, 147.07, 145.83, 144.78, 135.24, 135.03, 127.11, 124.34, 124.01, 113.35, 112.09, 61.44, 47.82, 44.85, 38.11, 37.09, 36.89, 33.61, 30.27, 25.43, 24.16, 21.76, 18.78, 16.76. MS (ESI) *m/z* 448.86 ([M − H]^−^).

*N’-(2-Hydroxylbenzylidene)-2-(dehydroabietyloxy)acetohydrazide* (**4g**): yellow solid; yield 73%, m.p. 214.9–215.6 °C. IR (KBr, cm^−1^): 3251 (N–H), 2953, 2870, 1726 (C=O), 1678 (C=O), 1616, 1552, 1292, 1390, 1274, 1220, 1166, 1124, 974, 896, 759. ^1^H-NMR (600 MHz, CDCl_3_) δ 10.81 (s, 1H, CONH), 9.87 (s, 1H, OH), 8.12 (s, 1H, N=CH), 7.34 (m, 1H, H-6’), 7.20 (d, *J* = 8.2 Hz, 1H and H-4’), 7.15 (d, *J* = 6.8 Hz, 1H, H-5’), 6.96–7.04 (m, 2H, H-11 and H-14), 6.84–6.91 (m, 7.4 Hz, 2H, H-12 and H-3’), 5.05 (q, *J* = 15.5 Hz, 2H, H-21), 2.95–3.02 (m, 1H, H-15), 2.78–2.90 (m, 2H, H-7), 2.38 (dd, *J* = 12.4, 1.2 Hz, 1H, H_e_-1), 2.34 (d, *J* = 12.8 Hz, 1H, H-5),1.47–2.01 (m, 7H, H_a_-1, H-2, H-3 and H-6), 1.38 (s, 3H, H-19), 1.26 (s, 3H, H-20), 1.23 (d, *J* = 6.9 Hz, 6H, H-16 and H-17). ^13^C-NMR (151 MHz, CDCl_3_) δ 178.43 (C=O), 168.98 (C=O), 163.35, 157.91, 146.96, 145.91, 134.88, 132.39, 131.42, 127.12, 124.32, 124.07, 120.15, 119.53, 117.14, 60.92, 47.88, 44.83, 38.07, 37.08, 36.97, 33.61, 30.22, 25.40, 24.14, 21.85, 18.74, 16.77. MS (ESI) *m/z* 474.85 ([M − H]^−^).

*N’-(2-Nitrobenzylidene)-2-(dehydroabietyloxy)acetohydrazide* (**4h**): yellow solid; yield 62%; m.p. 94.9–96.7 °C. IR (KBr, cm^−1^): 3442, 3207 (N–H), 3101, 2954, 2927, 2868, 1728 (C=O), 1691 (C=O), 1597, 1527, 1463, 1417, 1346, 1301, 1238, 1170, 1126, 970, 935, 821, 783, 742. ^1^H-NMR (600 MHz, CDCl_3_) δ 10.48 (s, 1H, CONH), 8.38 (s, 1H, N=CH), 8.03 (d, *J* = 8.2 Hz, 2H, H-3’ and H-6’), 7.65 (t, *J* = 7.8 Hz, 1H, H-5’), 7.55 (t, *J* = 7.8 Hz, 1H, H-4’), 7.18 (d, *J* = 8.2 Hz, 1H, H-11), 7.00 (d, *J* = 8.1 Hz, 1H, H-12), 6.87 (s, 1H, H-14), 5.14 (q, *J* = 16.0 Hz, 2H, H-21), 2.95 (m, 1H, H-15), 2.85 (m, 2H, H-7), 2.35 (dd, *J* = 12.5, 1.7 Hz, 1H, H_e_-1), 2.32 (d, *J* = 13.1 Hz, 1H, H-5), 1.49–1.93 (m, 7H, H_a_-1, H-2, H-3 and H-6), 1.37 (s, 3H, H-19), 1.26 (s, 3H, H-20), 1.23 (d, *J* = 6.9 Hz, 6H, H-16 and H-17). ^13^C-NMR (151 MHz, CDCl_3_) δ 178.59 (C=O), 169.97 (C=O), 148.30, 147.11, 145.77, 140.59, 135.06, 133.55, 130.64, 128.71, 128.49, 127.08, 125.02, 124.31, 123.96, 61.38, 47.85, 44.80, 38.07, 37.08, 36.92, 33.60, 30.19, 25.42, 24.16, 21.78, 18.71, 16.71. MS (ESI) *m/z* 503.88 ([M − H]^−^).

*N’-(3-Nitrobenzylidene)-2-(dehydroabietyloxy)acetohydrazide* (**4i**): light yellow solid; yield 82%, m.p. 99.6–101.9 °C. IR (KBr, cm^−1^): 3190, 2926, 1734 (C=O), 1693 (C=O), 1579, 1533, 1463, 1348, 1238, 1172, 1126, 981, 825, 734, 678. ^1^H-NMR (600 MHz, CDCl_3_) δ 10.85 (s, 1H, CONH), 8.46 (s, 1H, N=CH), 8.24 (s, 1H, H-2′), 7.92 (s, 2H, H-5′ and H-6′), 7.55 (s, 1H, H-3′), 7.19 (d, *J* = 8.2 Hz, 1H, H-11), 7.02 (d, *J* = 9.3 Hz, 1H, H-14), 6.86 (s, 1H, H-12), 5.18 (q, *J* = 16.0 Hz, 2H, H-21), 2.96–3.02 (m, 1H, H-15), 2.77–2.91 (m, 2H, H-7), 2.31–2.42 (m, 2H, H_e_-1 and H-5), 1.49–2.01 (m, 7H, H_a_-1, H-2, H-3 and H-6), 1.39 (s, 3H, H-19), 1.26 (s, 3H, H-20), 1.21 (d, *J* = 6.9, Hz, 6H, H-16 and H-17). ^13^C-NMR (151 MHz, CDCl_3_) δ 178.60 (C=O), 170.44 (C=O), 148.76, 147.00, 145.90, 142.97, 135.39, 134.84, 132.84, 130.05, 127.07, 124.82, 124.34, 124.08, 121.84, 61.45, 47.86, 44.88, 38.10, 37.09, 36.93, 33.59, 30.23, 25.39, 24.15, 21.82, 18.74, 16.80. MS (ESI) *m/z* 503.87 ([M − H]^−^).

*N’-(4-Nitrobenzylidene)-2-(dehydroabietyloxy)acetohydrazide* (**4j**): yellow solid; yield 54%, m.p. 177.2–179.8 °C. IR (KBr, cm^−1^): 3099, 2947, 1739 (C=O), 1691 (C=O), 1591, 1523, 1463, 1413, 1342, 1234, 1170, 1111, 1014, 943, 833, 745. ^1^H-NMR (600 MHz, CDCl_3_) δ 10.63 (s, 1H, CONH), 8.24 (s, 2H, H-3’ and H-5’), 7.87 (s, 1H, N=CH), 7.75 (d, *J* = 8.6 Hz, 2H, H-2’ and H-6’), 7.20 (d, *J* = 8.2 Hz, 1H, H-11), 6.86 (s, 1H, H-12), 7.02 (d, *J* = 9.3 Hz, 1H, H-14), 5.17 (q, *J* = 16.0 Hz, 2H, H-21), 2.95–3.01 (m, 1H, H-15), 2.77–2.91 (m, 2H, H-7), 2.34–2.39 (m, 2H, H_e_-1 and H-5), 1.48–1.98 (m, 7H, H_a_-1, H-2, H-3 and H-6), 1.37 (s, 3H, H-19), 1.26 (s, 3H, H-20), 1.20 (d, *J* = 6.9, Hz, 6H, H-16 and H-17). ^13^C-NMR (151 MHz, CDCl_3_) δ 178.53 (C=O), 170.36 (C=O), 148.71, 146.96, 145.96, 134.92, 132.51, 132.28, 128.70, 127.09, 124.79, 124.34, 124.06, 61.39, 47.86, 44.91, 38.09, 37.13, 36.93, 33.58, 30.26, 25.40, 24.13, 21.80, 18.77, 16.79. MS (ESI) *m/z* 503.87 ([M − H]^−^).

*N’-(2-Bromobenzylidene)-2-(dehydroabietyloxy)acetohydrazide* (**4k**): White solid; yield 70%; m.p. 184.9–185.7 °C. IR (KBr, cm^−1^): 3246 (N–H), 2935, 1730 (C=O), 1699 (C=O), 1668, 1602, 1496, 1462, 1402, 1234, 1172, 1128, 945, 877, 754, 711, 638. ^1^H-NMR (600 MHz, CDCl_3_) δ 10.54 (s, 1H, CONH), 8.21 (s, 1H, N=CH), 7.92 (d, *J* = 7.8 Hz, 1H, H-6’), 7.58 (d, *J* = 7.9 Hz, 1H, H-3’), 7.34 (t, *J* = 7.6 Hz, 1H, H-5’), 7.23–7.27 (m, 1H, H-4’), 7.19 (d, *J* = 8.2 Hz, 1H, H-11), 7.01 (d, *J* = 9.3 Hz, 1H, H-14), 6.87 (s, 1H, H-12), 5.16 (q, *J* = 16.0 Hz, 2H, H-21), 2.94–3.00 (m, 1H, H-15), 2.80–2.89 (m, 2H, H-7), 2.32–2.39 (m, 2H, H_e_-1, H-5), 1.50–1.98 (m, 7H, H_a_-1, H-2, H-3 and H-6), 1.37 (s, 3H, H-19), 1.26 (s, 3H, H-20), 1.23 (d, *J* = 6.9 Hz, 6H, H-16 and H-17). ^13^C-NMR (151 MHz, CDCl_3_) δ 178.51 (C=O), 170.00 (C=O), 147.07, 145.80, 144.04, 135.06, 133.38, 132.63, 131.60, 127.78, 127.71, 127.11, 124.38, 124.32, 123.98, 61.48, 47.84, 44.81, 38.10, 37.10, 37.00, 33.61, 30.25, 25.42, 24.16, 21.90, 18.80, 16.78. MS (ESI) *m/z* 538.72 ([M − H]^−^).

*N’-(3-Bromobenzylidene)-2-(dehydroabietyloxy)acetohydrazide* (**4l**): yellow solid; yield 61%; m.p. 178.3–179.8 °C. IR (KBr, cm^−1^): 3554, 3414, 3194 (N–H), 3111, 2947, 1730 (C=O), 1691 (C=O), 1560, 1409, 1236, 1170, 1120, 960, 877, 821, 734, 678. ^1^H-NMR (600 MHz, CDCl_3_) δ 9.85 (s, 1H, CONH), 7.64 (s, 1H, N=CH), 7.50 (dd, *J* = 21.1, 7.6 Hz, 1H, H-6’), 7.41 (d, *J* = 14.0 Hz, 1H, H-2’), 7.24 (s, 1H, H-4’), 7.20 (d, *J* = 8.1 Hz, 1H, H-5’), 7.02 (d, *J* = 7.9 Hz, 1H, H-11), 6.90 (s, 1H, H-14), 6.82 (s, 1H, H-12), 5.11–5.15 (m, 2H, H-21), 2.95–2.98 (m, 1H, H-15), 2.80–2.93 (m, 2H, H-7), 2.38 (d, *J* = 12.3 Hz, 1H, H_e_-1), 2.34 (d, *J* = 12.7 Hz, 1H, H-5), 1.47–1.96 (m, 7H, H_a_-1, H-2, H-3 and H-6), 1.37 (s, 3H, H-19), 1.26 (s, 3H, H-20), 1.22 (d, *J* = 6.9 Hz, 6H, H-16 and H-17). ^13^C-NMR (151 MHz, CDCl_3_) δ 178.38 (C=O), 170.05 (C=O), 149.58, 147.07, 145.85, 138.47, 135.04, 132.22, 130.45, 129.89, 127.11, 125.69, 124.33, 124.02, 123.15, 61.80, 47.79, 44.80, 38.13, 37.10, 36.93, 33.61, 30.26, 25.42, 24.18, 21.82, 18.80, 16.78. MS (ESI) *m/z* 536.77 ([M − H]^−^).

*N’-(4-Bromobenzylidene)-2-(dehydroabietyloxy)acetohydrazide* (**4m**): White solid; yield 90%, m.p. 188.1–188.6 °C. IR (KBr, cm^−1^): 3201 (N–H), 3101, 2949, 1734 (C=O), 1691 (C=O), 1415, 1309, 1236, 1118, 1064, 1006, 952, 889, 817. ^1^H-NMR (600 MHz, CDCl_3_) δ 10.51 (s, 1H, CONH), 7.75 (s, 1H, N=CH), 7.51 (d, *J* = 8.5 Hz, 2H, H-3’ and H-5’), 7.46 (d, *J* = 8.5 Hz, 2H, H-2’ and H-6’), 7.20 (d, *J* = 8.2 Hz, 1H, H-11), 7.02 (d, *J* = 9.3 Hz, 1H, H-12), 6.86 (s, 1H, H-14), 5.14 (q, *J* = 16.0 Hz, 2H, H-21), 2.94–3.02 (m, 1H, H-15), 2.79–2.90 (m, 2H, H-7), 2.38 (dd, *J* = 12.4, 1.7 Hz, 1H, H_e_-1), 2.34 (d, *J* = 12.8 Hz, 1H, H-5), 1.50–1.98 (m, 7H, H_a_-1, H-2, H-3 and H-6), 1.37 (s, 3H, H-19), 1.26 (s, 3H, H-20), 1.22 (d, *J* = 6.9 Hz, 6H, H-16 and H-17). ^13^C-NMR (151 MHz, CDCl_3_) δ 178.50 (C=O), 170.04 (C=O), 147.01, 145.88, 144.20, 134.92, 132.51, 132.28, 128.70, 127.09, 124.79, 124.34, 124.06, 61.47, 47.84, 44.88, 38.10, 37.10, 36.93, 33.60, 30.28, 25.43, 24.15, 21.80, 18.77, 16.79. MS (ESI) *m/z* 538.73 ([M − H]^−^).

*N’-(2-Methoxybenzylidene)-2-(dehydroabietyloxy)acetohydrazide (***4n***):* White solid; yield 84%; m.p. 190.3–191.8 °C. IR (KBr, cm^−1^): 3442, 3217 (N-H), 3084, 2958, 1730 (C=O), 1691 (C=O), 1666, 1600, 1462, 1361, 1298, 1247, 1176, 1126, 1080, 1024, 968, 819, 761, 644. ^1^H-NMR (600 MHz, CDCl_3_) δ 10.38 (s, 1H, CONH), 8.24 (s, 1H, N=CH), 7.88 (d, *J* = 7.6 Hz, 1H, H-6’), 7.38 (t, *J* = 7.8 Hz, 1H, H-4’), 7.20 (d, *J* = 8.2 Hz, 1H, H-5’), 6.97–7.05 (d, 2H, H-11 and H-3’), 6.91 (d, *J* = 8.4 Hz, 1H, H-14), 6.87 (s, 1H, H-12), 5.18 (s, 2H, H-21), 3.85 (s, 3H, OCH_3_), 2.96–3.06 (m, 1H, H-15), 2.79–2.83 (m, 2H, H-7), 2.40 (d, *J* = 11.9 Hz, 1H, H_e_-1), 2.34 (d, *J* = 12.5 Hz, 1H, H-5), 1.48–1.98 (m, 7H, H_a_-1, H-2, H-3 and H-6), 1.37 (s, 3H, H-19), 1.26 (s, 3H, H-20), 1.23 (d, *J* = 6.9 Hz, 6H, H-16 and H-17). ^13^C-NMR (151 MHz, CDCl_3_) δ 178.49 (C=O), 169.74 (C=O), 158.21, 147.09, 145.79 141.27, 135.19, 131.75, 127.15, 126.40, 124.31, 123.94, 122.21, 120.96, 111.18, 61.63, 55.62, 47.84, 44.91, 38.15, 37.12, 36.89, 33.61, 30.24, 25.47, 24.17, 21.76, 18.83 (C-6), 16.77. MS (ESI) *m*/*z* 488.86 ([M − H]^−^).

*N’-(3-Methoxybenzylidene)-2-(dehydroabietyloxy)acetohydrazide* (**4o**): light yellow solid; yield 81%; m.p. 136.7–137.9 °C. IR (KBr, cm^−1^): 3412, 3080, 2958, 2927, 2870, 1726 (C=O), 1685 (C=O), 1573, 1458, 1419, 1311, 1286, 1249, 1130, 1043, 948, 873, 823, 775, 688. ^1^H-NMR (600 MHz, CDCl_3_) δ 10.71 (s, 1H, CONH), 7.80 (s, 1H, N=CH), 7.31 (t, 1H, H-6’), 7.21 (d, *J* = 13.2 Hz, 2H, H-2’ and H-5’), 7.17 (d, *J* = 7.6 Hz, 1H, H-11), 7.03 (d, *J* = 8.0 Hz, 1H, H-12), 6.97 (d, *J* = 10.5 Hz, 1H, H-4’), 6.89 (s, 1H, H-14), 5.18 (q, *J* = 16.0 Hz, 2H, H-21), 3.85 (s, 3H, OCH_3_), 2.99–3.05 (s, 1H, H-15), 2.81–2.92 (m, 2H, H-7), 2.42 (dd, *J* = 12.3, 1.8 Hz, 1H, H_e_-1), 2.36 (d, *J* = 12.8 Hz, 1H, H-5), 1.50–2.00 (m, 7H, H_a_-1, H-2, H-3 and H-6), 1.37 (s, 3H, H-19), 1.26 (s, 3H, H-20), 1.23 (d, *J* = 6.9 Hz, 6H, H-16 and H-17). ^13^C-NMR (151 MHz, CDCl_3_) δ 178.51 (C=O), 170.15 (C=O), 160.02, 147.06, 145.85, 145.41, 135.01, 129.98, 127.13, 124.34, 124.02, 120.55, 116.66, 111.53, 61.51, 55.47, 47.86, 44.86, 38.13, 37.11, 36.99, 33.62, 30.30, 25.45, 24.16, 21.83, 18.81, 16.81. MS (ESI) *m*/*z* 488.91 ([M − H]^−^).

*N’-(4-Methoxybenzylidene)-2-(dehydroabietyloxy)acetohydrazide* (**4p**): White solid; yield 90%; m.p. 174.8–175.5 °C. IR (KBr, cm^−1^): 2931, 1737 (C=O), 1689 (C=O), 1604, 1504, 1419, 1303, 1247, 1168, 1124, 1035, 954, 893, 837. ^1^H-NMR (600 MHz, CDCl_3_) δ 10.49 (s, 1H, CONH), 7.76 (s, 1H, N=CH), 7.56 (d, 2H, H-2’, H-6’), 7.20 (d, 1H, H-11), 7.01 (d, 1H, H-12), 6.92 (d, 2H, H-3’, H-5’), 6.88 (s, 1H, H-14), 5.16 (q, *J* = 15.9 Hz, 2H, H-21), 3.85 (s, 3H, OCH_3_), 2.98–3.04 (m, 1H, H-15), 2.81–2.90 (m, 2H, H-7), 2.41 (d, *J* = 11.8 Hz, 1H, H_e_-1), 2.35 (d, *J* = 12.8 Hz, 1H, H-5), 1.53–1.98 (m, 7H, H_a_-1, H-2, H-3 and H-6), 1.39 (s, 3H, H-19), 1.27 (s, 3H, H-20), 1.23 (d, *J* = 6.9, Hz, 6H, H-16 and H-17).^13^C-NMR (151 MHz, CDCl_3_) δ 178.49 (C=O), 169.80 (C=O), 161.51, 147.08, 145.82, 145.18, 135.05, 128.90, 127.12, 126.41, 124.34, 124.00, 114.38, 61.56, 55.53, 47.84, 44.88, 38.13, 37.11, 36.95, 33.61, 30.31, 25.45, 24.16, 21.80, 18.80, 16.80. MS (ESI) *m*/*z* 488.88 ([M − H]^−^).

*Dehydroabietic acid-based 2-fluorophenyl acylhydrazone* (**4q**): light yellow solid; yield 72%; m.p. 130.2–130.5 °C. IR (KBr, cm^−1^): 2954, 2868, 1730 (C=O), 1691 (C=O), 1415, 1311, 1240, 1170, 1126, 1053, 877, 823, 756, 630. ^1^H-NMR (600 MHz, CDCl_3_) δ 10.86 (s, 1H, CONH), 8.10 (s, 1H, N=CH), 7.89 (d, *J* = 7.8 Hz, 1H, H-6’), 7.38 (d, *J* = 7.9 Hz, 1H, H-3’), 7.21 (d, *J* = 7.6 Hz, 1H, H-5’), 7.18 (d, *J* = 8.9 Hz, 1H, H-11), 7.12 (d, *J* = 8.2 Hz, 1H, H-4’), 7.02 (d, *J* = 9.3 Hz, 1H, H-12), 6.89 (s, 1H, H-14), 5.17 (q, *J* = 16.0 Hz, 2H, H-21), 2.95–3.05 (m, 1H, H-15), 2.80–2.93 (m, 2H, H-7), 2.41 (dd, *J* = 12.4, 1.7 Hz, 1H, H_e_-1), 2.35 (d, *J* = 12.9 Hz, 1H, H-5), 1.50–1.98 (m, 7H, H_a_-1, H-2, H-3 and H-6), 1.37 (s, 3H, H-19), 1.26 (s, 3H, H-20), 1.23 (d, *J* = 6.9 Hz, 6H, H-16 and H-17). ^13^C-NMR (151 MHz, CDCl_3_) δ 178.59 (C=O), 170.29 (C=O), 162.42, 147.14, 145.78, 138.62, 135.14, 132.02, 127.12, 126.72, 124.59, 124.34, 123.97, 121.60, 116.17, 61.44, 47.87, 44.78, 38.13, 37.10, 37.01, 33.62, 30.23, 25.43, 24.18, 21.79, 18.77, 16.72. MS (ESI) *m/z* 476.88 ([M − H]^−^).

*N’-(3-Fluorobenzylidene)-2-(dehydroabietyloxy)acetohydrazide* (**4r**): White solid; yield 82%, m.p. 166.0–167.3 °C. IR (KBr, cm^−1^): 3414, 3107, 2954, 1732 (C=O), 1697 (C=O), 1610, 1579, 1450, 1411, 1236, 1124, 943, 866, 785, 684. ^1^H-NMR (600 MHz, CDCl_3_) δ 10.75 (s, 1H, CONH), 7.80 (s, 1H, N=CH), 7.39 (d, *J* = 9.2 Hz, 1H, H-5’), 7.35 (d, *J* = 5.3 Hz, 1H, H-2’), 7.34 (s, 1H, H-4’), 7.21 (d, *J* = 8.2 Hz, 1H, H-11), 7.10 (t, *J* = 7.7 Hz, 1H, H-5’), 7.02 (d, *J* = 7.9 Hz, 1H, H-12), 6.88 (s, 1H, H-14), 5.16 (q, *J* = 16.0 Hz, 2H, H-21), 2.94–3.07 (m, 1H, H-15), 2.78–2.93 (m, 2H, H-7), 2.40 (dd, *J* = 12.2, 1.2 Hz, 1H, H_e_-1), 2.36 (d, *J* = 12.9 Hz, 1H, H-5), 1.51–2.00 (m, 7H, H_a_-1, H-2, H-3 and H-6), 1.39 (s, 3H, H-19), 1.27 (s, 3H, H-20), 1.22 (d, *J* = 6.8 Hz, 6H, H-16 and H-17). ^13^C-NMR (151 MHz, CDCl_3_) δ 178.51 (C=O), 170.31 (C=O), 163.96, 147.04, 145.88, 144.17, 135.92, 134.94, 130.58, 127.10, 124.35, 124.06, 123.67, 117.45, 113.20, 61.46, 47.85, 44.87, 38.11, 37.10, 36.96, 33.61, 30.29, 25.43, 24.13, 21.81, 18.78, 16.80. MS (ESI) *m/z* 476.89 ([M − H]^−^).

*N’-(4-Fluorobenzylidene)-2-(dehydroabietyloxy)acetohydrazide* (**4s**): White solid; yield 83%; m.p. 169.2–170.5 °C. IR (KBr, cm^−1^): 3224 (N–H), 3070, 2866, 1726 (C=O), 1687 (C=O), 1598, 1550, 1417, 1238, 1172, 1128, 1080, 945, 837, 723. ^1^H-NMR (600 MHz, CDCl_3_) δ 10.70 (s, 1H, CONH), 7.80 (s, 1H, N=CH), 7.61 (t, *J* = 8.3 Hz, 2H, H-2’ and H-6’), 7.21 (d, *J* = 8.1 Hz, 1H, H-11), 7.07 (t, 2H, *J* = 8.2 Hz, H-3’ and H-5’), 7.03 (d, *J* = 8.2 Hz, 1H, H-12), 6.87 (s, 1H, H-14), 5.16 (q, *J* = 16.0 Hz, 2H, H-21), 2.95–3.06 (m, 1H, H-15), 2.78–2.90 (m, 2H, H-7), 2.40 (d, *J* = 12.3 Hz, 1H, H_e_-1), 2.35 (d, *J* = 12.9 Hz, 1H, H-5), 1.49–2.00 (m, 7H, H_a_-1, H-2, H-3 and H-6), 1.39 (s, 3H, H-19), 1.27 (s, 3H, H-20), 1.23 (d, *J* = 6.9 Hz, 6H, H-16 and H-17). ^13^C-NMR (151 MHz, CDCl_3_) δ 178.51 (C=O), 170.13 (C=O), 164.94, 147.04, 145.89, 144.37, 134.94, 129.89, 129.24, 127.10, 124.35, 124.07, 116.21, 61.50, 47.84, 44.90, 38.12, 37.11, 36.96, 33.61, 30.30, 25.44, 24.13, 21.81, 18.79, 16.80. MS (ESI) *m/z* 476.84 ([M − H]^−^).

*N’-(2,3-Difluorobenzylidene)-2-(dehydroabietyloxy)acetohydrazide* (**4t**): White solid; yield 62%; m.p. 150.2–153.5 °C. IR (KBr, cm^−1^): 3097, 2966, 2868, 1732 (C=O), 1697 (C=O), 1612, 1487, 1415, 1305, 1238, 1170, 1124, 989, 786. ^1^H-NMR (600 MHz, CDCl_3_) δ 10.00 (s, 1H, CONH), 8.02 (s, 1H, H-23), 7.63 (dd, *J* = 7.5, 6.2 Hz, 1H, H-6’), 7.22 (dd, *J* = 20.1, 8.8 Hz, 2H, H-4’ and H-5’), 7.13 (dd, *J* = 12.4, 7.9 Hz, 1H, H-11), 7.02 (dd, *J* = 8.1, 1.5 Hz, 1H, H-12), 6.90 (s, 1H, H-14), 5.15 (q, *J* = 16.0 Hz, 2H, H-21), 2.96–3.04 (m, 1H, H-15), 2.80–2.93 (m, 2H, H-7), 2.39 (dd, *J* = 12.4, 1.9 Hz, 1H, H_e_-1), 2.34 (d, *J* = 12.9 Hz, 1H, H-5), 1.53–1.95 (m, 7H, Ha-1, H-2, H-3 and H-6), 1.38 (s, 3H, H-19), 1.27 (s, 3H, H-20), 1.24 (d, *J* = 6.9 Hz, 6H, H-16 and H-17). ^13^C-NMR (151 MHz, CDCl_3_) δ 178.36 (C=O), 169.52 (C=O), 151.49, 150.36, 148.76, 146.95, 145.67, 136.87, 134.91, 126.95, 124.35, 124.15, 123.83, 121.39, 118.73, 61.23, 47.73, 44.68, 37.97, 36.96, 36.80, 33.46, 30.05, 25.23, 23.97, 21.64, 18.59, 16.57. MS (ESI) *m/z* 495.1 ([M − H]^−^).

*N’-(2,4-Difluorobenzylidene)-2-(dehydroabietyloxy)acetohydrazide* (**4u**): White solid; yield 77%; m.p. 181.9–184.5 °C. IR (KBr, cm^−1^): 3072, 2949, 2870, 1737 (C=O), 1691 (C=O), 1608, 1494, 1419, 1298, 1274, 1230, 1170, 1126, 962, 854, 794. ^1^H-NMR (600 MHz, CDCl_3_) δ 9.80 (s, 1H, CONH), 7.97 (s, 1H, H-23), 7.89 (dd, *J* = 14.9, 8.4 Hz, 1H and H-6’), 7.20 (d, *J* = 8.2 Hz, 1H, H-11), 7.02 (d, *J* = 8.0 Hz, 1H, H-12), 6.95 (t, *J* = 9.2 Hz, 1H, C-14), 6.92–6.83 (m, 2H, H-3’and H-5’), 5.14 (q, *J* = 16.0 Hz, 2H, H-21), 2.94–3.04 (m, 1H, H-15), 2.78–2.93 (m, 2H, C-7), 2.39 (dd, *J* = 12.3, 1.7 Hz, 1H, H_e_-1), 2.34 (d, *J* = 13.0 Hz, 1H, H-5), 2.00–1.53 (m, 7H, Ha-1, H-2, H-3 and H-6), 1.38 (s, 3H, H-19), 1.27 (s, 3H, H-20), 1.24 (d, *J* = 6.9 Hz, 6H, H-16 and H-17), ^13^C-NMR (151 MHz, CDCl_3_) δ 178.34 (C=O), 169.28 (C=O), 162.43, 160.74, 154.86, 146.95, 145.67, 137.02, 134.94, 126.95, 124.15, 123.83, 117.72, 112.44, 104.29, 61.26, 47.72, 44.68, 37.97, 36.96, 36.78, 33.46, 30.06, 25.24, 23.99, 21.63, 18.60, 16.57. MS (ESI) *m/z* 495.2 ([M − H]^−^).

*N’-(2,6-Difluorobenzylidene)-2-(dehydroabietyloxy)acetohydrazide* (**4v**): Brown solid;yield 93%; m.p. 107.3–110.0 °C. IR (KBr, cm^−1^): 3101, 2954, 2868, 1728, 1693, 1620, 1467, 1415, 1240, 1170, 1126, 1012, 881, 785. ^1^H-NMR (600 MHz, CDCl_3_) δ 9.80 (s, 1H, CONH), 7.97 (s, 1H, H-23), 7.89 (dd, *J* = 14.9, 8.4 Hz, 1H, H-4’), 7.20 (d, *J* = 8.2 Hz, 1H, H-11), 7.02 (d, *J* = 8.0 Hz, 1H, H-12), 6.95 (t, *J* = 9.2 Hz, 1H, H-14), 6.88 (q, *J* = 8.5 Hz, 2H, H-3’ and H-5’), 5.14 (q, *J* = 16.0 Hz, 2H, H-21), 2.94–3.04 (m, 1H, H-15), 2.78–2.94 (m, 2H, H-7), 2.39 (dd, *J* = 12.3, 1.7 Hz, 1H, H_e_-1), 2.34 (d, *J* = 13.0 Hz, 1H, H-5), 1.54–2.00 (m, 7H, Ha-1, H-2, H-3 and H-6), 1.38 (s, 3H, H-19), 1.27 (d, *J* = 7.1 Hz, 3H, H-20), 1.24 (d, *J* = 6.9 Hz, 6H, H-16 and H-17). ^13^C-NMR (151 MHz, CDCl_3_) δ 178.29 (C=O), 170.00 (C=O), 162.04, 160.34, 153.60, 147.00, 145.62, 134.94, 131.20, 126.96, 124.14, 123.78, 117.72, 112.44, 104.29, 61.27, 47.68, 44.67, 37.98, 36.96, 36.78, 33.46, 30.06, 25.25, 23.97, 21.59, 18.61, 16.57. MS (ESI) *m/z* 495.2 ([M − H]^−^).

*N’-(3,5-Difluorobenzylidene)-2-(dehydroabietyloxy)acetohydrazide* (**4w**): White solid; yield 72%; m.p. 182.6–186.6 °C. IR (KBr,cm^−1^): 3091, 2949, 2885, 1724 (C=O), 1703 (C=O), 1612, 1583, 1436, 1415, 1363, 1298, 1240, 1174, 1122, 983, 850, 752.^1^H-NMR (600 MHz, CDCl_3_) δ 10.02 (s, 1H, CONH), 7.71 (s, 1H, H-23), 7.20 (d, *J* = 8.2 Hz, 1H, H-11), 7.16 (d, *J* = 5.8 Hz, 2H, H-2’ and H-6’), 7.02 (d, *J* = 8.0 Hz, 1H, H-12), 6.89 (s, 1H, H-14), 6.87 (t, *J* = 8.6 Hz, 1H, H-4’), 5.14 (q, *J* = 16.0 Hz, 2H, H-21), 2.96 –3.05 (m, 1H, H-15), 2.80–2.93 (m, 2H, H-7), 2.39 (d, *J* = 12.6, Hz, 1H, H_e_-1), 2.36 (d, *J* = 13.0 Hz, 1H, H-5),1.53–1.97 (m, 7H, Ha-1, H-2, H-3 and H-6), 1.38 (s, 3H, H-19), 1.27 (s, 3H, H-20), 1.23 (d, *J* = 6.9 Hz, 6H, H-16 and H-17). ^13^C-NMR (151 MHz, CDCl_3_) δ 178.31 (C=O), 169.65 (C=O), 164.00, 162.43, 162.35, 146.88, 145.74, 142.28, 136.58, 134.79, 126.94, 124.15, 123.89, 109.95, 105.67, 61.22, 47.72, 44.73, 37.97, 36.96, 36.77, 33.45, 30.08, 25.24, 23.97, 21.64, 18.60, 16.62. MS (ESI) *m/z* 495.1 ([M − H]^−^).

*N’-((Pyridine-3-yl)methylene)-2-(dehydroabietyloxy)acetohydrazide* (**4x**): White solid; yield 85%; m.p. 191.9–193.8 °C. IR (KBr, cm^−1^): 3084, 2956, 2866, 1723 (C=O), 1710 (C=O), 1614, 1462, 1402, 1288, 1240, 1170, 1126, 887, 704. ^1^H-NMR (600 MHz, CDCl_3_) δ 10.34 (s, 1H, CONH), 8.80 (s, 1H, H-2’), 8.65 (d, *J* = 4.1 Hz, 1H, H-4’), 8.00 (d, *J* = 7.9 Hz, 1H, H-6’), 7.83 (s, 1H, H-23), 7.35 (dd, *J* = 7.8, 4.9 Hz, 1H, H-5’), 7.20 (d, *J* = 8.2 Hz, 1H, H-11), 7.02 (d, *J* = 8.1 Hz, 1H, H-12), 6.88 (s, 1H, H-14), 5.16 (q, *J* = 16.0 Hz, 2H, H-21), 2.97–3.04 (m, 1H, H-15), 2.80–2.92 (m, 2H, H-7), 2.39 (d, *J* = 11.2 Hz, 1H, H_e_-1), 2.35 (d, *J* = 12.8 Hz, 1H, H-5), 1.53–1.94 (m, 7H, Ha-1, H-2, H-3 and H-6), 1.39 (s, 3H, H-19), 1.27 (s, 3H, H-20), 1.23 (d, *J* = 6.9 Hz, 6H, H-16 and H-17). ^13^C-NMR (151 MHz, CDCl_3_) δ 178.34 (C=O), 169.75 (C=O), 151.13, 149.01, 146.89, 145.73, 141.81, 134.78, 133.50, 129.41, 126.94, 124.16, 123.89, 123.77, 61.26, 47.72, 44.73, 37.97, 36.96, 36.80, 33.46, 30.09, 25.24, 23.99, 21.66, 18.62, 16.63. MS (ESI) *m/z* 460.2 ([M − H]^−^).

### 3.5. Cytotoxicity Assay In Vitro

The CNE-2, HepG2, HeLa, and BEL-7402 cell lines used in this work were all purchased from the Institute of Biochemistry and Cell Biology, China Academy of Sciences. All were supplemented with 10% heat-inactivated fetal bovine serum in a humidified atmosphere of 5% CO_2_/95% air at 37 °C. In order to investigate the potential of compounds **4**, cisplatin, a commercial classical anticancer drug was used as a reference drug. CNE-2, HepG2, HeLa, and BEL-7402 cells were seeded into 96-well microculture plates and allowed to adhere for 24 h, respectively. After cells were exposed to compounds at concentrations from 100 to 0.1 μM for 48 h, medium was aspirated and replenished with complete medium. IC_50_ values was evaluated by MTT tetrazolium dye assay. All the tests were repeated in at least three independent experiments. The IC_50_ values of the compounds were calculated using SPSS Version 10 software (IBM, New York, NY, USA), which defined the IC_50_ value as the concentration required to inhibit cell growth by 50%.

## 4. Conclusions

This study started with the aim to explore the potential anticancer activity of dualistic molecules bearing a combination of the dehydroabietic acid and acylhydrazone moieties. Their cytotoxic activities against human nasopharyngeal carcinoma (CNE-2), human liver carcinoma (HepG2), human cervix carcinoma (HeLa), and human hepatocellular carcinoma (BEL-7402) cells lines, along with HL-7702, a normal human liver cell line, were investigated. A number of compounds showed moderate to high anticancer activity and the results revealed that the introduction of acylhydrazone on the skeleton of DHA markedly improved the anticancer activity. In particular, compounds **4k** and **4x** inhibited the growth of HepG2 and HeLa cell lines with low (<10 μM) micromolar IC_50_ values. Compound **4w** was found to exhibit anticancer activity against HeLa and BEL-7402 cell lines compared to cisplatin as reference drug. In addition, compounds **4i** and **4l** displayed good cytotoxic activities against CNE-2 cells (IC_50_ values of 12.61 μM and 11.45 μM, respectively). The results highlight these novel dehydroabietic acid derivatives as potential leads for the further investigation for new anticancer drug candidates.

## Figures and Tables

**Table 1:** Effect of compounds **4a**–**x** against cell viability of different cell lines.

Compound	IC_50_ (μM)				
	CNE-2	HepG2	HeLa	BEL-7402	HL-7702
**4a**	28.20 ± 0.43	27.81 ± 0.58	17.89 ± 0.13	36.70 ± 0.34	>100
**4b**	17.22 ± 0.21	12.44 ± 0.31	6.72 ± 0.22	15.38 ± 0.08	68.58 ± 2.14
**4c**	15.82 ± 0.56	27.60 ± 0.23	10.84 ± 0.43	21.03 ± 0.09	53.22 ± 1.36
**4d**	38.68 ± 0.49	35.26 ± 0.11	12.22 ± 0.08	33.59 ± 0.38	50.74 ± 1.82
**4e**	48.85 ± 0.05	7.62 ± 0.32	34.92 ± 0.16	15.60 ± 0.44	63.04 ± 3.06
**4f**	51.39 ± 0.34	25.61 ± 0.35	33.23 ± 0.21	16.90 ± 0.41	61.90 ± 1.34
**4g**	60.42 ± 1.02	12.50 ± 0.46	27.26 ± 0.50	36.40 ± 0.74	>100
**4h**	98.78 ± 0.45	44.64 ± 0.92	15.20 ± 0.29	38.53 ± 0.52	>100
**4i**	12.61 ± 0.24	57.84 ± 0.75	13.91 ± 0.41	44.65 ± 0.21	77.08 ± 0.96
**4j**	93.26 ± 0.52	39.50 ± 0.82	13.06 ± 0.08	27.45 ± 0.17	>100
**4k**	47.70 ± 0.23	8.47 ± 0.55	5.53 ± 0.04	48.16 ± 0.32	65.94 ± 1.48
**4l**	11.45 ± 0.11	35.05 ± 0.96	25.14 ± 0.10	19.95 ± 0.27	86.44 ± 3.12
**4m**	89.66 ± 0.27	9.94 ± 0.59	12.84 ± 0.15	23.08 ± 0.33	>100
**4n**	26.08 ± 0.05	50.08 ± 0.74	45.13 ± 0.09	23.79 ± 0.51	70.37 ± 2.23
**4o**	86.46 ± 0.13	51.28 ± 0.63	32.06 ± 0.21	14.77 ± 0.38	>100
**4p**	56.98 ± 0.08	48.59 ± 0.09	34.33 ± 0.32	55.29 ± 0.44	91.75 ± 1.57
**4q**	27.44 ± 0.72	93.76 ± 0.39	27.71 ± 0.14	22.79 ± 0.34	>100
**4r**	51.02 ± 0.94	31.57 ± 0.47	12.82 ± 0.13	25.86 ± 0.53	68.59 ± 1.25
**4s**	49.81 ± 0.49	90.08 ± 0.33	28.79 ± 0.24	25.22 ± 0.22	>100
**4t**	33.34 ± 0.56	22.89 ± 0.04	11.14 ± 0.15	18.78 ± 0.59	72.04 ± 1.66
**4u**	32.83 ± 0.16	25.36 ± 0.54	5.86 ± 0.09	18.64 ± 0.37	55.80 ± 1.38
**4v**	26.77 ± 0.46	16.90 ± 0.18	14.96 ± 0.21	20.52 ± 0.53	44.63 ± 2.02
**4w**	15.44 ± 0.21	12.07 ± 0.07	2.21 ± 0.04	14.46 ± 0.22	66.08 ± 1.84
**4x**	12.96 ± 0.13	8.07 ± 0.25	4.94 ± 0.13	15.83 ± 0.18	58.62 ± 2.62
**DHA**	88.64 ± 0.73	80.36 ± 0.84	37.40 ± 0.64	46.70 ± 0.55	>100
**Cisplatin**	8.75 ± 0.24	6.42 ± 0.18	1.94 ± 0.20	12.68 ± 0.33	20.76 ± 0.83

## Supplementary Materials

Supplementary materials are available online.

## References

[1] Xue S.A., Stauss H.J. (2007). Enhancing immune responses for cancer therapy. Cell. Mol. Immunol..

[2] Malvezzi M., Carioli G., Rodriguez T., Negri E., La Vecchia C. (2016). Global trends and predictions in ovarian cancer mortality. Ann. Oncol..

[3] Chen W.Q., Zheng R.S., Baade P.D., Zhang S.W., Zeng H.M., Bray F., Jemal A., Yu X.Q., He J. (2016). Cancer statistics in China, 2015. CA Cancer J. Clin..

[4] Newman D.J., Cragg G.M., Snader K.M. (2003). Natural products as source of new drugs over the period. J. Nat. Prod..

[5] Lee K.H. (2010). Discovery and development of natural product derived chemo-therapeutic agents based on a medicinal chemistry approach. J. Nat. Prod..

[6] Chyu C.F., Lin H.C., Kuo Y.H. (2005). New abietane and seco-abietane diterpenes from the roots of *Taiwania cryptomerioides*. Chem. Pharm. Bull..

[7] Pertino M.W., Vega C., Rolón M., Coronel C., Arias A.R., Schmeda-irschmann G. (2017). Antiprotozoal activity of triazole derivatives of dehydroabietic acid and oleanolic acid. Molecules.

[8] Wada H., Kodato S., Kawamori M., Morikawa T., Nakai H., Takeda M., Saito S., Onoda Y., Tamaki H. (1985). Antiulcer activity of dehydroabietic acid derivatives. Chem. Pharm. Bull..

[9] Kang M.S., Hirai S., Goto T., Kuroyanagi K., Lee J.Y., Uemura T., Ezaki Y., Takahashi N., Kawada T. (2008). Dehydroabietic acid, a phytochemical, acts as ligand for PPARs in macrophages and adipocytes to regulate inflammation. Biochem. Biophys. Res. Commun..

[10] Zapata B., Rojas M., Betancur-Galvis L., Mesa-Arango A.C., Pérez-Guaitac D., González M.A. (2013). Cytotoxic, immunomodulatory, antimycotic, and antiviral activities of semisynthetic 14-hydroxyabietane derivatives and triptoquinone C-4 epimers. Med. Chem. Commun..

[11] Zhang W.M., Yang T., Pan X.Y., Liu X.L., Lin H.X., Gao Z.B., Yang C.G., Cui Y.M. (2017). The synthesis and antistaphylococcal activity of dehydroabietic acid derivatives: Modifications at C12 and C7. Eur. J. Med. Chem..

[12] Helfenstein A., Vahermo M., Nawrot D.A., Demirci F., Iscan G., Krogerus S., Yli-Kauhaluoma J., Moreira V.M., Tammela P. (2017). Antibacterial profiling of abietane-type diterpenoids. Bioorg. Med. Chem..

[13] Kusumoto1 N., Zhao T., Swedjemark G., Ashitani T., Takahashi K., Borg-Karlson A.K. (2014). Antifungal properties of terpenoids in *Picea abies* against *Heterobasidion parviporum*. For. Path..

[14] Tolmacheva I.A., Tarantin A.V., Boteva A.A., Anikina L.V., Vikharev Y.B., Grishko V.V., Tolstikov A.G. (2006). Synthesis and biological activity of nitrogen-containing derivatives of methyl dehydroabietate. Pharm. Chem. J..

[15] Kim J., Kang Y.G., Lee J.Y., Choi D.H., Cho Y.U., Shin J.M., Park J.S., Lee J.H., Kima W.G., Seo D.B. (2015). The natural phytochemical dehydroabietic acid is an anti-aging reagent that mediates the direct activation of SIRT1. Mol. Cell. Endocrinol..

[16] Sepúlveda B., Astudillo L., Rodríguez J.A., Yáñez T., Theoduloz C., Schmeda-Hirschmann G. (2005). Gastroprotective and cytotoxic effect of dehydroabietic acid derivatives. Pharmacol. Res..

[17] Cui Y.M., Liu X.L., Zhang W.M., Lin H.X., Ohwada T., Ido K., Sawada K. (2016). The synthesis and BK channel-opening activity of *N*-acylaminoalkyloxime derivatives of dehydroabietic acid. Bioorg. Med. Chem. Lett..

[18] Xu H.T., Liu L.L., Fan X.T., Zhang G.J., Li Y.C., Jiang B. (2017). Identification of a diverse synthetic abietane diterpenoid library for anticancer activity. Bioorg. Med. Chem. Lett..

[19] Wen G., Miao T.T., Hua D.W., Jin X.Y., Tao X.B., Huang C.B., Wang S.F. (2017). Synthesis and in vitro cytotoxic evaluation of new 1H-benzo[*d*]imidazole derivatives of dehydroabietic acid. Bioorg. Med. Chem. Lett..

[20] Huang X.C., Huang R.Z., Liao Z.X., Pan Y.M., Gou S.H., Wang H.S. (2016). Synthesis and pharmacological evaluation of dehydroabietic acid thiourea derivatives containing bisphosphonate moiety as an inducer of apoptosis. Eur. J. Med. Chem..

[21] Cukierman D.S., Pinheiro A.B., Castiñeiras-Filho S.L., Silva A.S., Miotto M.C., De Falco A., de P., Ribeiro T., Maisonette S., da Cunha A.L., Hauser-Davis R.A. (2017). A moderate metal-binding hydrazone meets the criteria for a bioinorganic approach towards Parkinson’s disease: Therapeutic potential, blood-brain barrier crossing evaluation and preliminary toxicological studies. J. Inorg. Biochem..

[22] Zhang F., Wang X.L., Shi J., Wang S.F., Yin Y., Yang Y.S., Zhang W.M., Zhu H.L. (2014). Synthesis, molecular modeling and biological evaluation of *N*-benzylidene-2-((5-(pyridin-4-yl)-1,3,4-oxadiazol-2-yl)thio)acetohydrazide derivatives as potential anticancer agents. Bioorg. Med. Chem..

[23] Onnis V., Demurtas M., Deplano A., Balboni G., Baldisserotto A., Manfredini S., Pacifico S., Liekens S., Balzarini J. (2016). Design, synthesis and evaluation of antiproliferative activity of new benzimidazolehydrazones. Molecules.

[24] Xia Y., Fan C.D., Zhao B.X., Shin D.S., Miao J.Y. (2008). Synthesis and structure–activity relationships of novel 1-arylmethyl-3-aryl-1*H*-pyrazole-5-carbohydrazide hydrazone derivatives as potential agents against A549 lung cancer cells. Eur. J. Med. Chem..

[25] Cui Z.N., Li Y., Ling Y., Huang J., Cui J.R., Wang R.Q., Yang X.L. (2010). New class of potent antitumor acylhydrazone derivatives containing furan. Eur. J. Med. Chem..

[26] Chen N.Y., Duan W.G., Lin G.S., Liu L.Z., Zhang R., Li D.P. (2016). Synthesis and antifungal activity of dehydroabietic acid-based 1,3,4-thiadiazole-thiazolidinone compounds. Mol. Divers..

[27] Chen N.Y., Duan W.G., Liu L.Z., Li F.Y., Lu M.P., Liu B.M. (2015). Synthesis and antifungal activity of dehydroabietic acid-based thiadiazole-phosphonates. Holzforschung.

[28] Lin G.S., Duan W.G., Yang L.X., Huang M., Lei F.H. (2017). Synthesis and antifungal activity of novel myrtenal-based 4-methyl-1,2,4-triazole-thioethers. Molecules.

[29] Duan W.G., Ma X.L., Mo Q.J., Huang J.X., Cen B., Xu X.T., Lei F.H. (2011). Synthesis and herbicidal activity of 5-dehydroabietyl-1,3,4-oxadiazolederivatives. Holzforschung.

[30] Mo Q.J., Duan W.G., Li X.R., Huang D.P., Luo Z.J. (2011). Synthesis and herbicidal activity of 2-substituted amino-5-dehydroabietyl-1,3,4-oxadiazole derivatives. Chin. J. Org. Chem..

[31] Li F.Y., Mo Q.J., Duan W.G., Lin G.S., Cen B., Chen N.Y., Yang Z.Q. (2014). Synthesis and insecticidal activities of *N*-(5-dehydroabietyl-1,3,4-thiadiazol-2-yl)-benzene-sulfonamides. Med. Chem. Res..

