# Supplementary material for: Synthesis and In Vitro Anticancer Activity of Novel Dehydroabietic Acid-Based Acylhydrazones
Source: Molecules. 2017 Jun 29;22(7):1087. doi: 10.3390/molecules22071087 (PMC6152134; doi:10.3390/molecules22071087)
Supplement: Supplementary file 1 [file molecules-22-01087-s001.pdf]

Synthesis and in Vitro Anticancer Activity of Novel Dehydroabietic Acid-Based Acylhydrazones

Fang-Yao Li , Xiu Wang, Wen-Gui Duan , Gui-Shan Lin

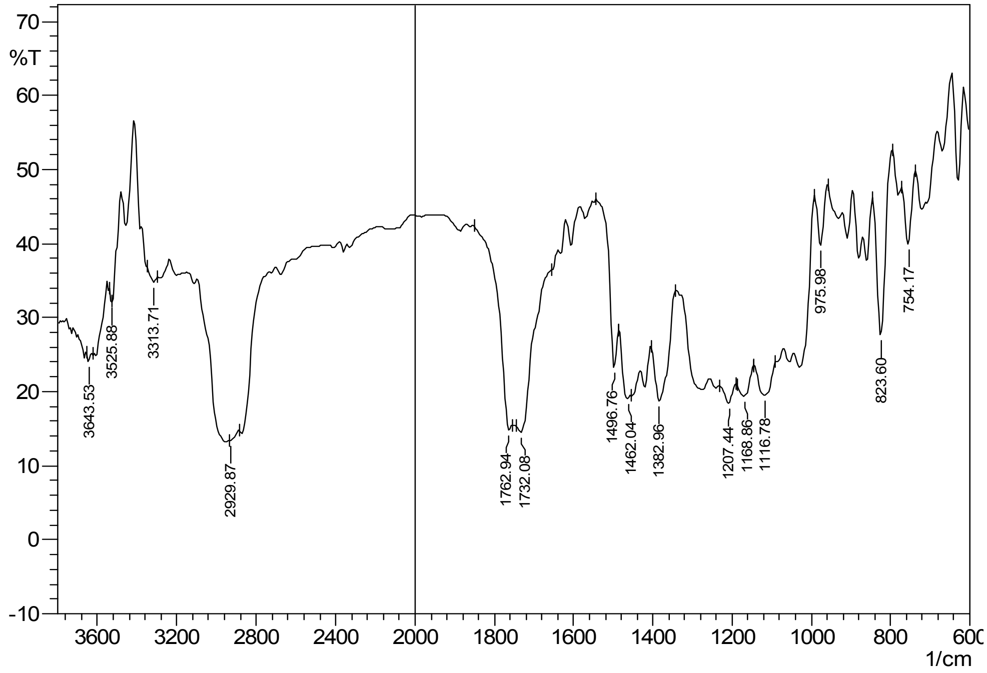

Figure S1. FTIR spectrum of dehydroabietic acid-based ethyl acetate (**2**)

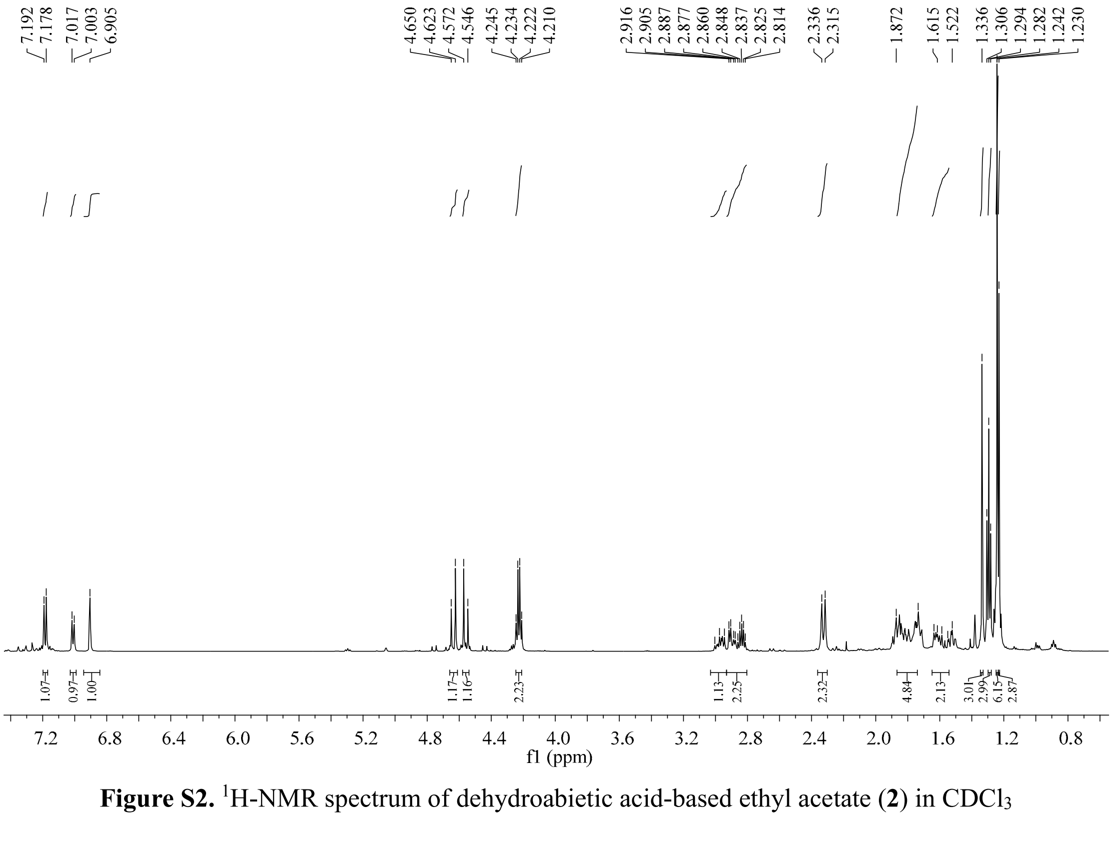

Figure S2. ^{1}H-NMR spectrum of dehydroabietic acid-based ethyl acetate (**2**) in $CDCl_3$

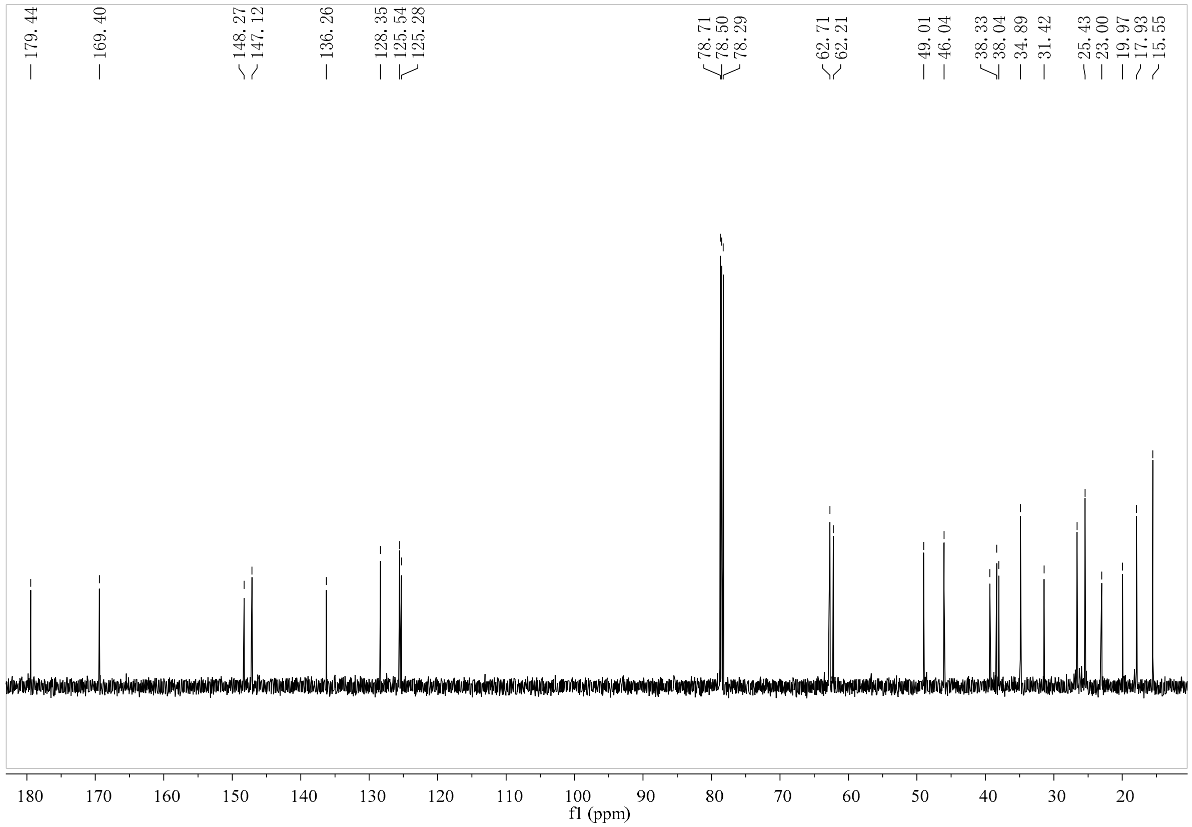

Figure S3. ^{13}C-NMR spectrum of dehydroabietic acid-based ethyl acetate (**2**) in $CDCl_3$

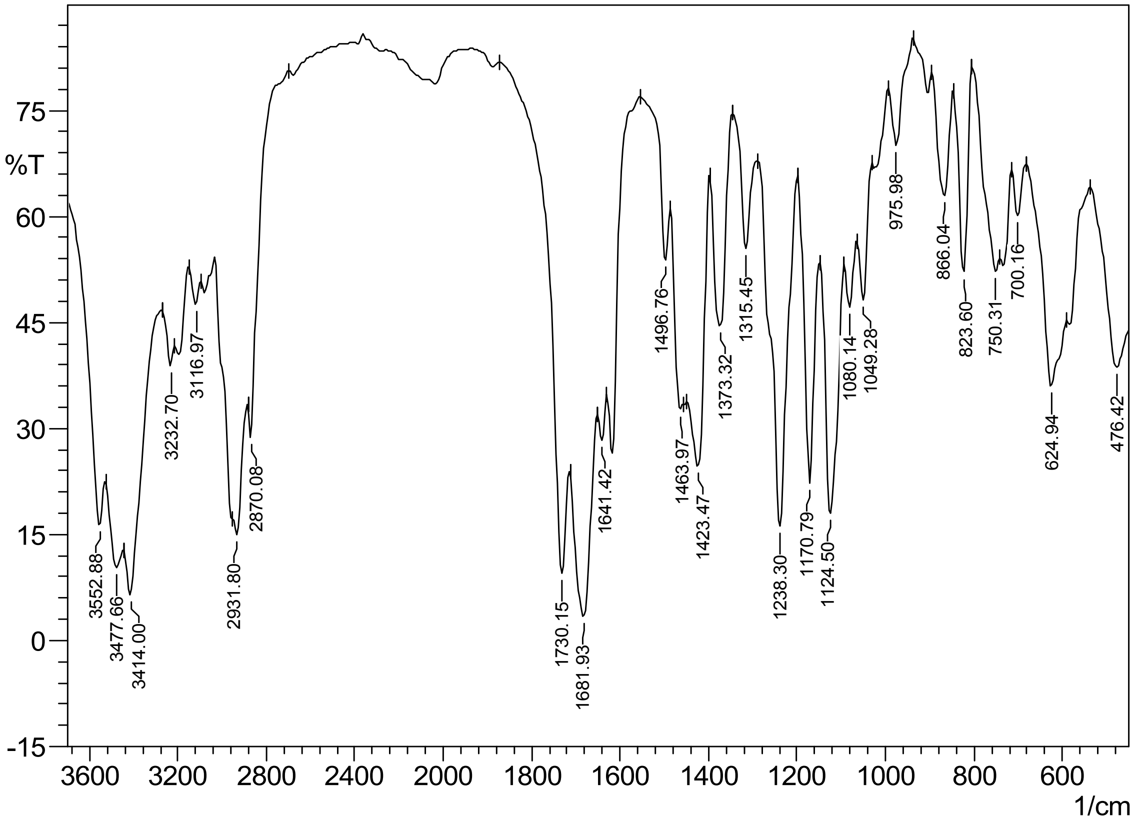

Figure S4. FTIR spectrum of dehydroabietic acid-based hydrazide (**3**)

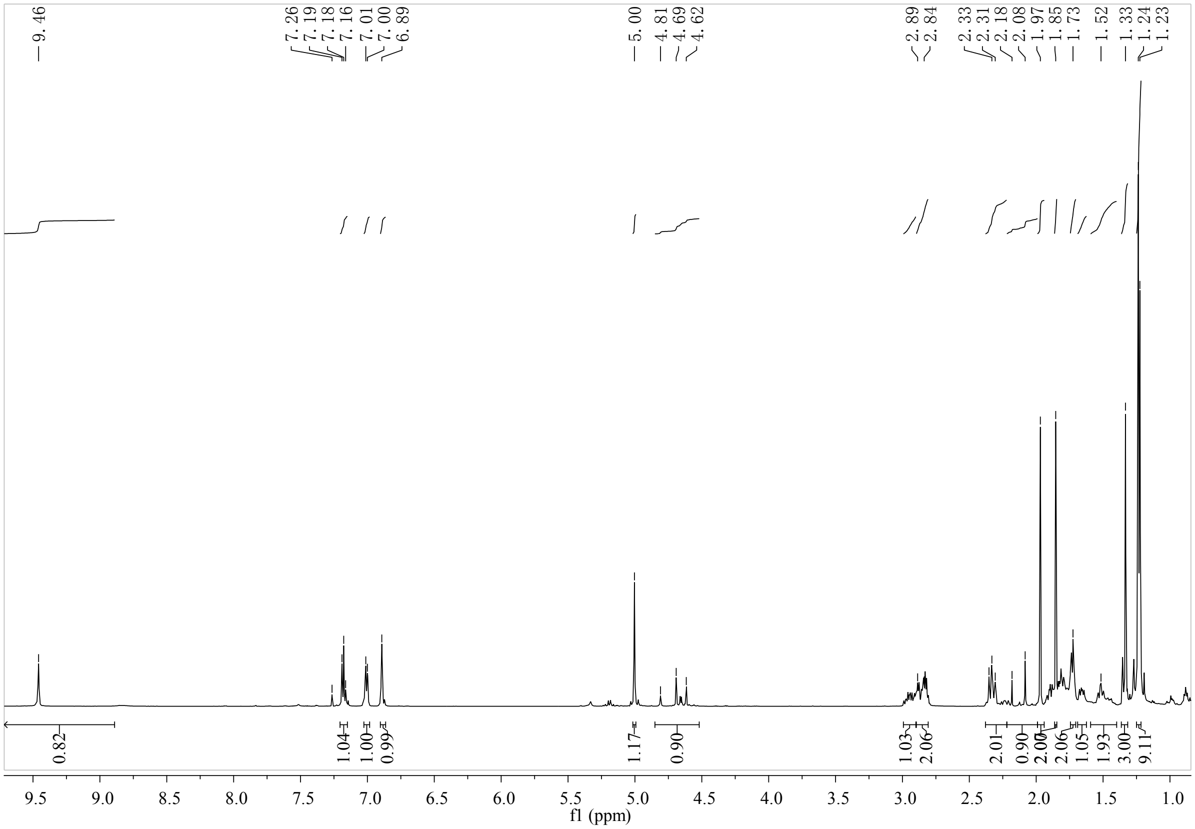

Figure S5. ^{1}H-NMR spectrum of dehydroabietic acid-based hydrazide (**3**) in $CDCl_3$

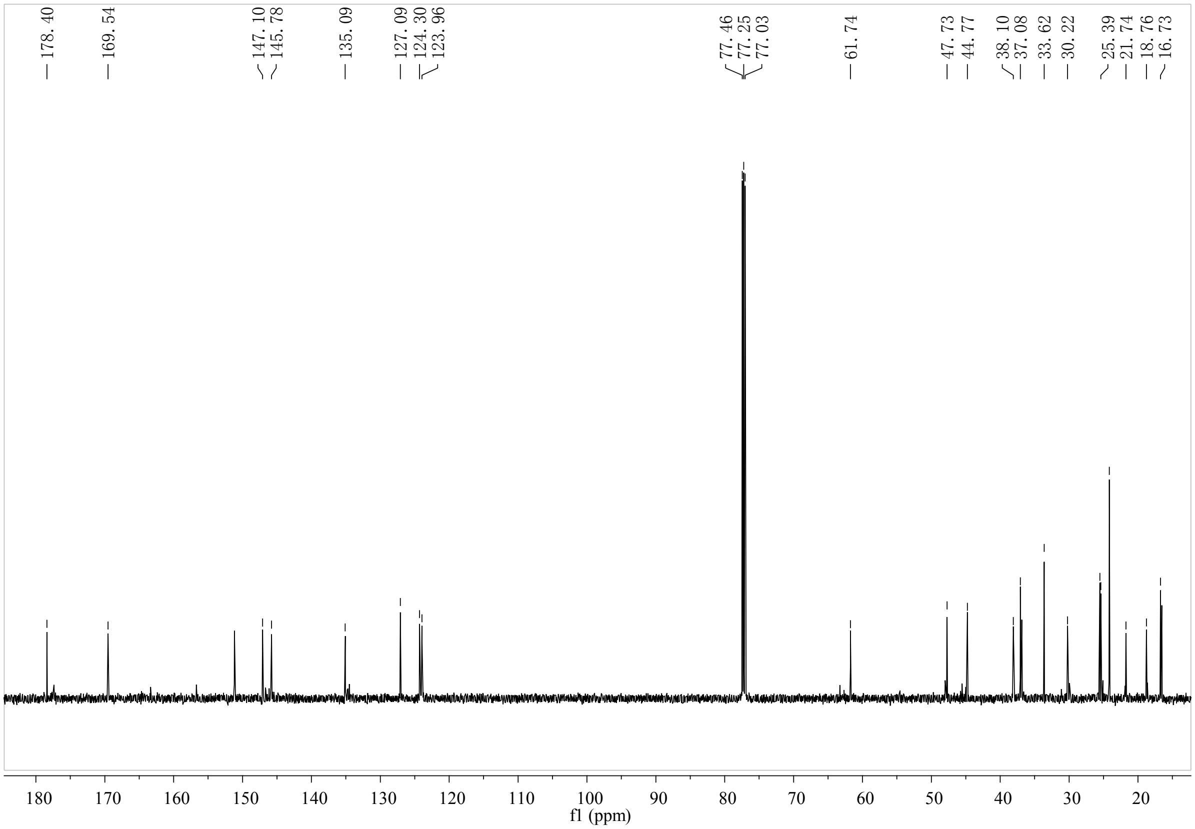

Figure S6. ^{13}C-NMR spectrum of dehydroabietic acid-based hydrazide (**3**) in $CDCl_3$

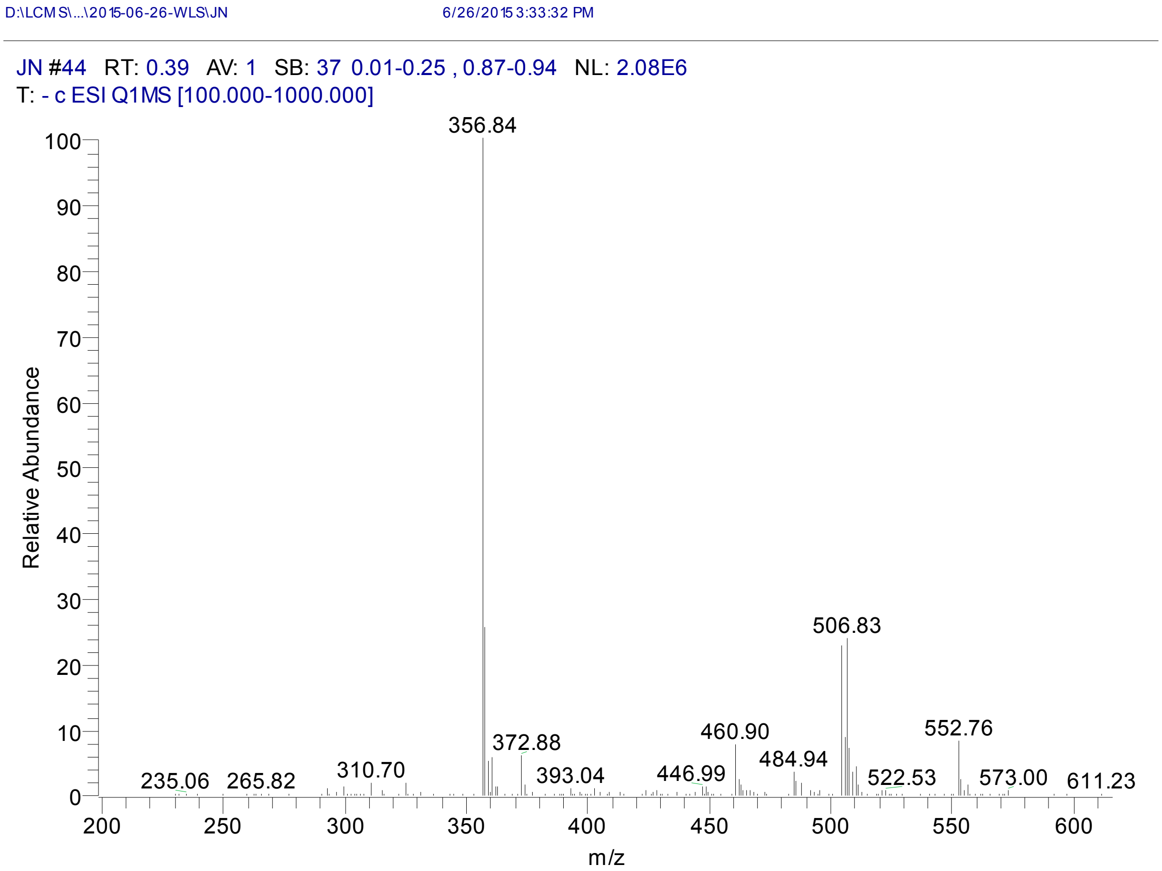

Figure S7. ESI-MS spectrum of dehydroabietic acid-based hydrazide (**3**)

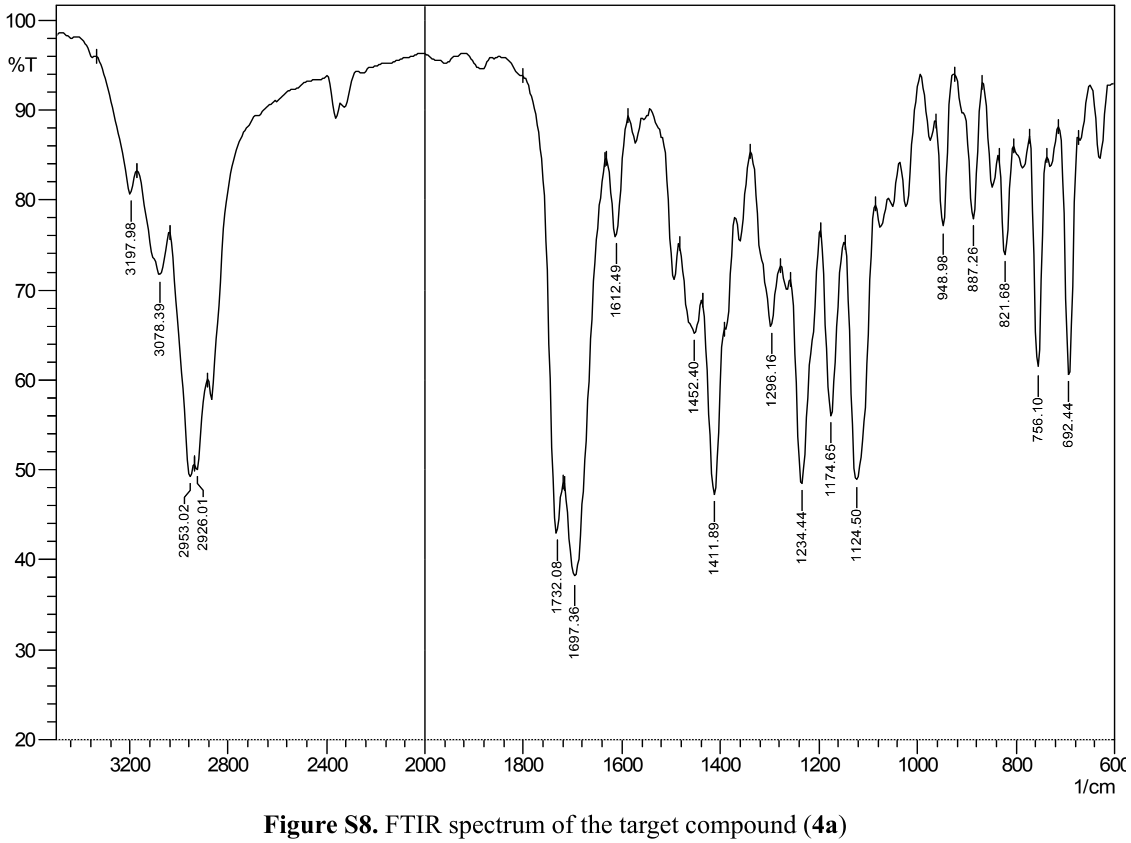

Figure S8. FTIR spectrum of the target compound (**4a**)

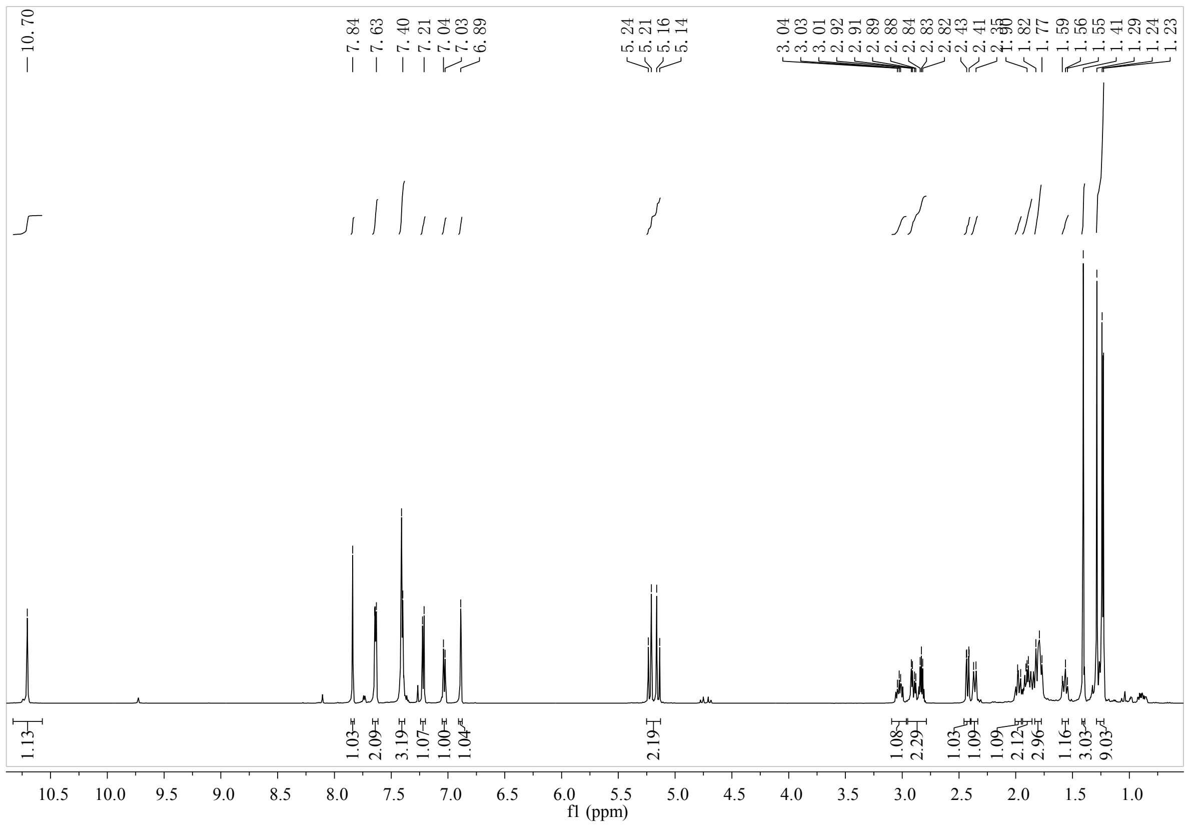

Figure S9. ^{1}H-NMR spectrum of the target compound (**4a**) in $CDCl_3$

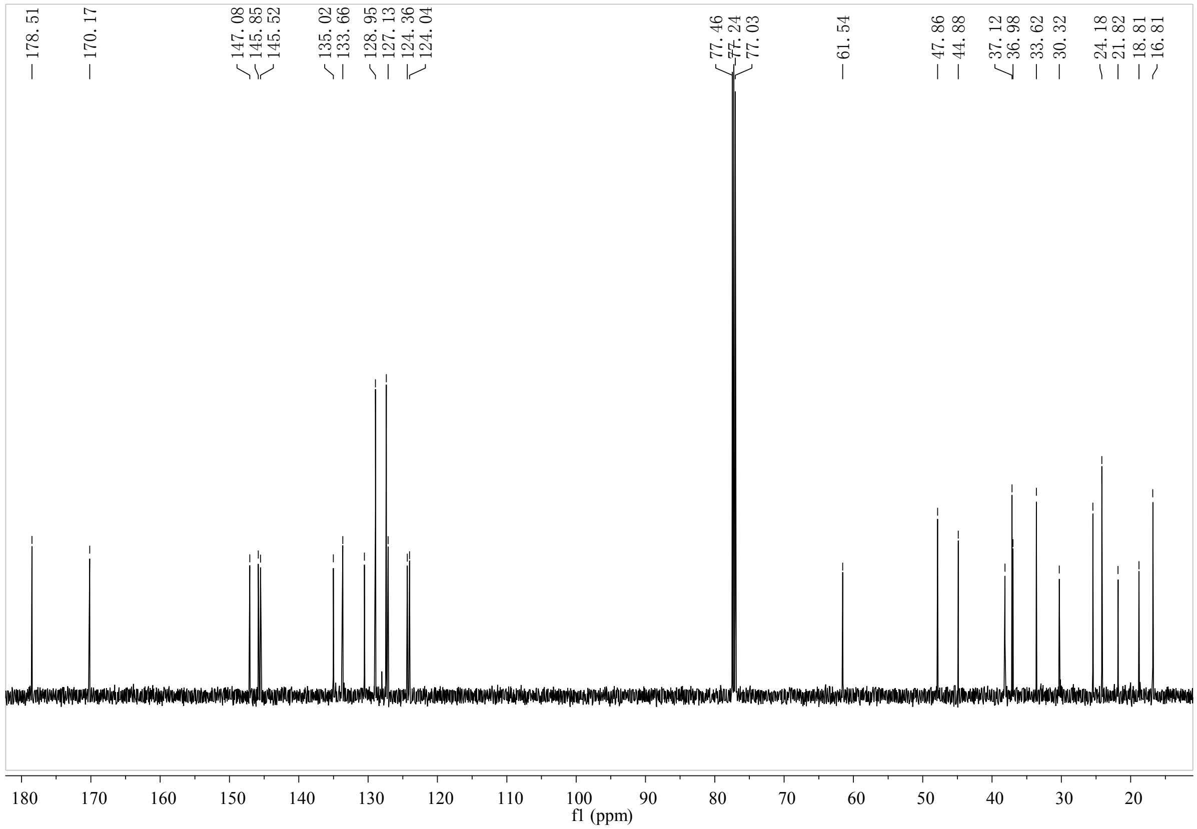

Figure S10. ^{13}C-NMR spectrum of the target compound (**4a**) in $CDCl_3$

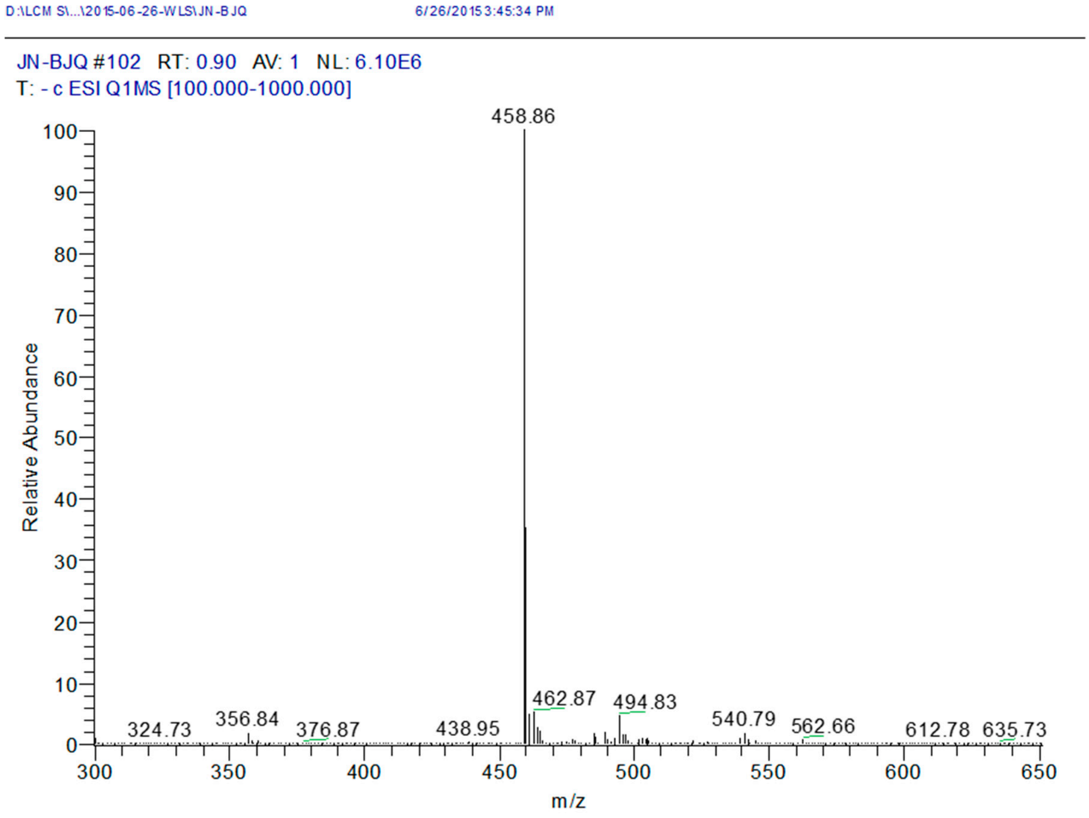

Figure S11. ESI-MS spectrum of the target compound (**4a**)

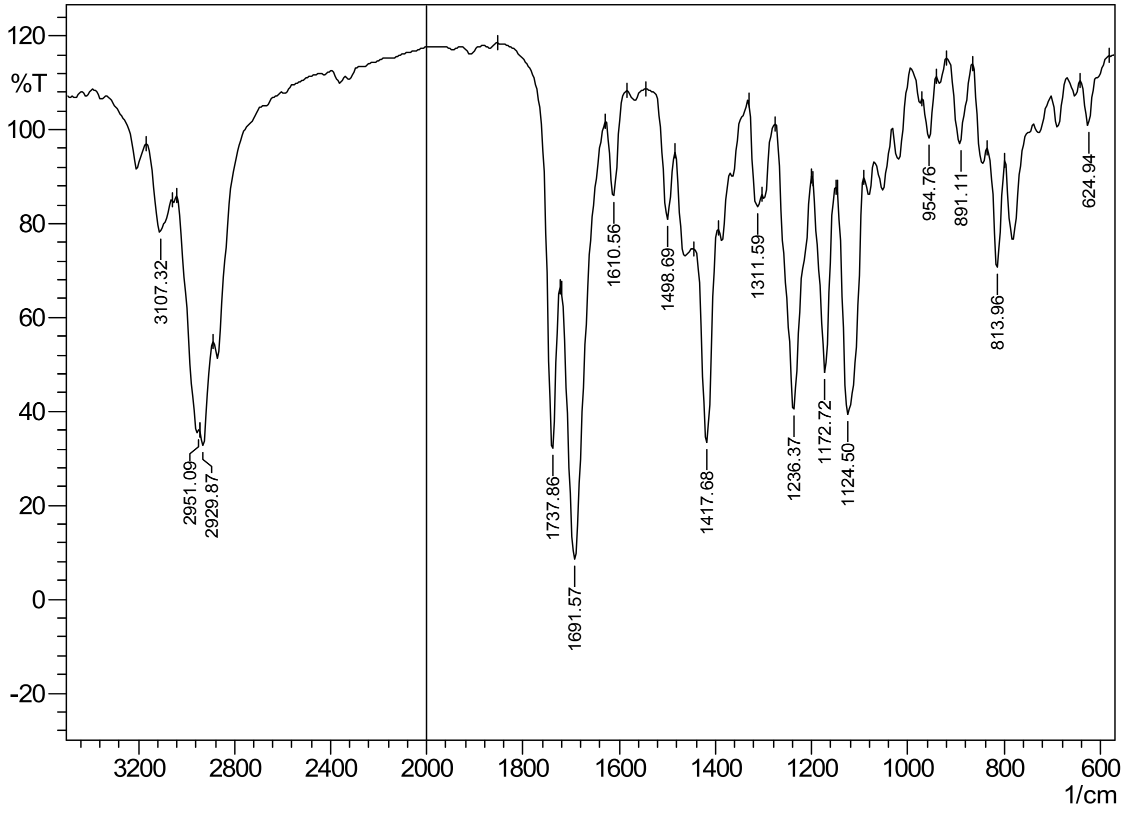

Figure S12. FTIR spectrum of the target compound (**4b**)

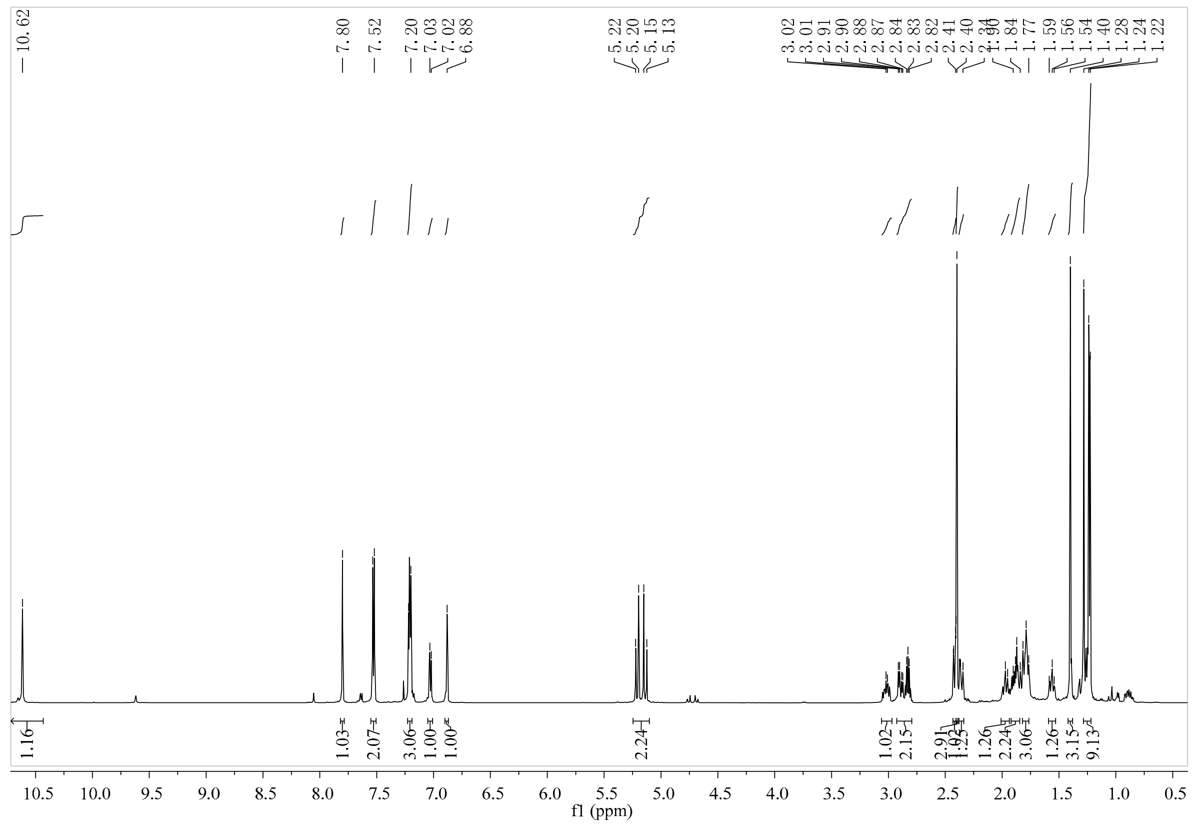

Figure S13. ^{1}H-NMR spectrum of the target compound (**4b**) in $CDCl_3$

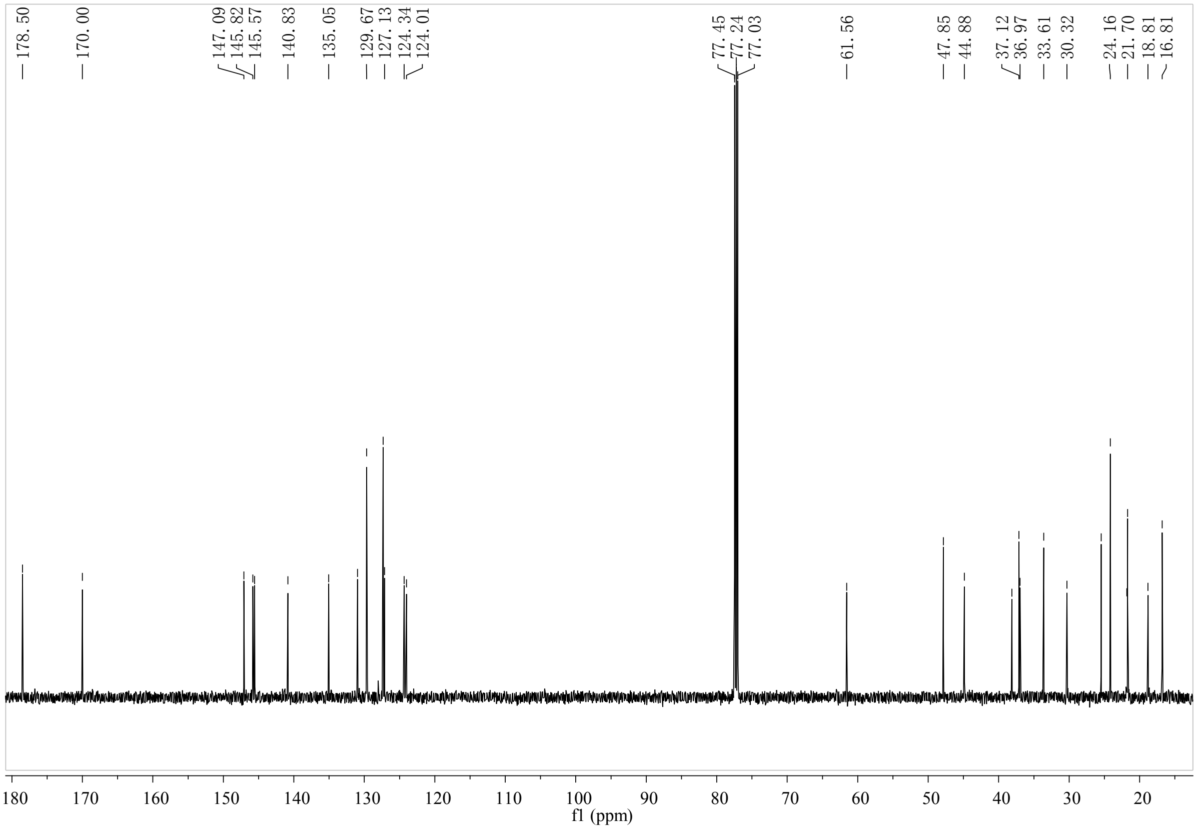

Figure S14. ^{13}C-NMR spectrum of the target compound (**4b**) in $CDCl_3$

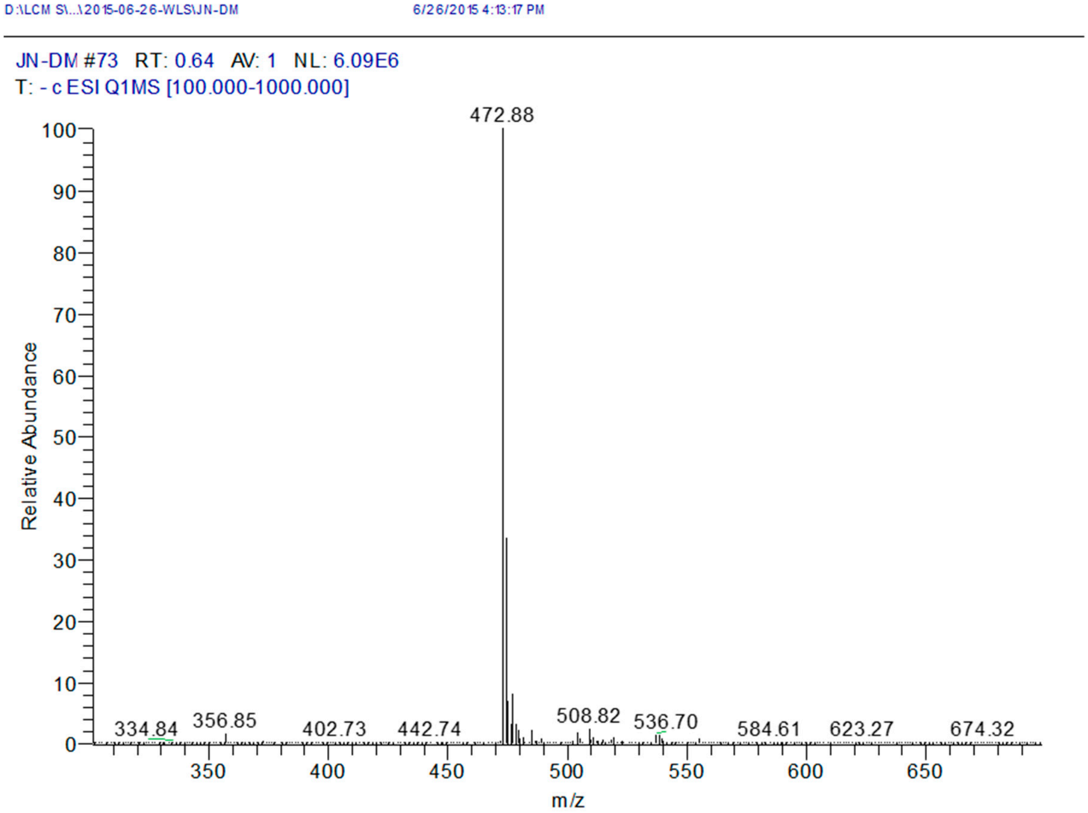

Figure S15. ESI-MS spectrum of the target compound (**4b**)

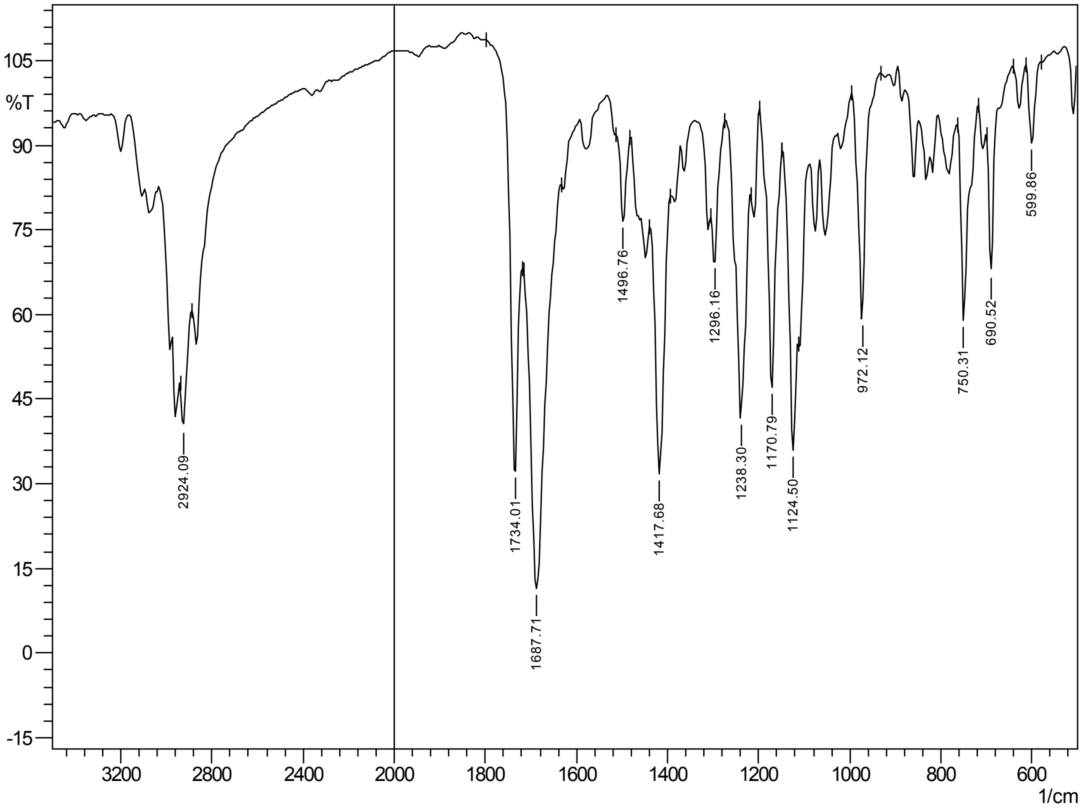

Figure S16. FTIR spectrum of the target compound (**4c**)

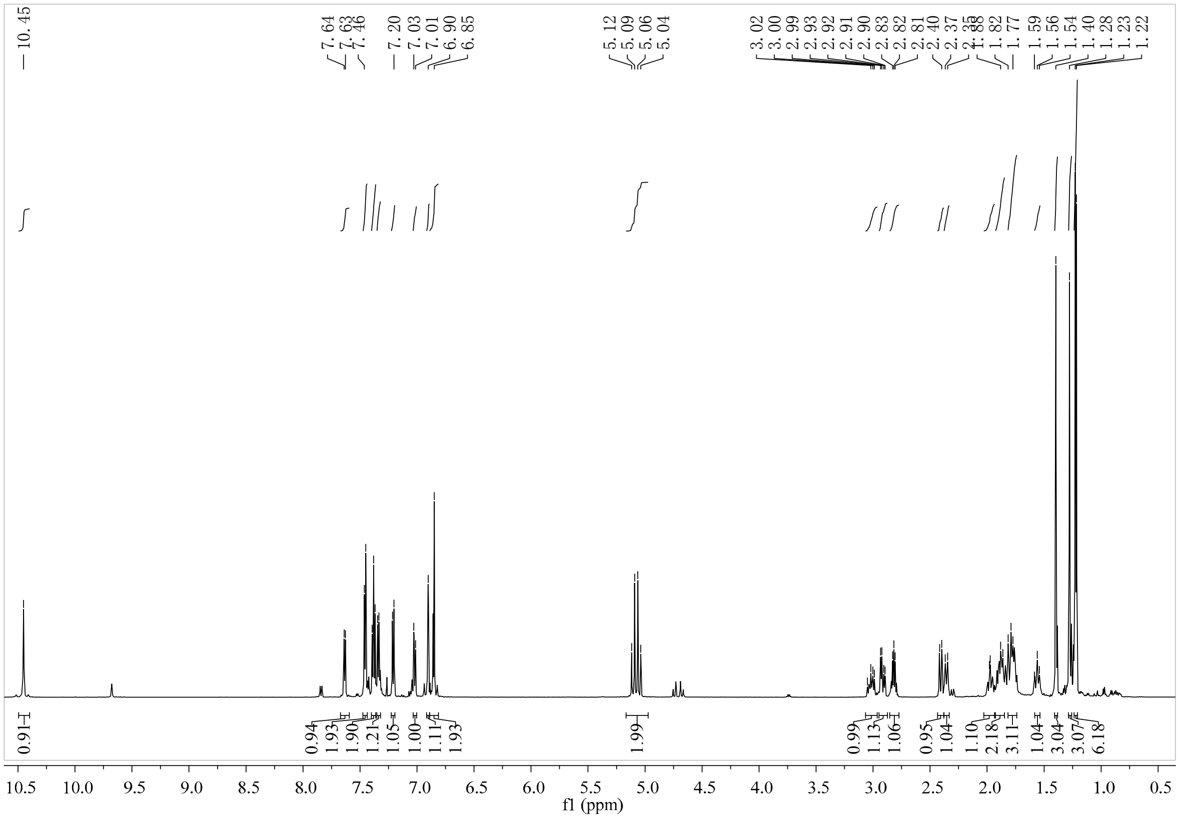

Figure S17. ^{1}H-NMR spectrum of the target compound (**4c**) in $CDCl_3$

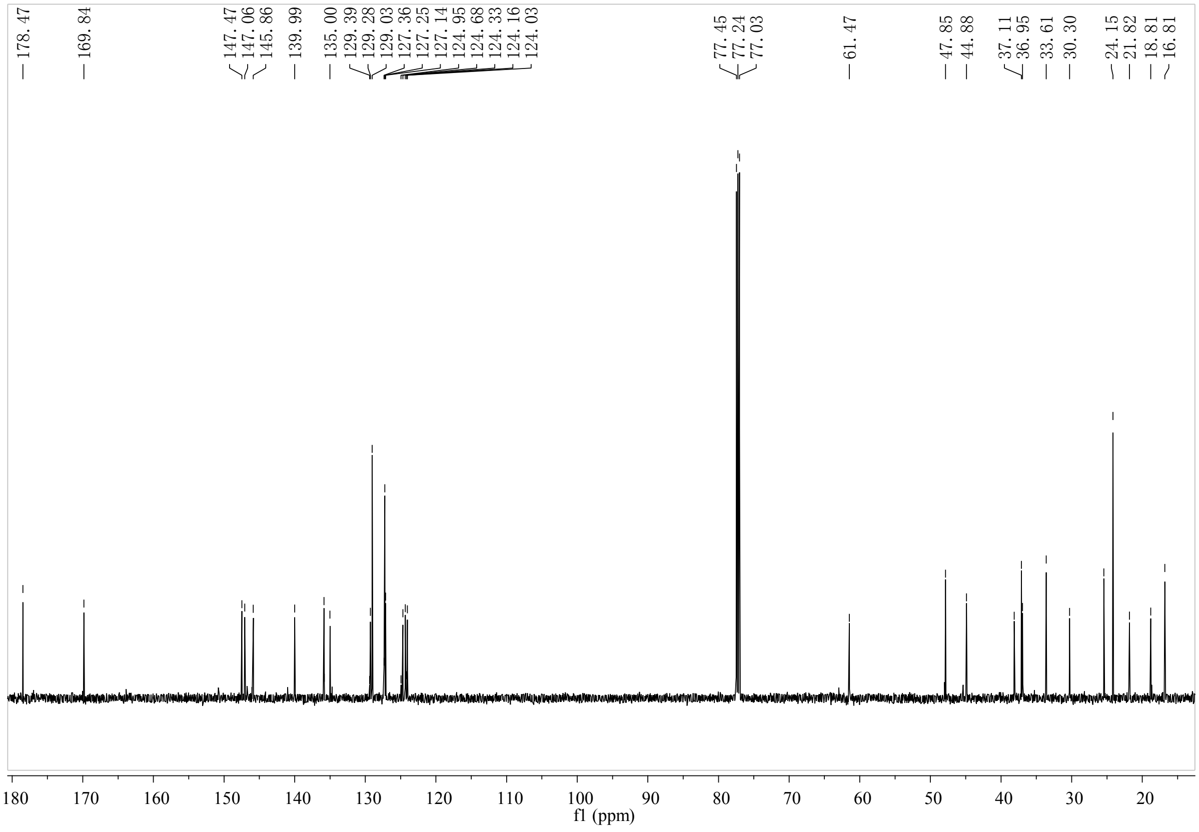

Figure S18. ^{13}C-NMR spectrum of the target compound (**4c**) in $CDCl_3$

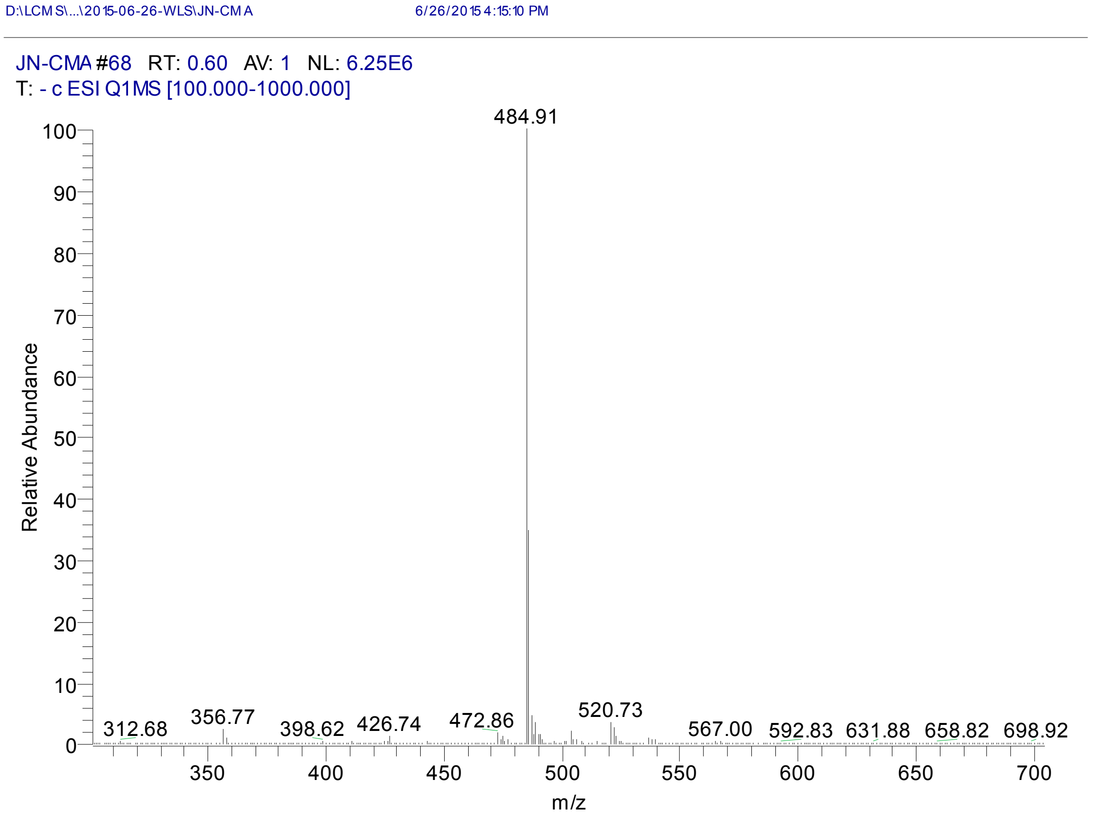

Figure S19. ESI-MS spectrum of the target compound (**4c**)

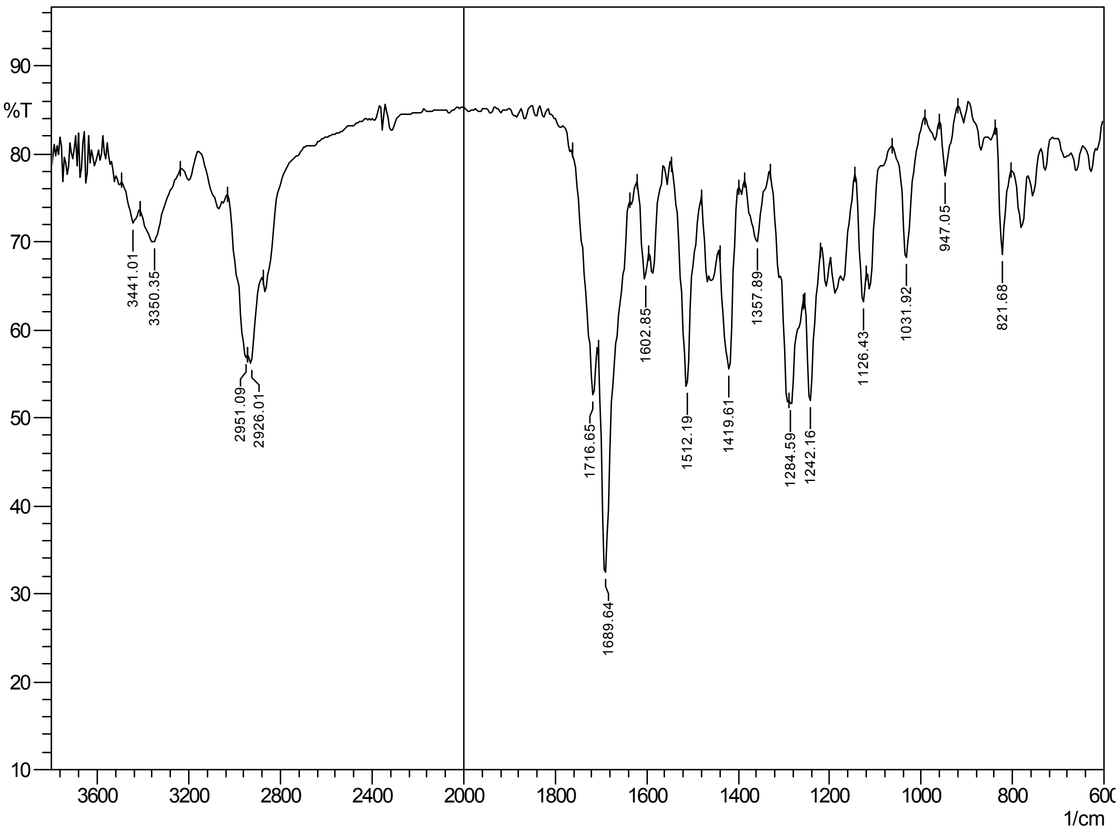

Figure S20. FTIR spectrum of the target compound (**4d**)

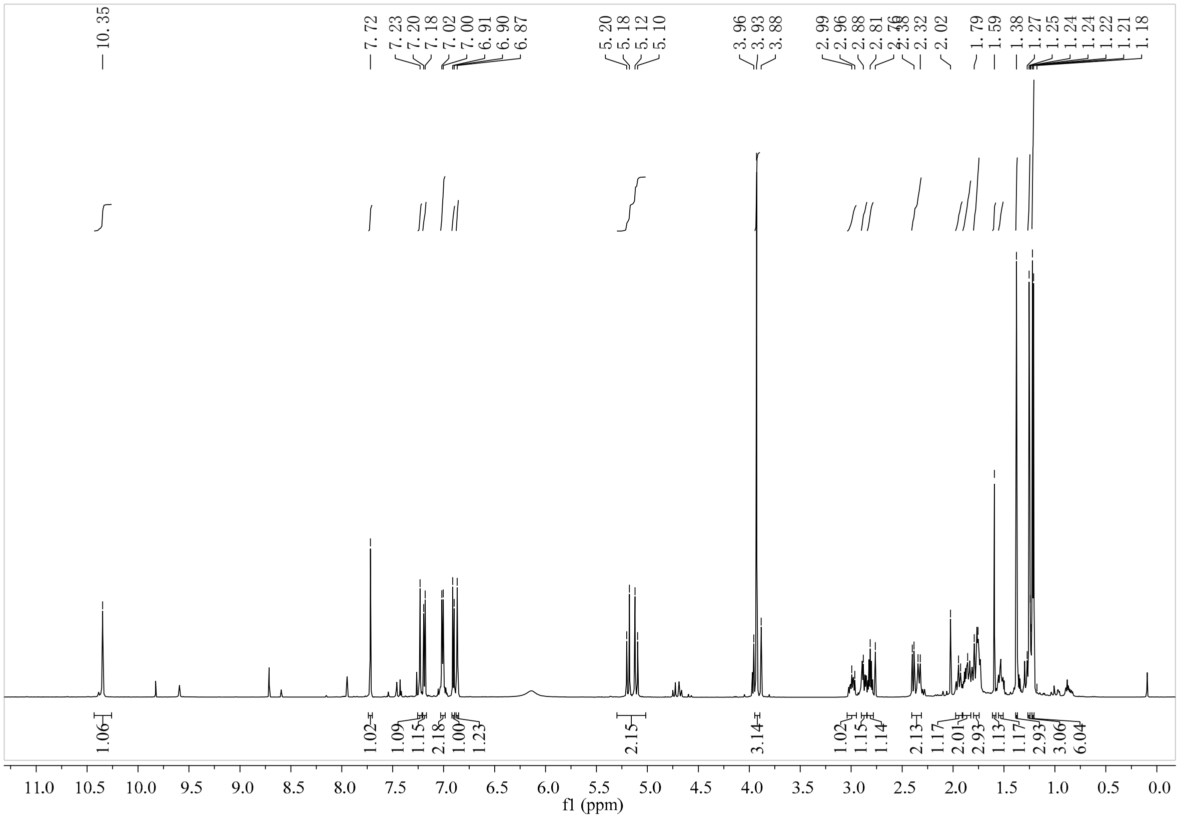

Figure S21. ^{1}H-NMR spectrum of the target compound (**4d**) in $CDCl_3$

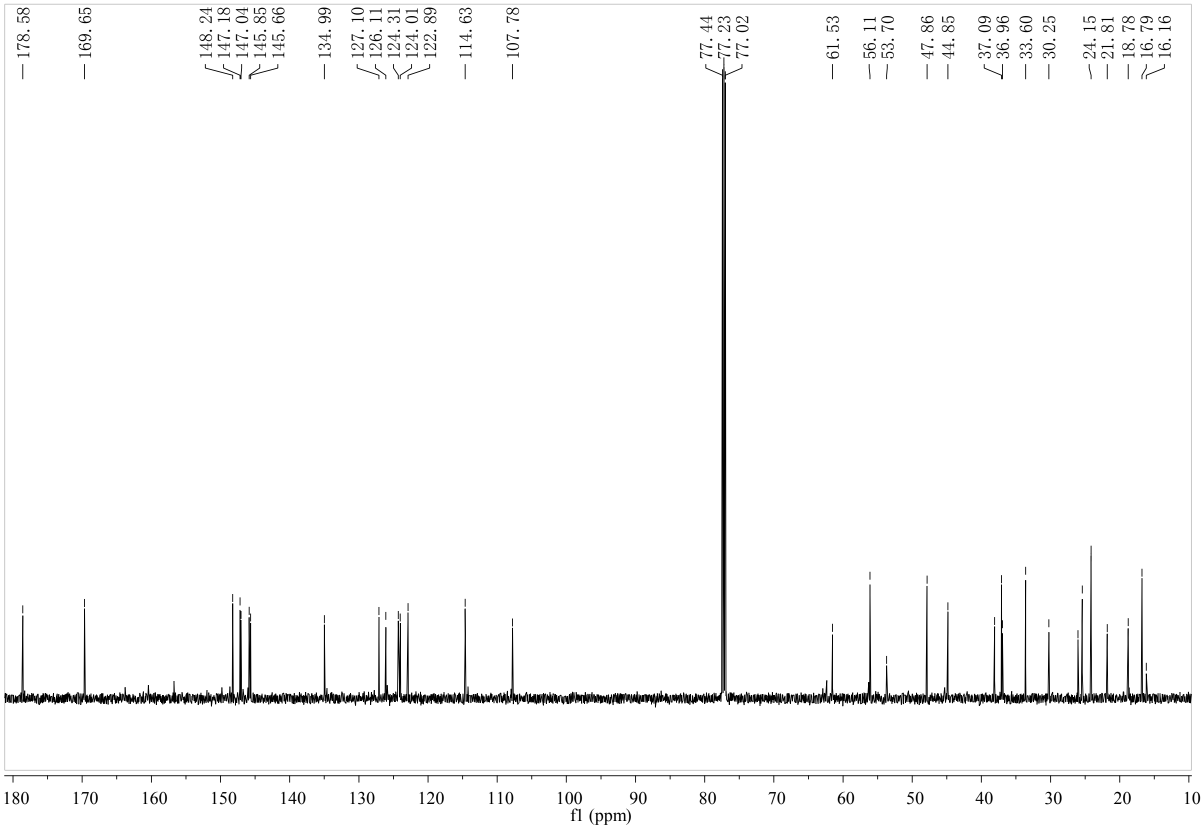

Figure S22. ^{13}C-NMR spectrum of the target compound (**4d**) in $CDCl_3$

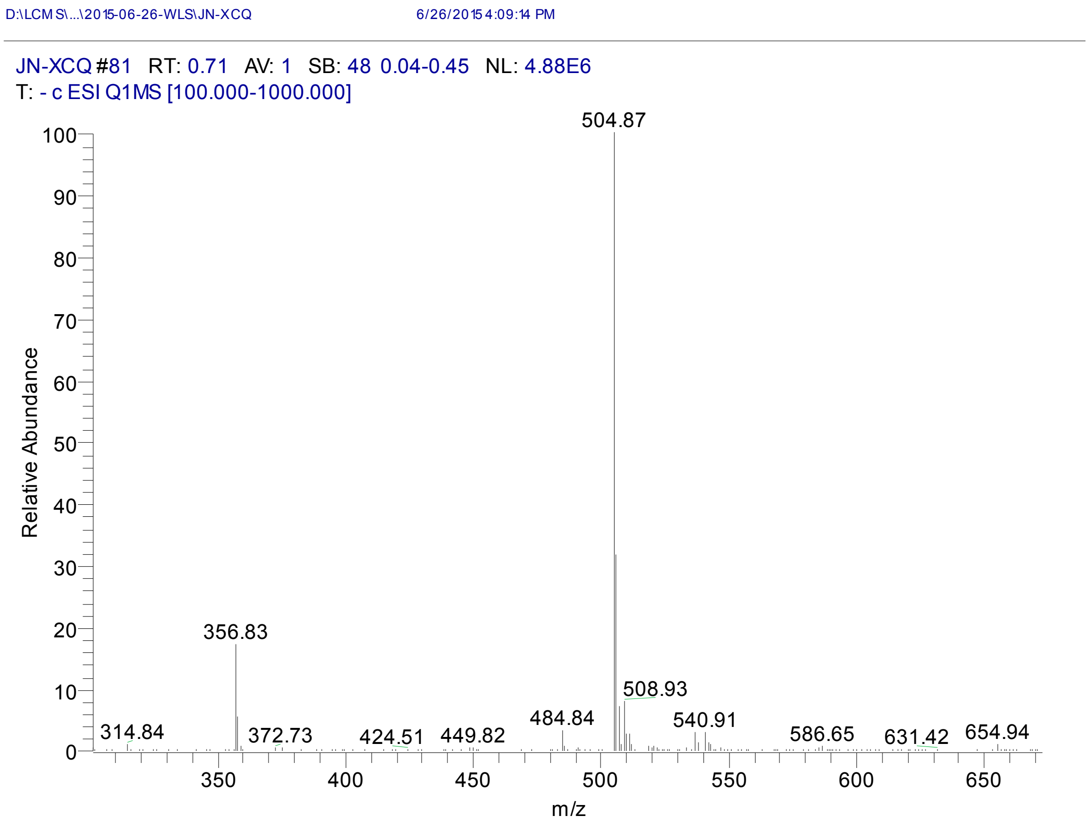

Figure S23. ESI-MS spectrum of the target compound (**4d**)

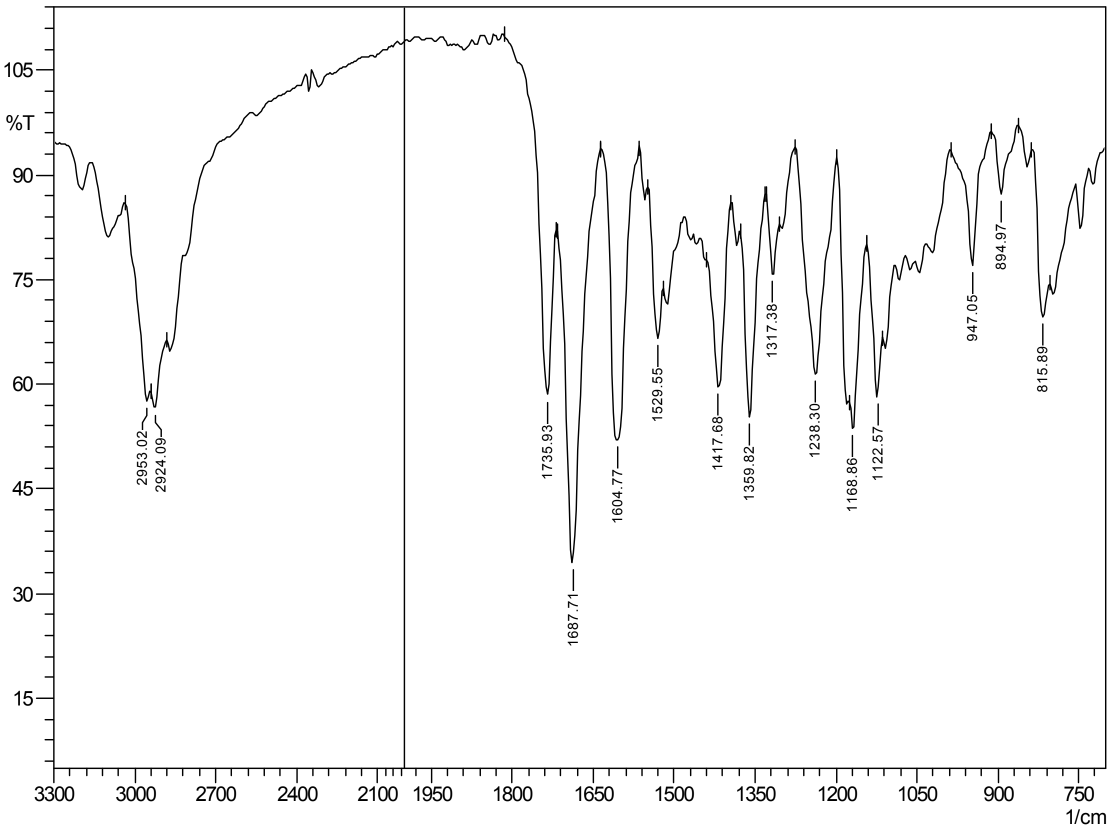

Figure S24. FTIR spectrum of the target compound (**4e**)

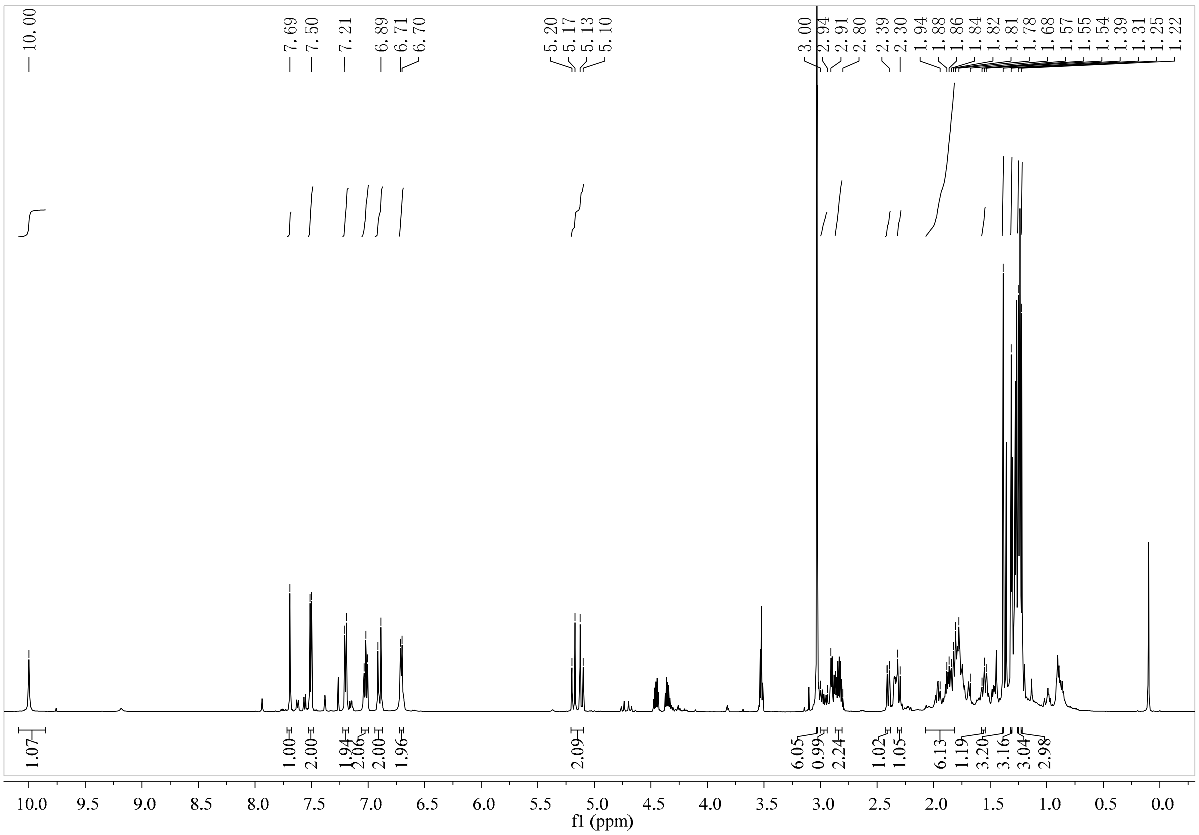

Figure S25. ^{1}H-NMR spectrum of the target compound (**4e**) in $CDCl_3$

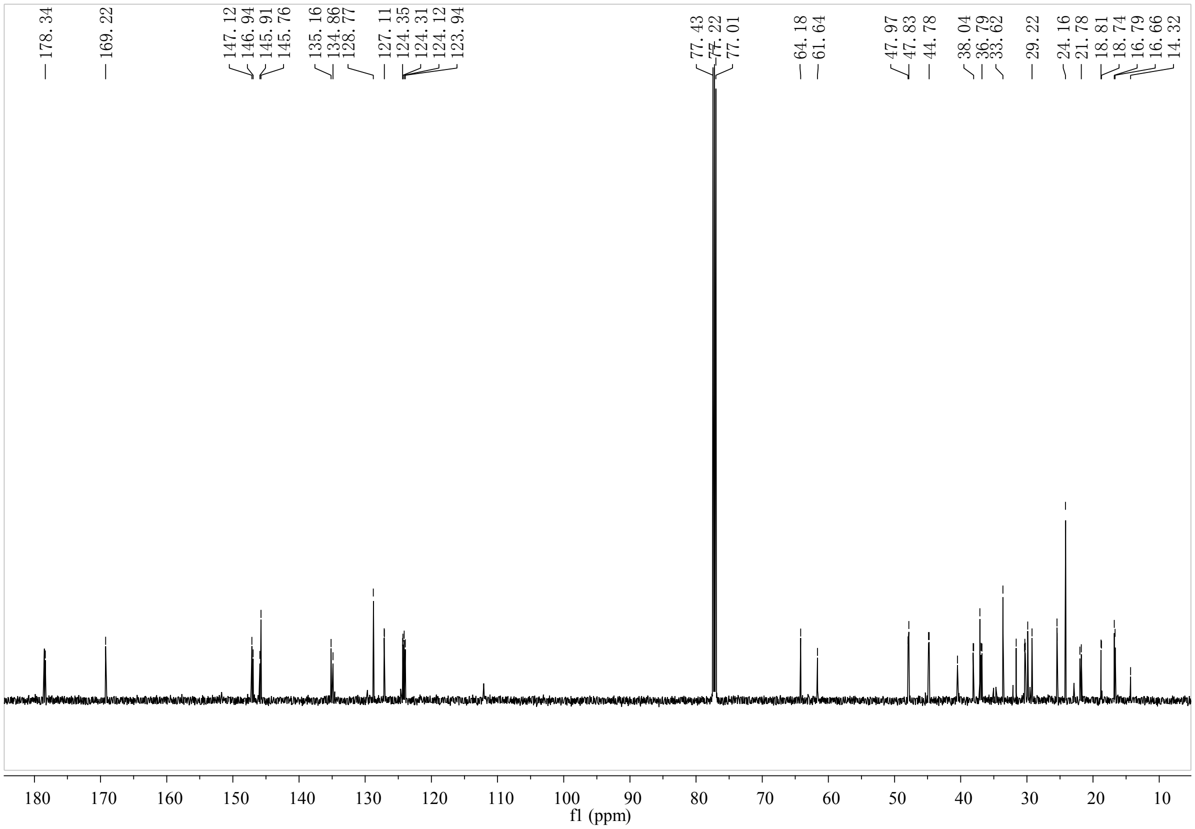

Figure S26. ^{13}C-NMR spectrum of the target compound (**4e**) in $CDCl_3$

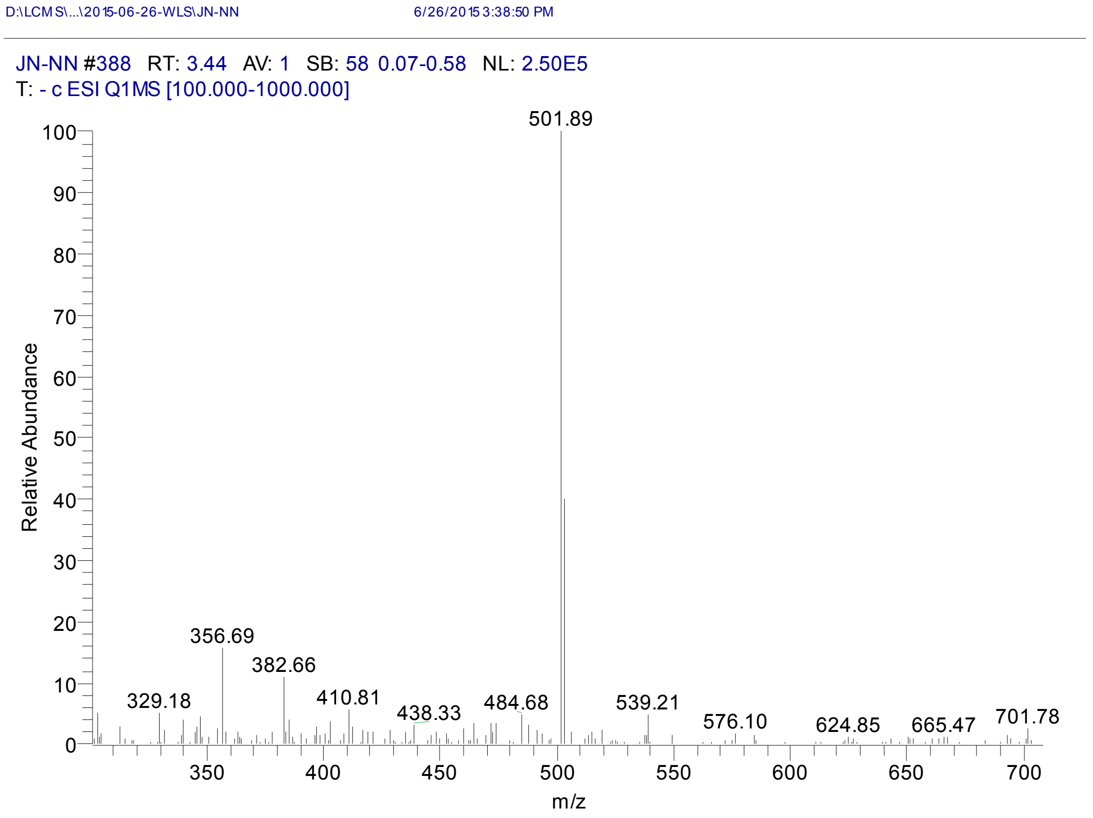

Figure S27. ESI-MS spectrum of the target compound (**4e**)

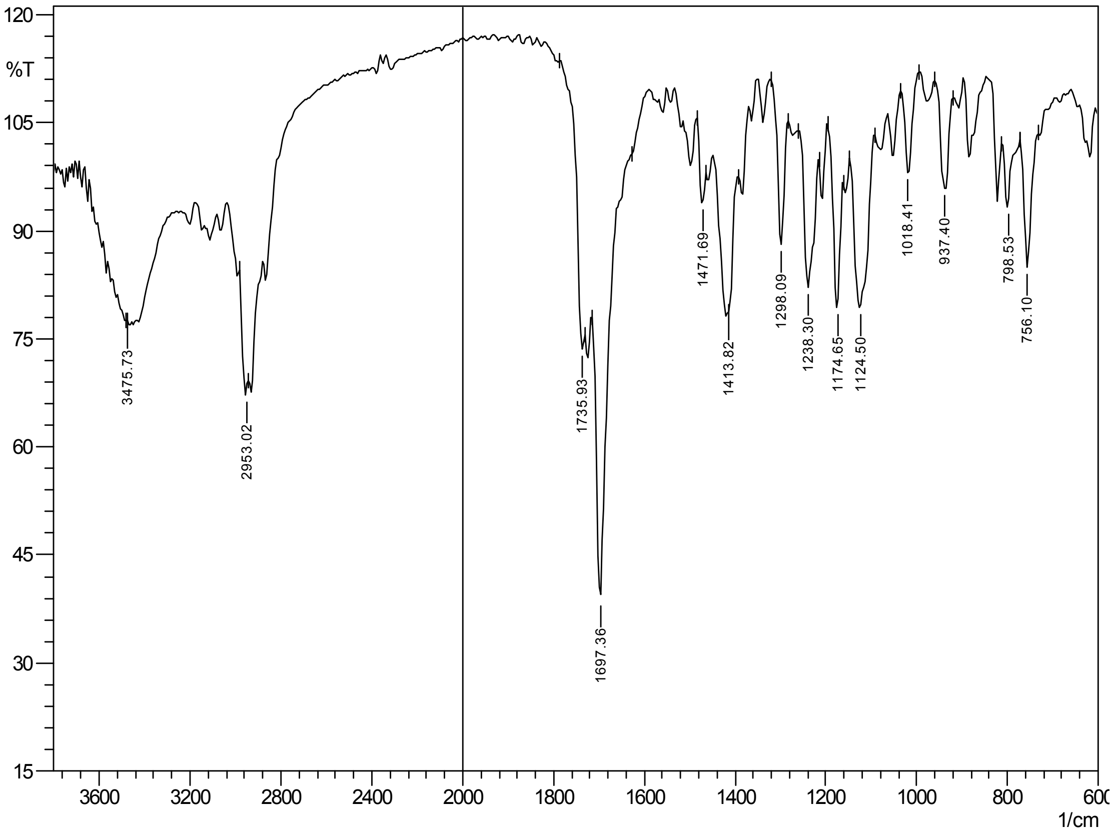

Figure S28. FTIR spectrum of the target compound (**4f**)

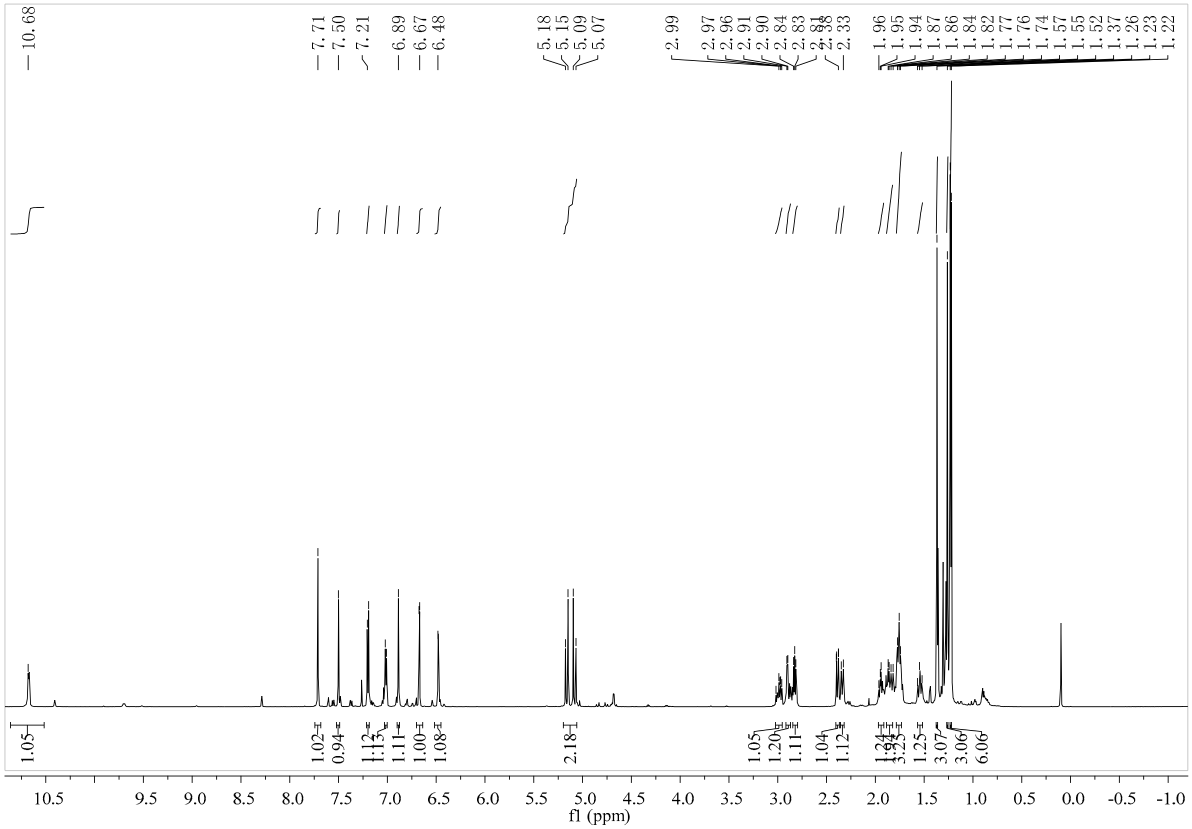

Figure S29. ^{1}H-NMR spectrum of the target compound (**4f**) in $CDCl_3$

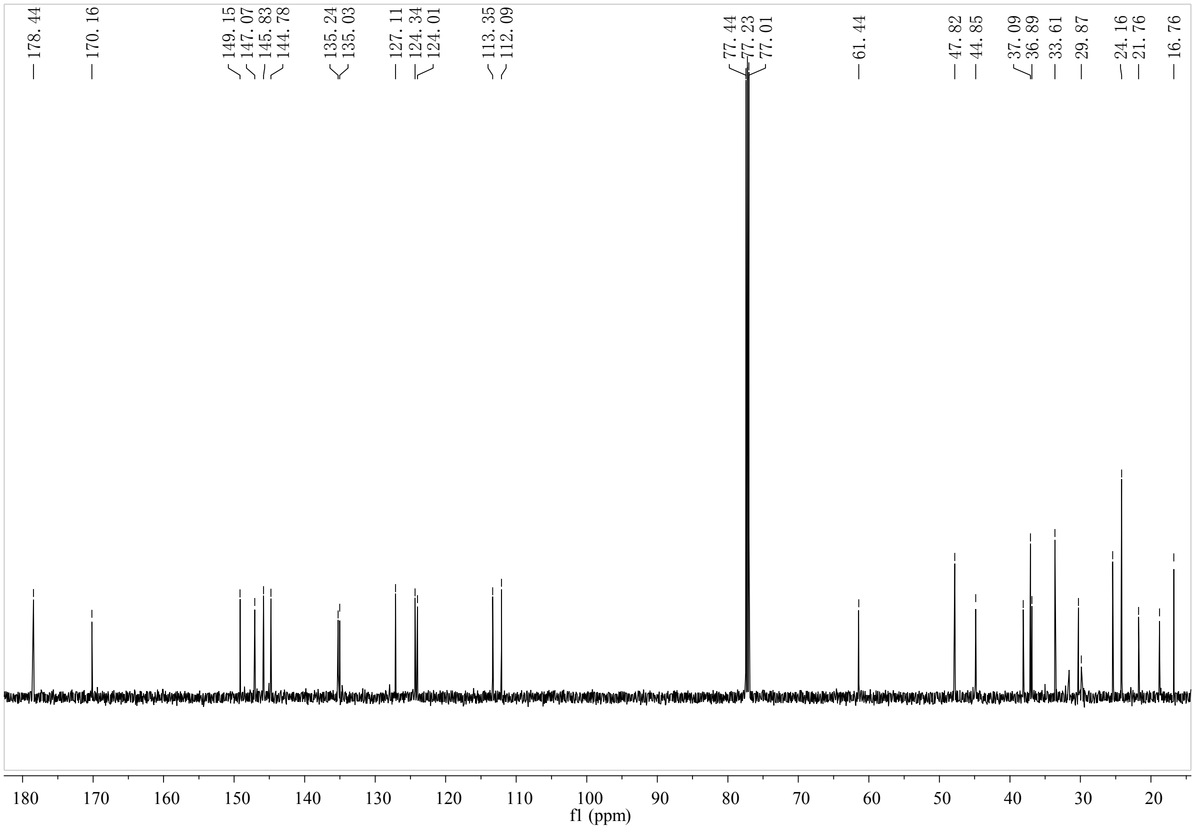

Figure S30. ^{13}C-NMR spectrum of the target compound (**4f**) in $CDCl_3$

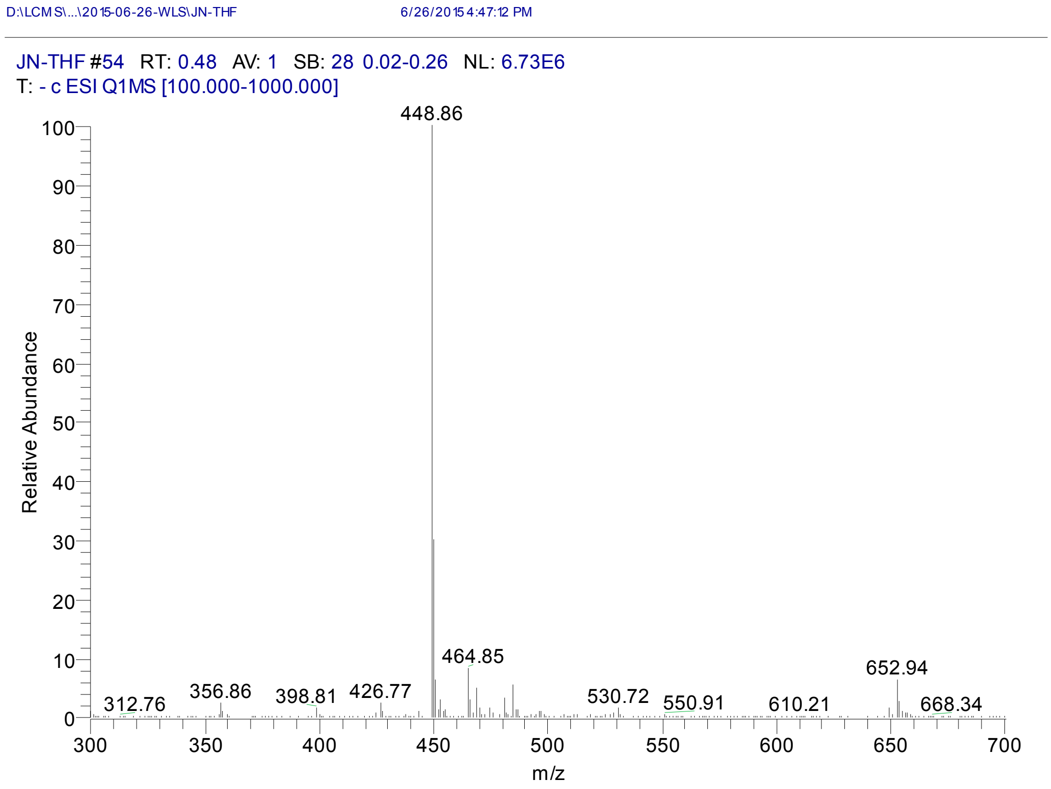

Figure S31. ESI-MS spectrum of the target compound (**4f**)

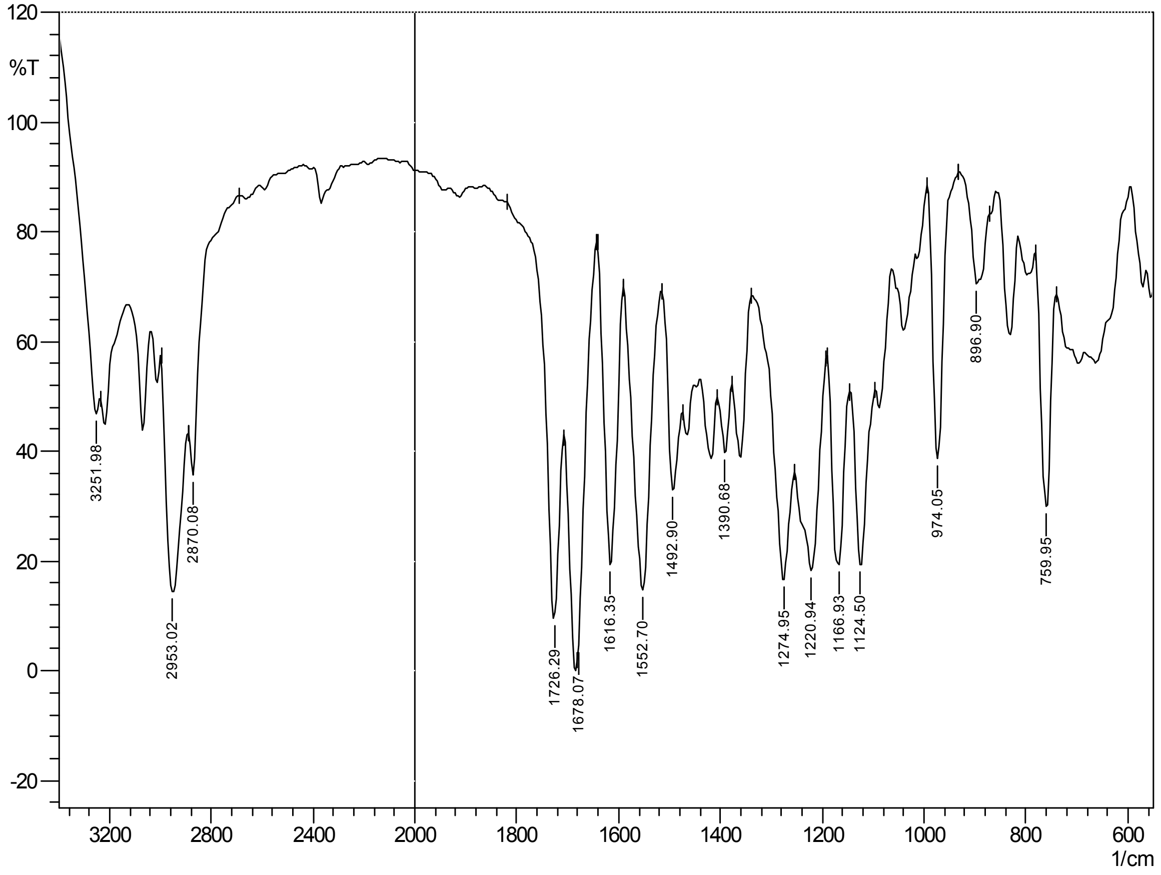

Figure S32. FTIR spectrum of the target compound (**4g**)

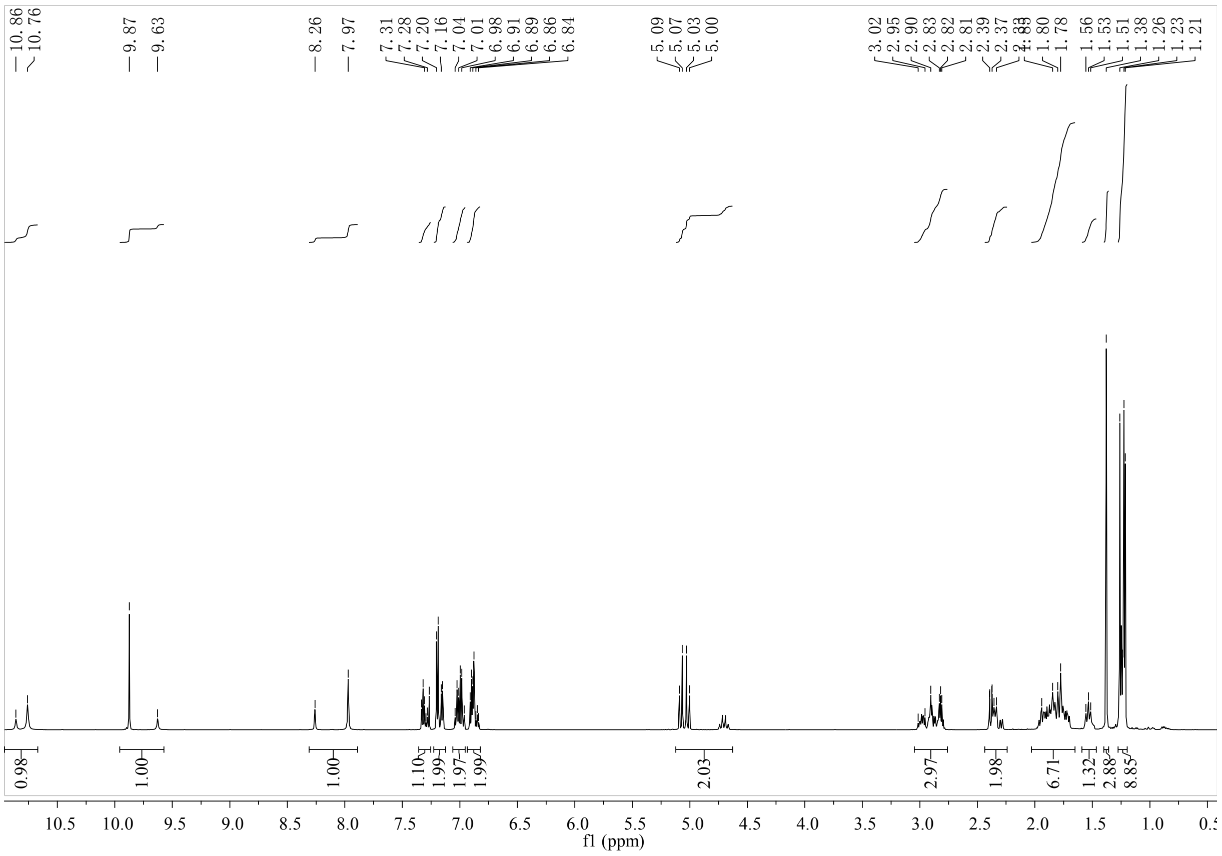

Figure S33. ^{1}H-NMR spectrum of the target compound (**4g**) in $CDCl_3$

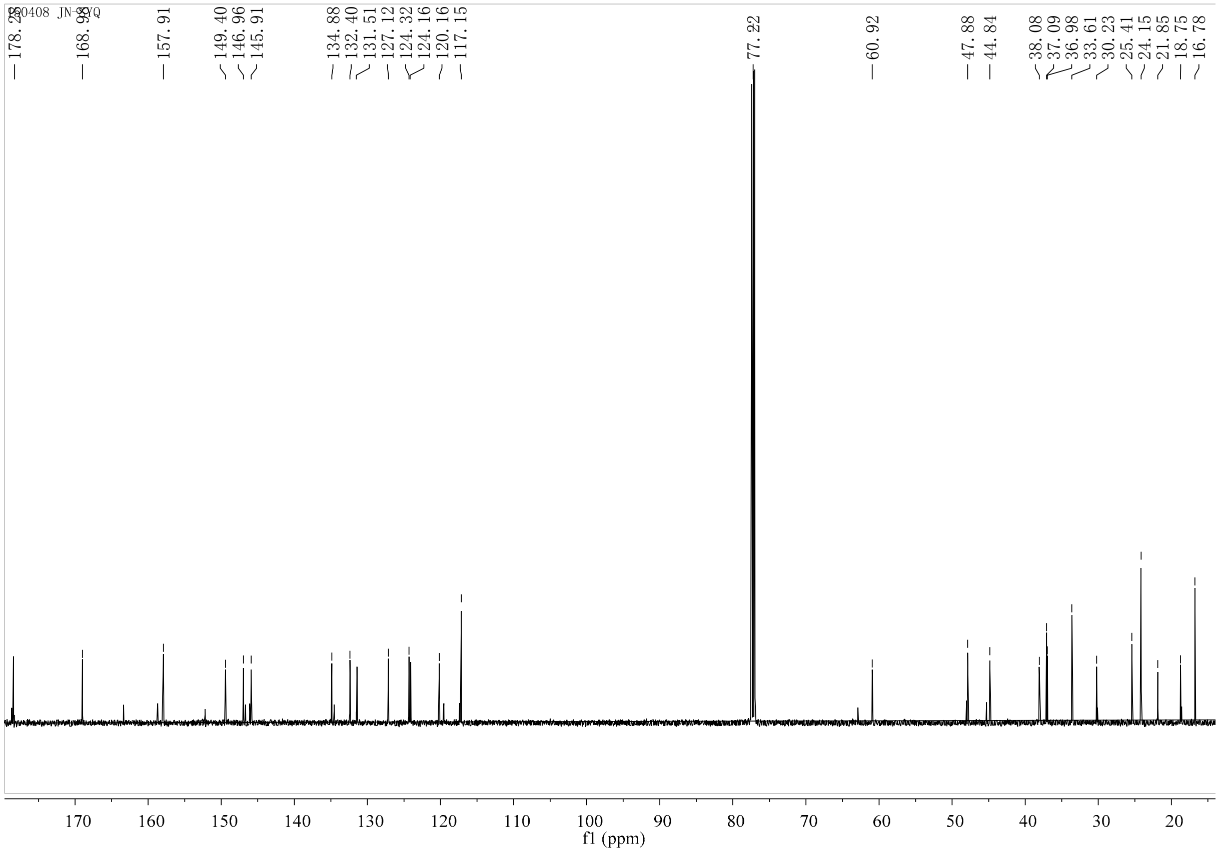

Figure S34. ^{13}C-NMR spectrum of the target compound (**4g**) in $CDCl_3$

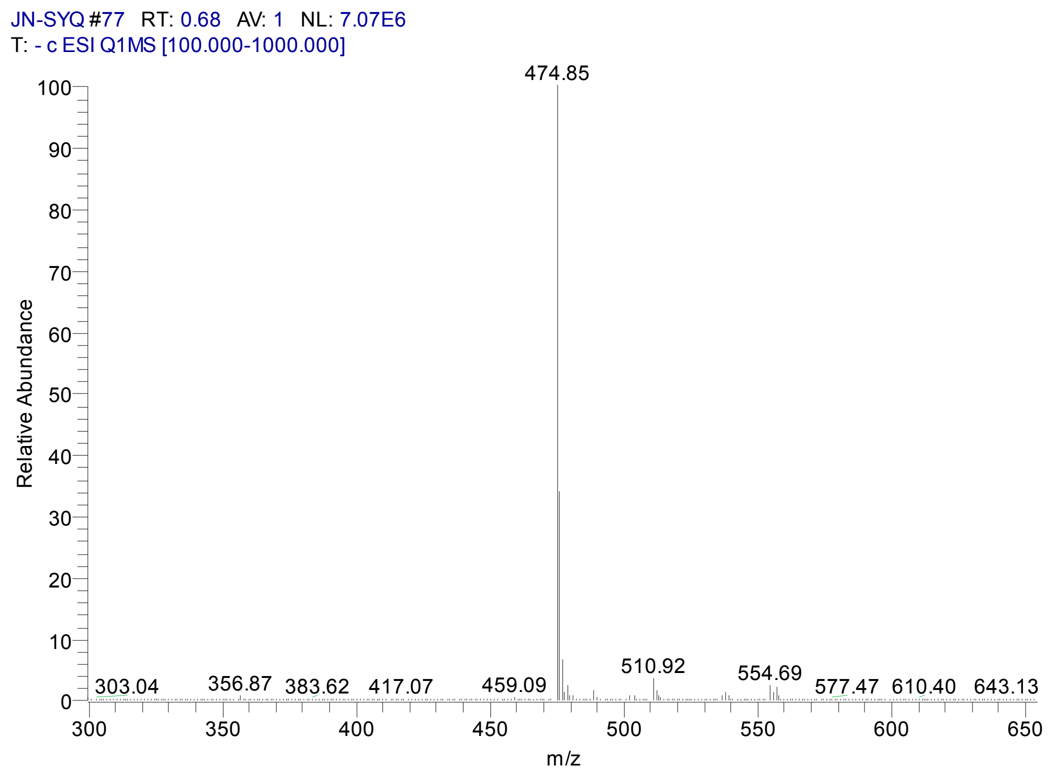

Figure S35. ESI-MS spectrum of the target compound (**4g**)

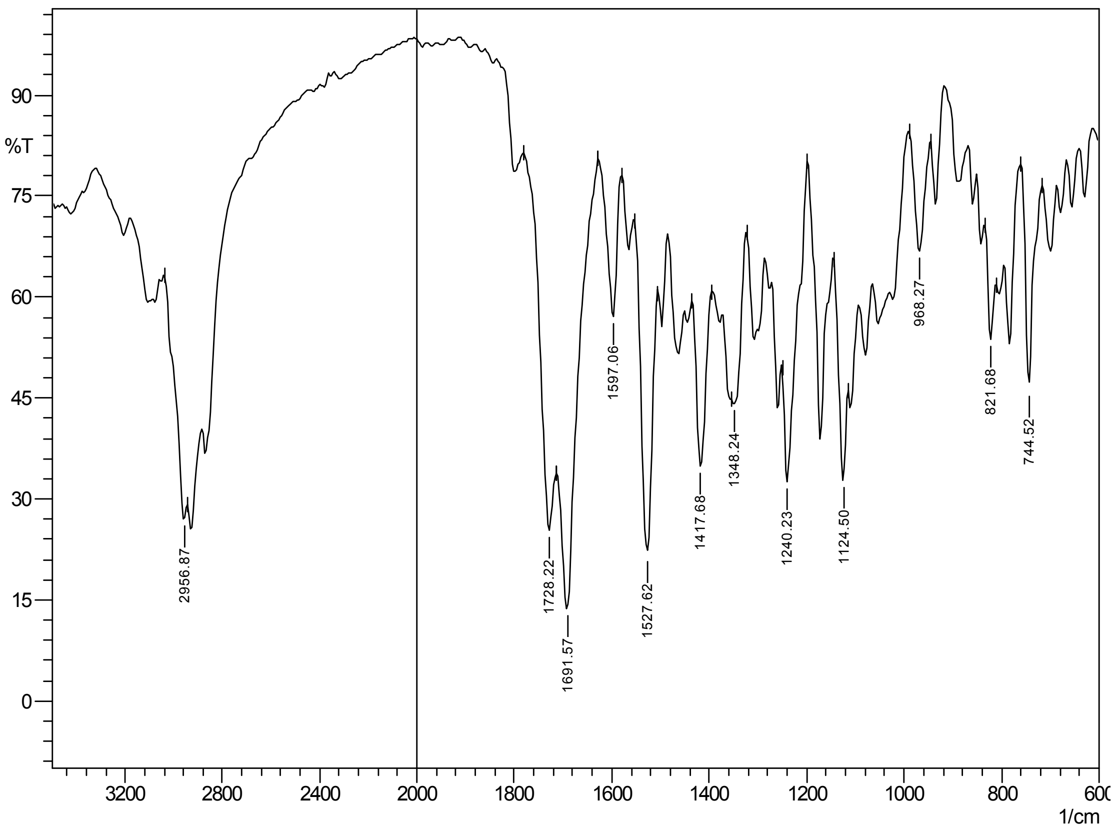

Figure S36. FTIR spectrum of the target compound (**4h**)

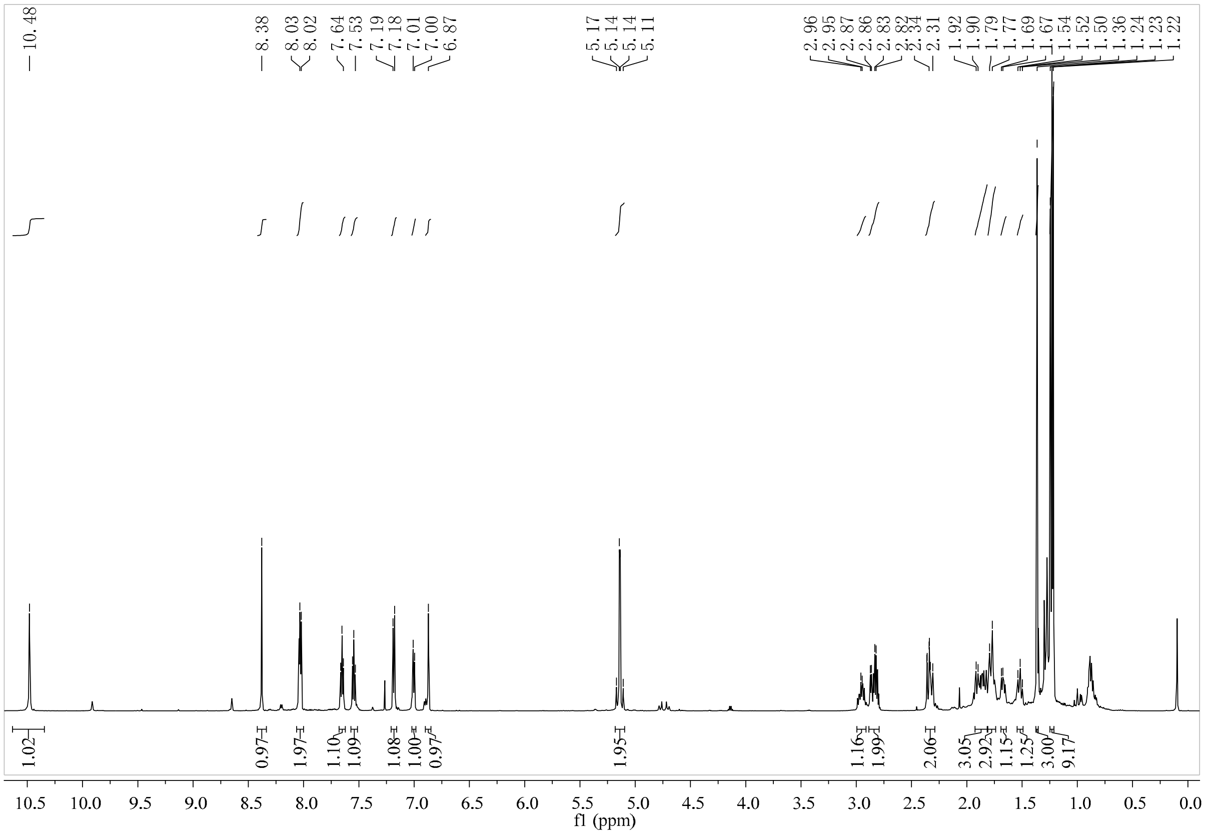

Figure S37. ^{1}H-NMR spectrum of the target compound (**4h)** in $CDCl_3$

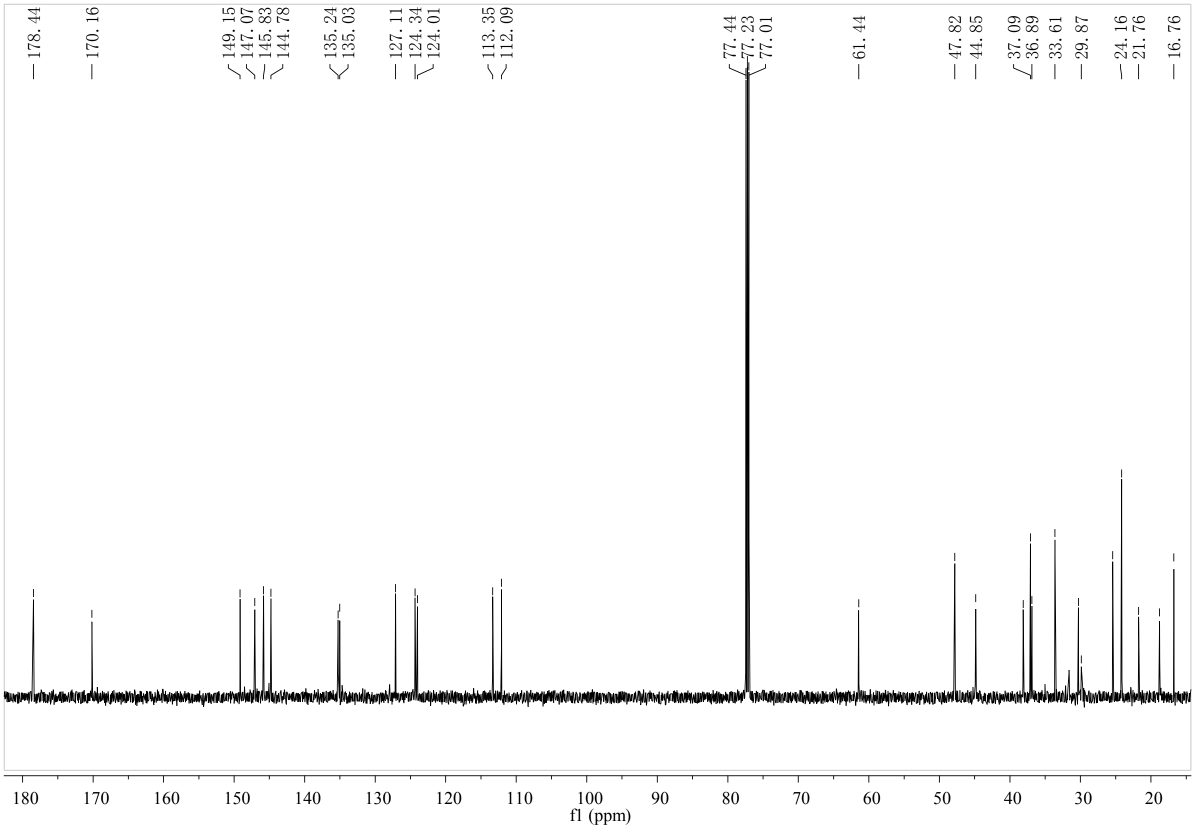

Figure S38. ^{13}C-NMR spectrum of the target compound (**4h)** in $CDCl_3$

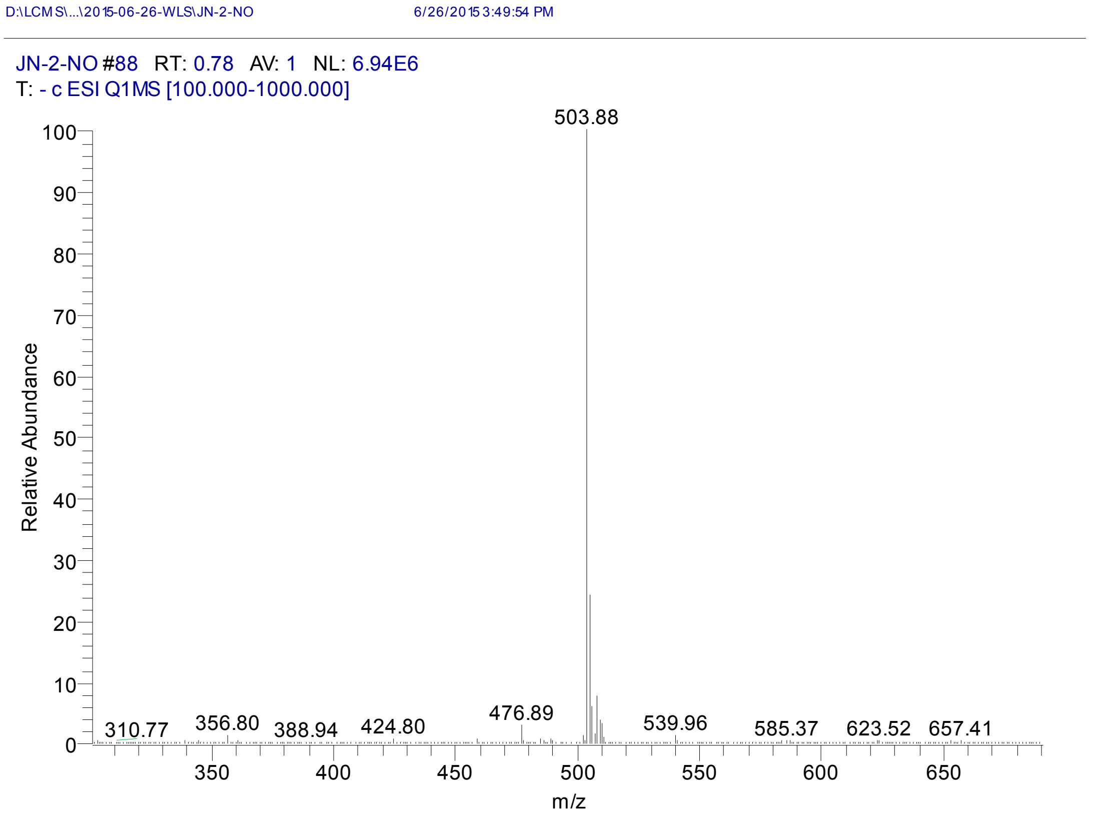

Figure S39. ESI-MS spectrum of the target compound (**4h**)

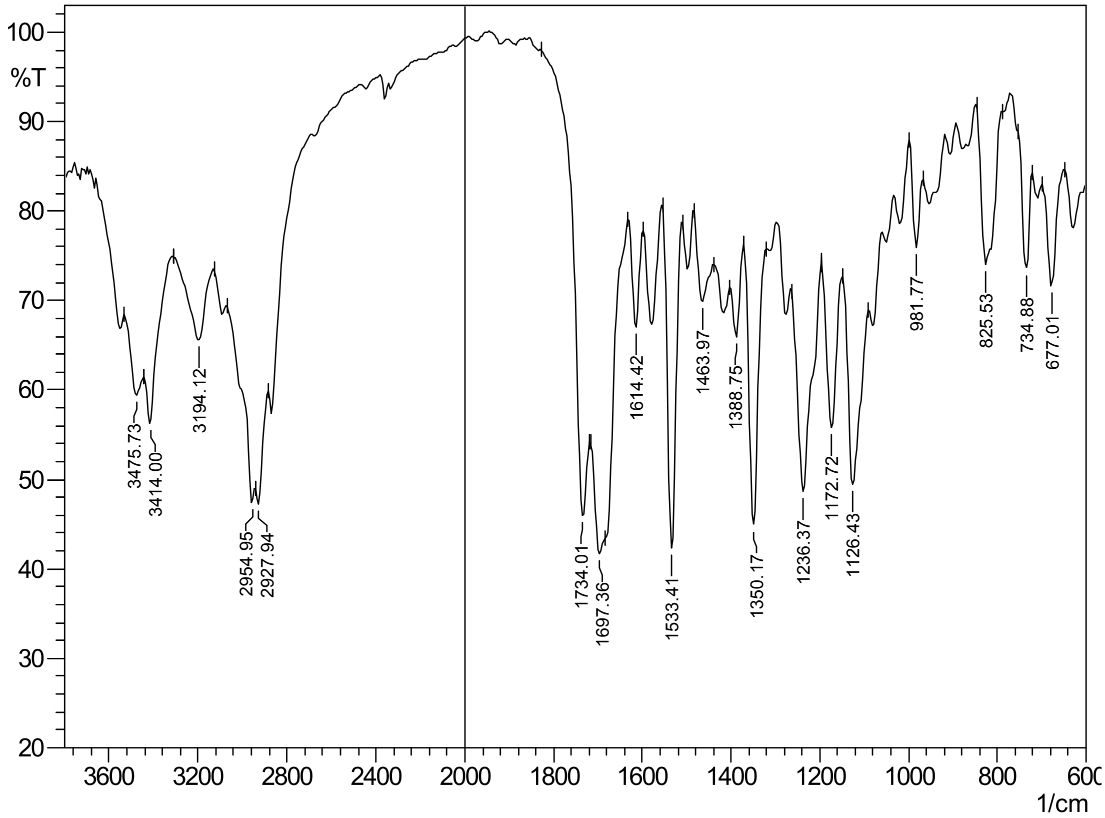

Figure S40. FTIR spectrum of the target compound (**4i**)

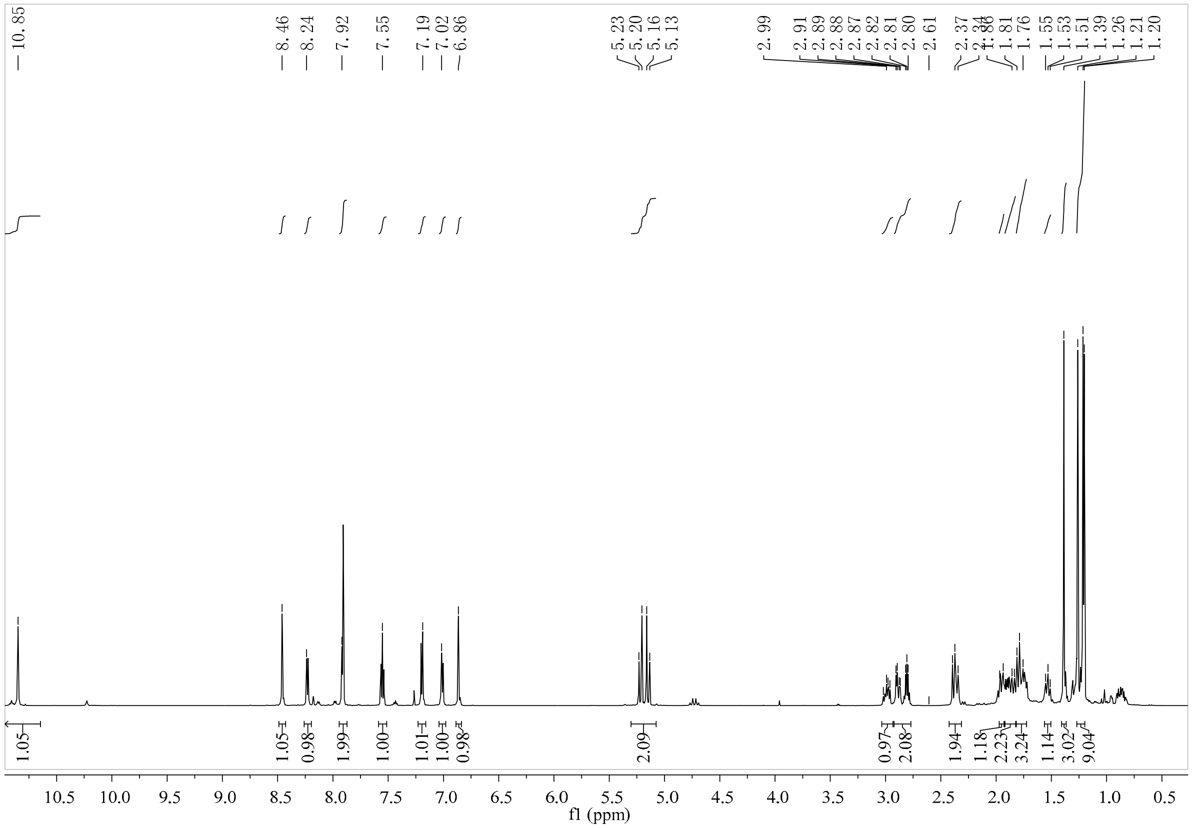

Figure S41. ^{1}H-NMR spectrum of the target compound (**4i**) in $CDCl_3$

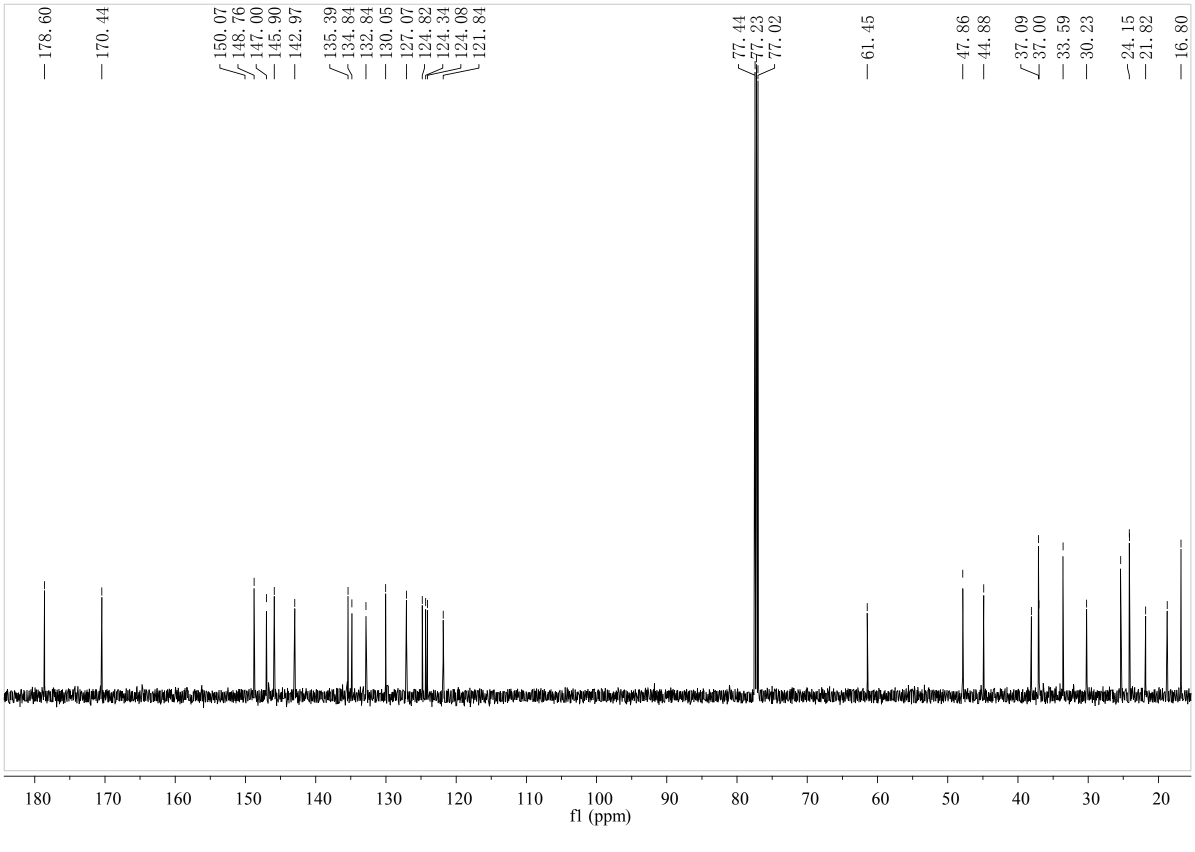

Figure S42. ^{13}C-NMR spectrum of the target compound (**4i**) in $CDCl_3$

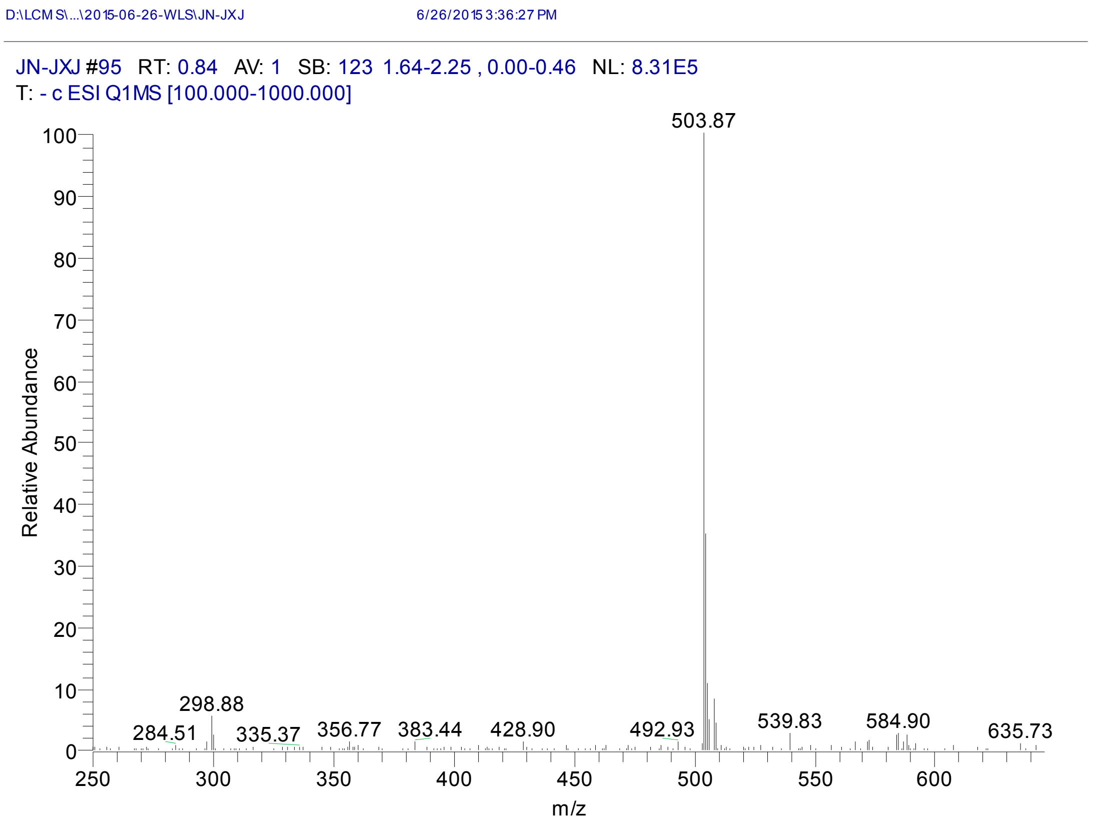

Figure S43. ESI-MS spectrum of the target compound (**4i**)

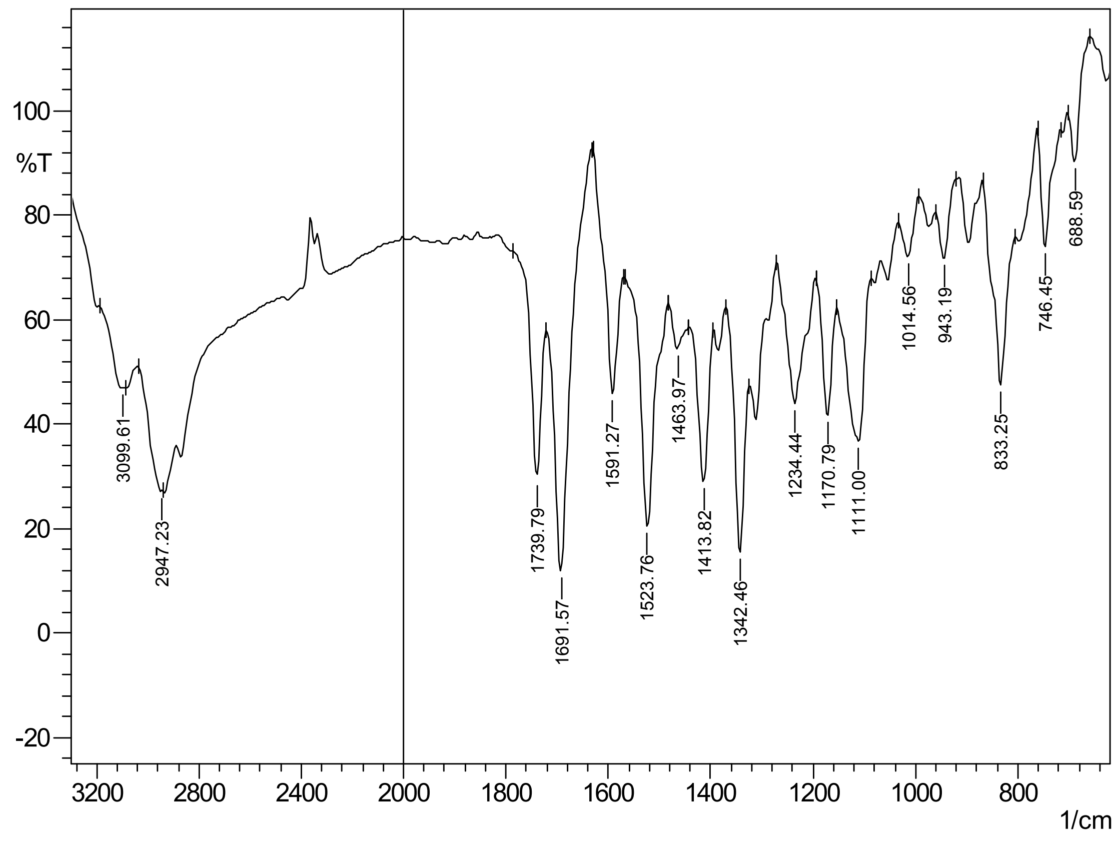

Figure S44. FTIR spectrum of the target compound (**4j**)

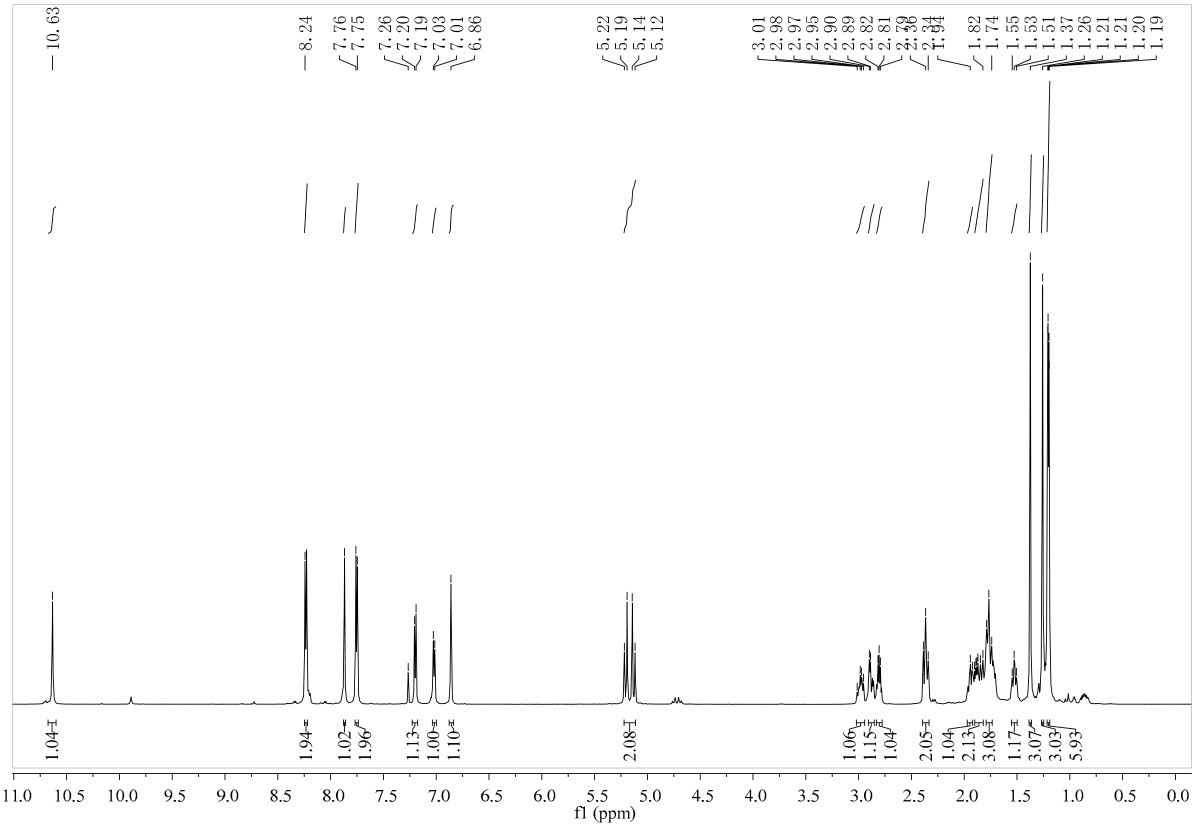

Figure S45. ^{1}H-NMR spectrum of the target compound (**4j**) in $CDCl_3$

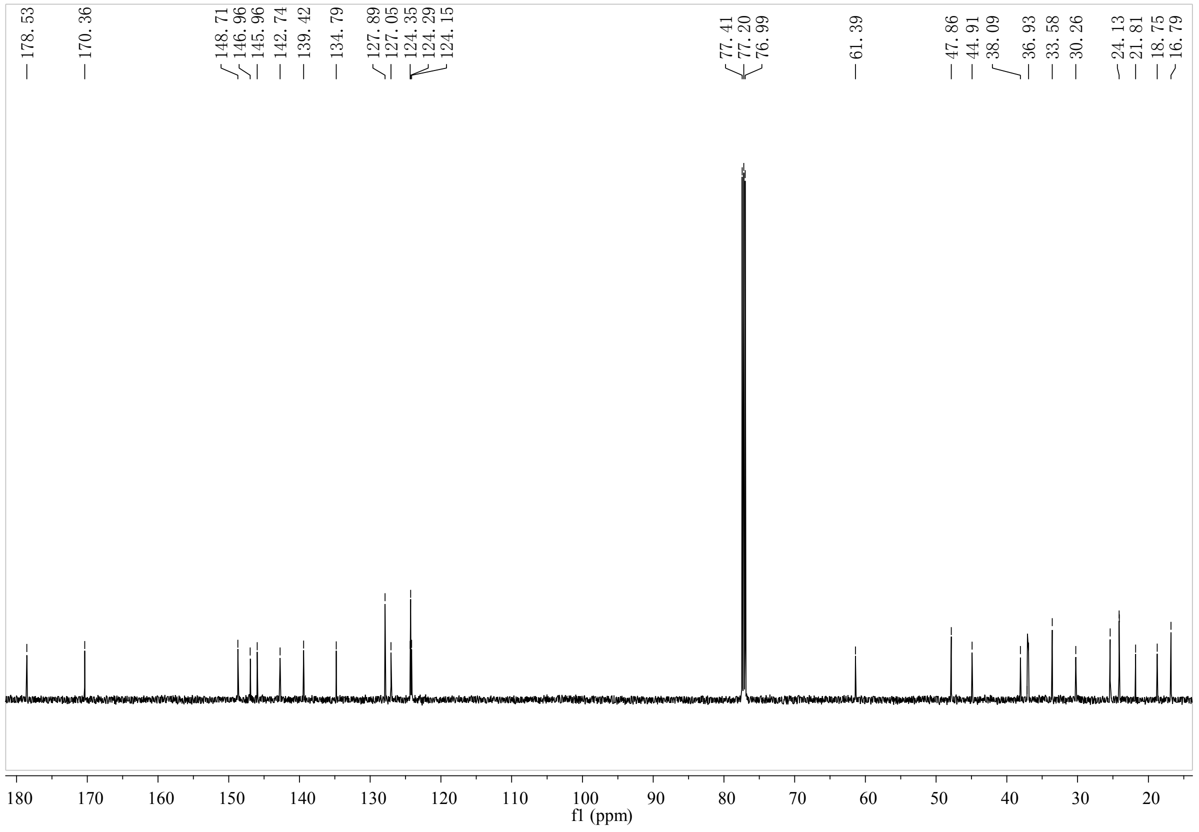

Figure S46. ^{13}C-NMR spectrum of the target compound (**4j**) in $CDCl_3$

D:\LCMS\...\2015-06-26-WLS\JN-DXJ 6/26/2015 4:05:05 PM

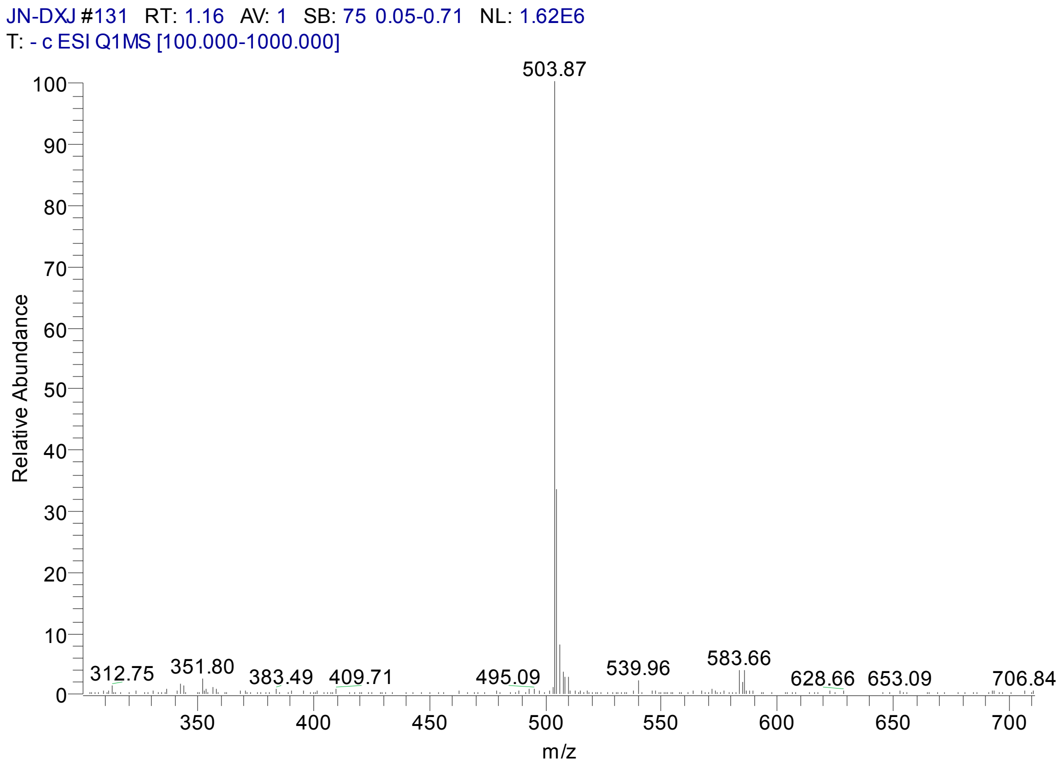

Figure S47. ESI-MS spectrum of the target compound (**4j**)

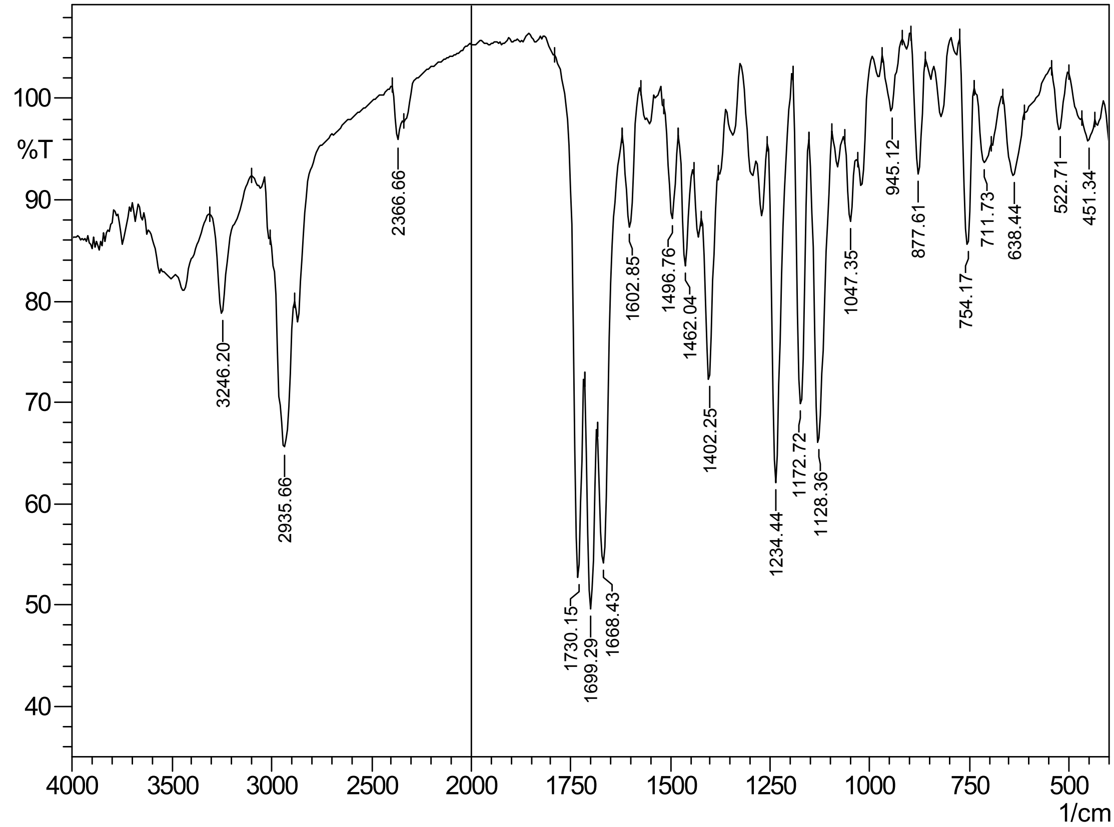

Figure S48. FTIR spectrum of the target compound (**4k**)

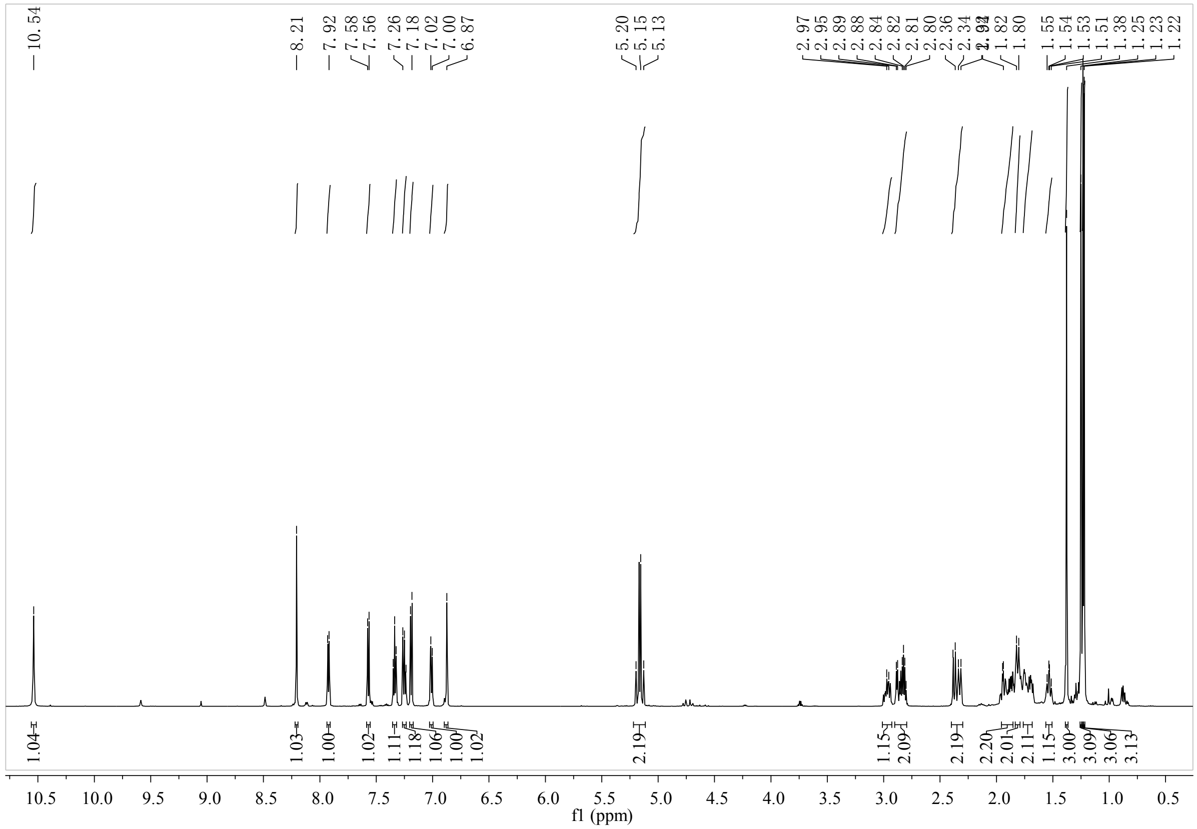

Figure S49. ^{1}H-NMR spectrum of the target compound (**4k**) in $CDCl_3$

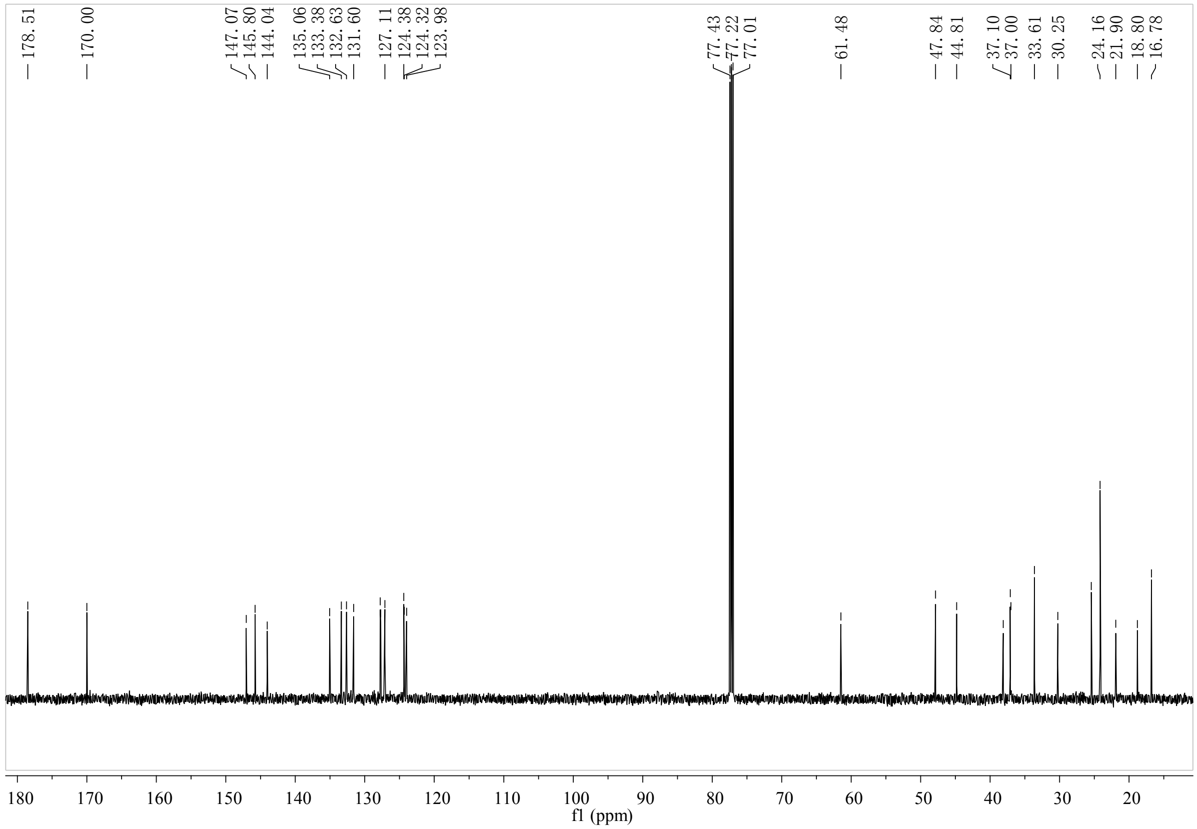

Figure S50. ^{13}C-NMR spectrum of the target compound (**4k**) in $CDCl_3$

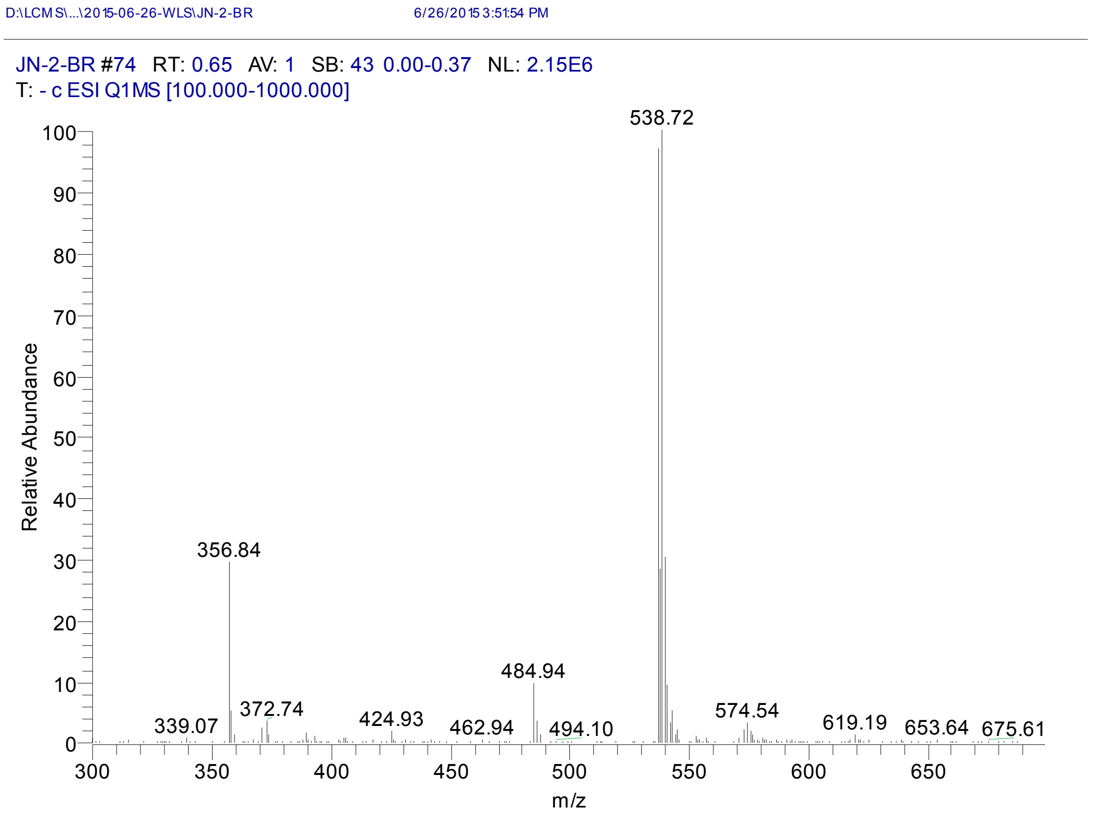

Figure S51. ESI-MS spectrum of the target compound (**4k**)

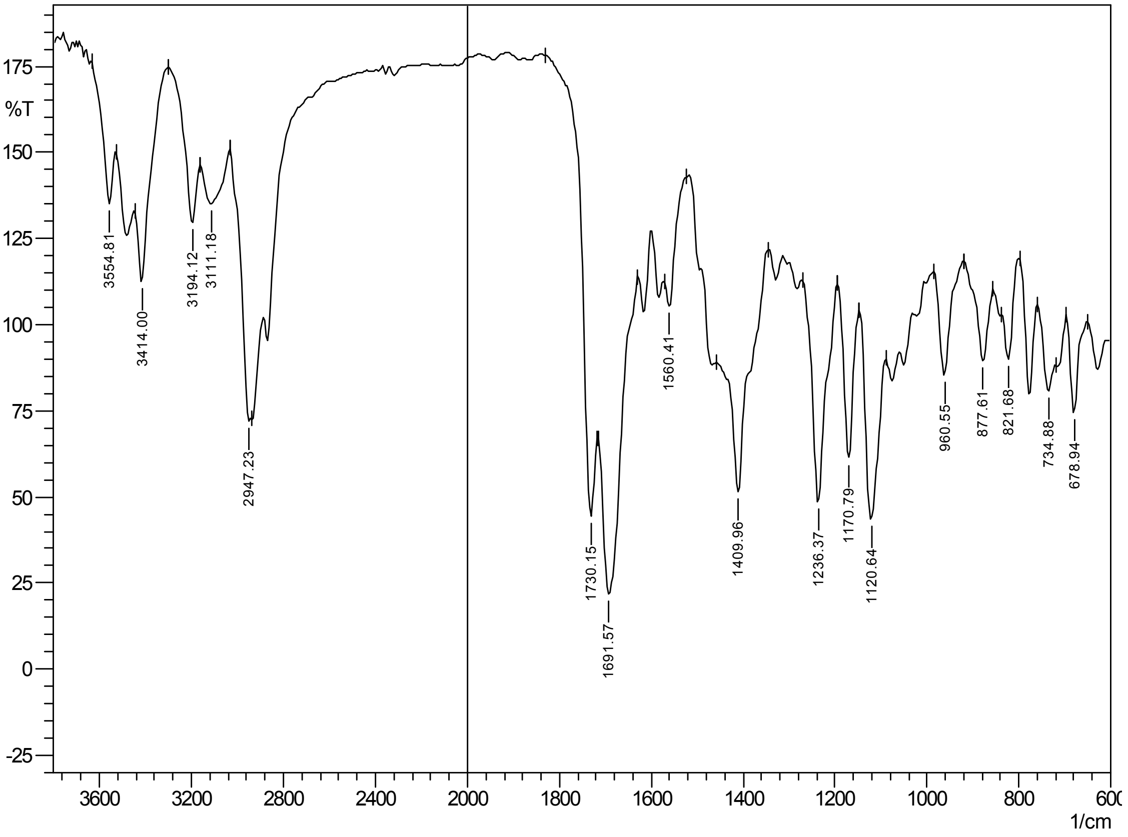

Figure S52. FTIR spectrum of the target compound (**4l**)

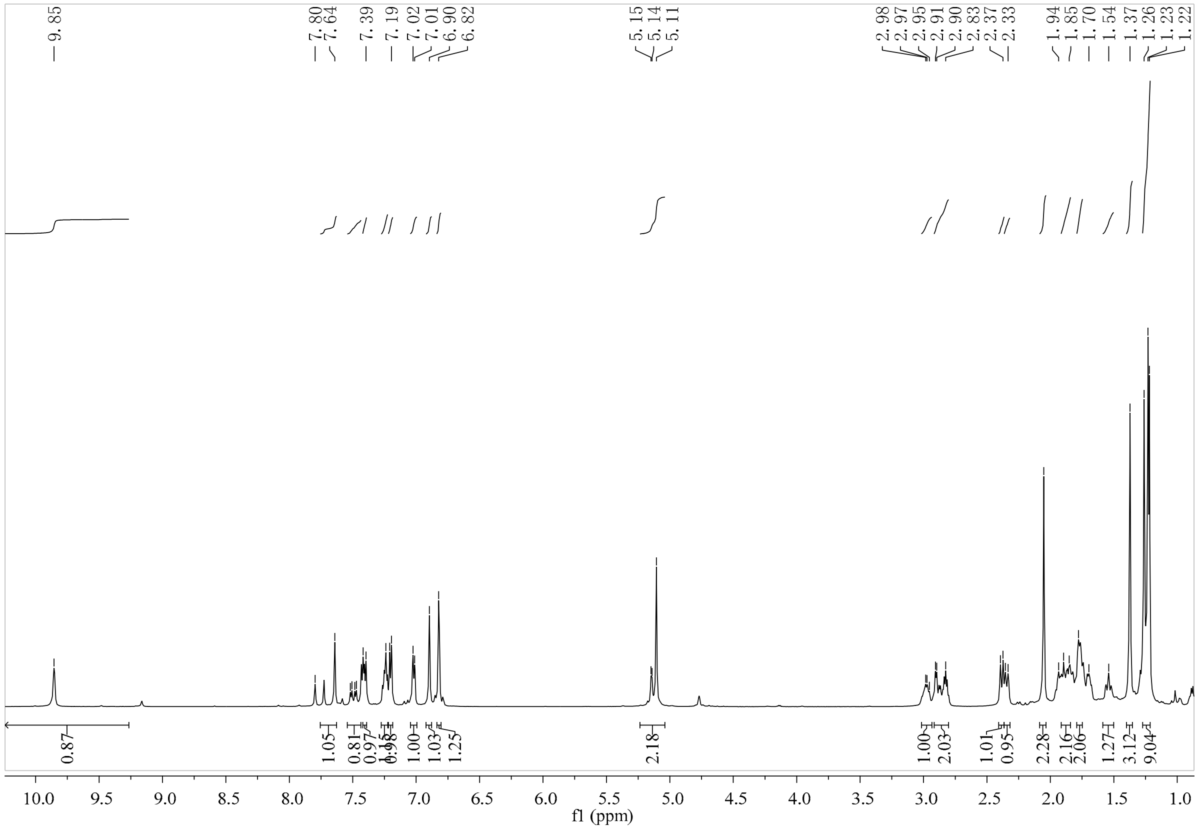

Figure S53. ^{1}H-NMR spectrum of the target compound (**4l**) in $CDCl_3$

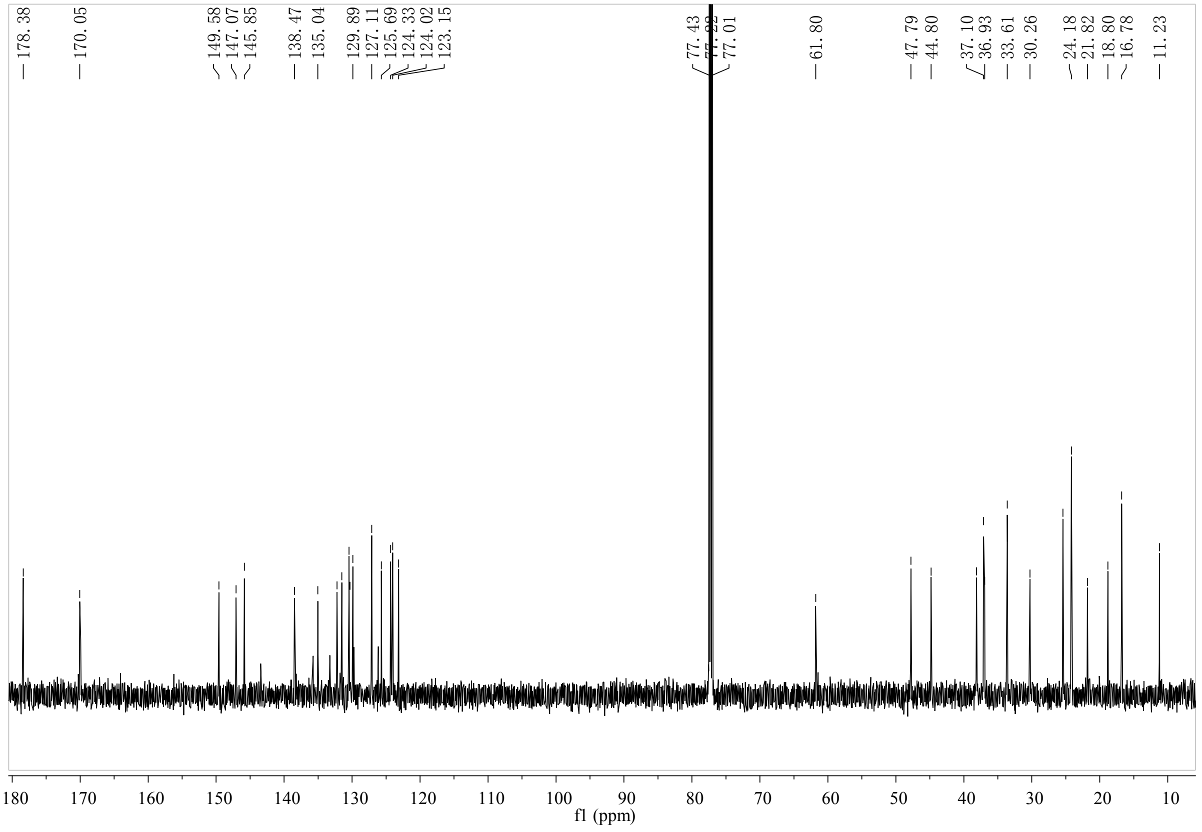

Figure S54. ^{13}C-NMR spectrum of the target compound (**4l**) in $CDCl_3$

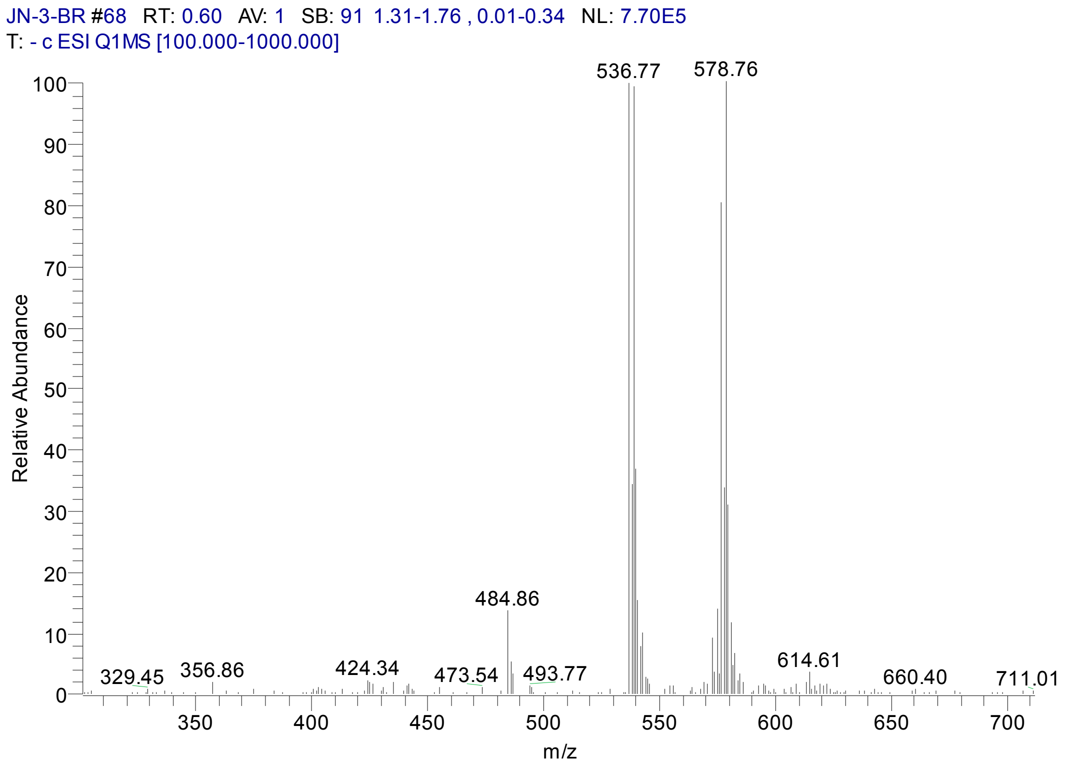

Figure S55. ESI-MS spectrum of the target compound (**4l**)

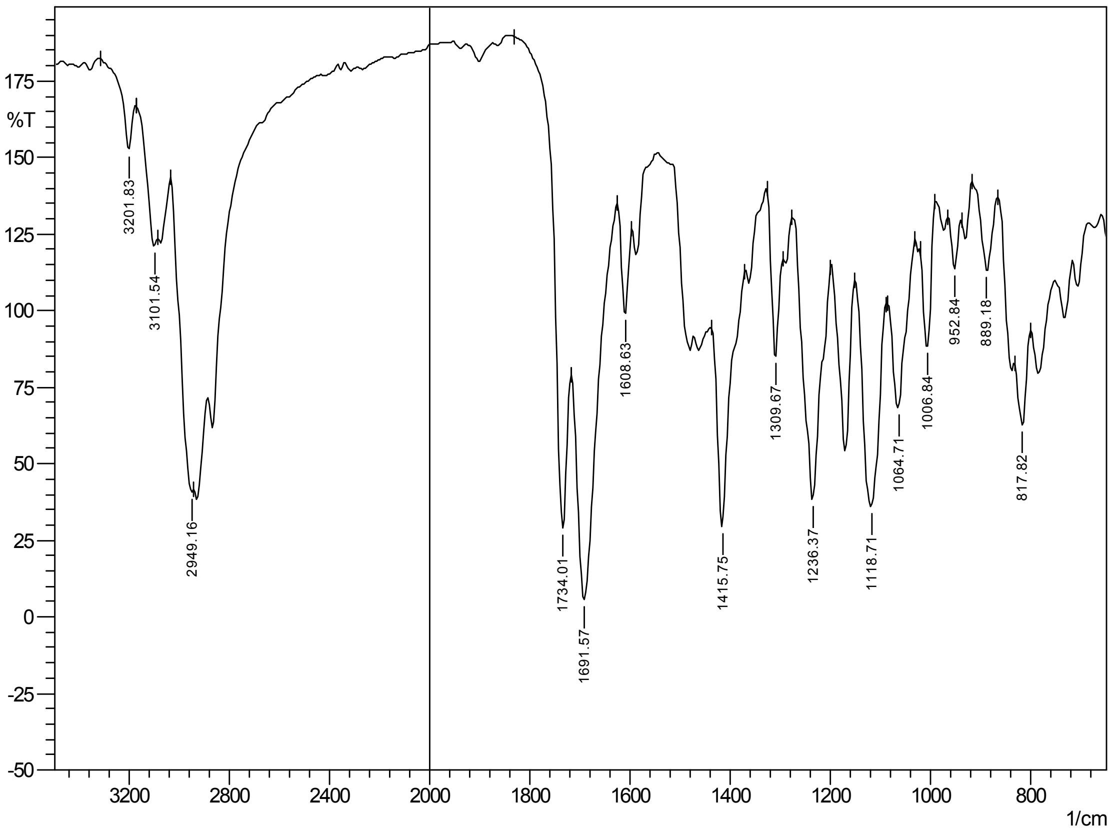

Figure S56. FTIR spectrum of the target compound (**4m**)

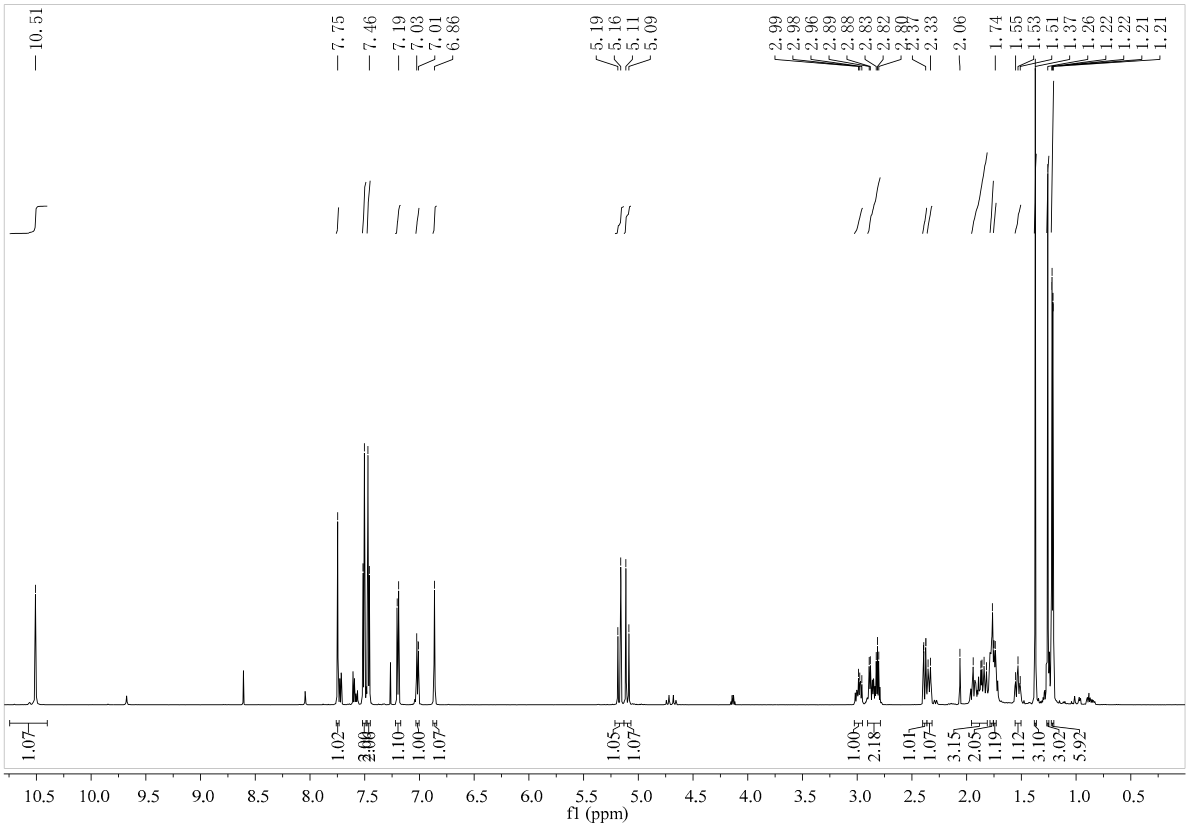

Figure S57. ^{1}H-NMR spectrum of the target compound (**4m**) in $CDCl_3$

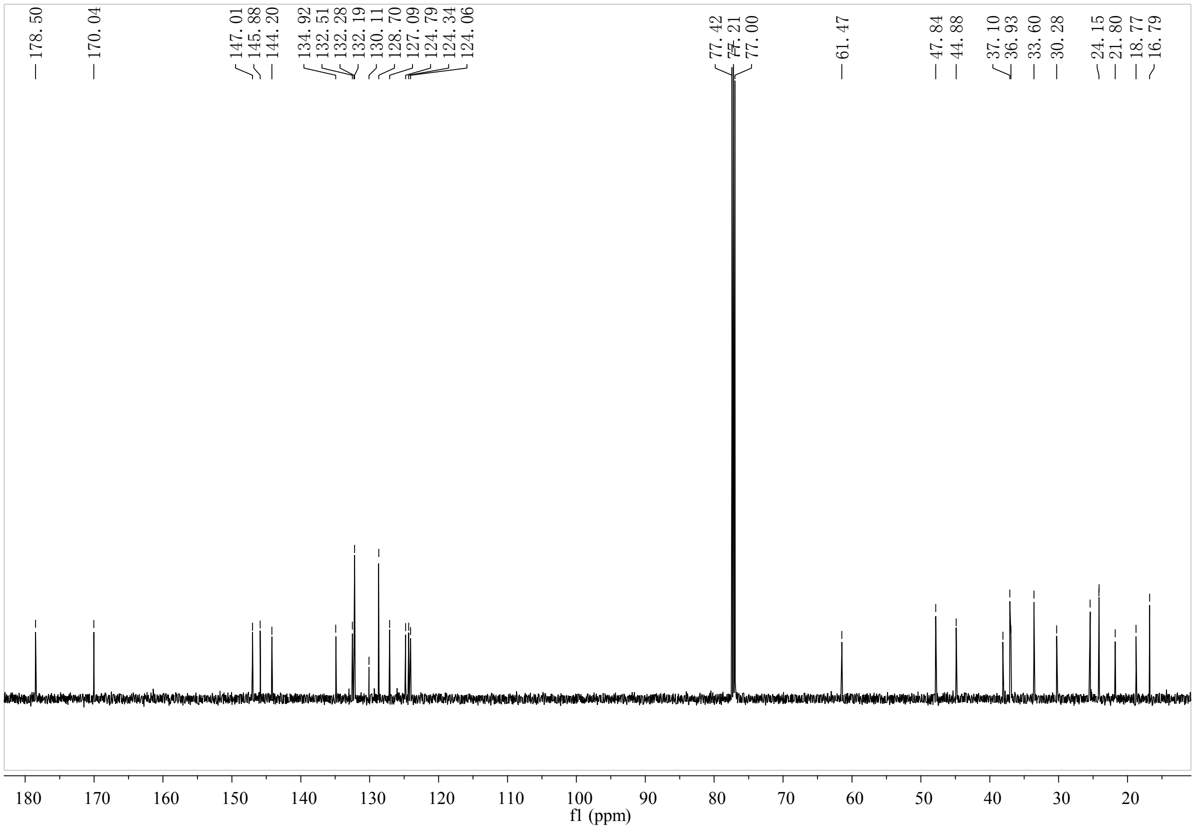

Figure S58. ^{13}C-NMR spectrum of the target compound (**4m**) in $CDCl_3$

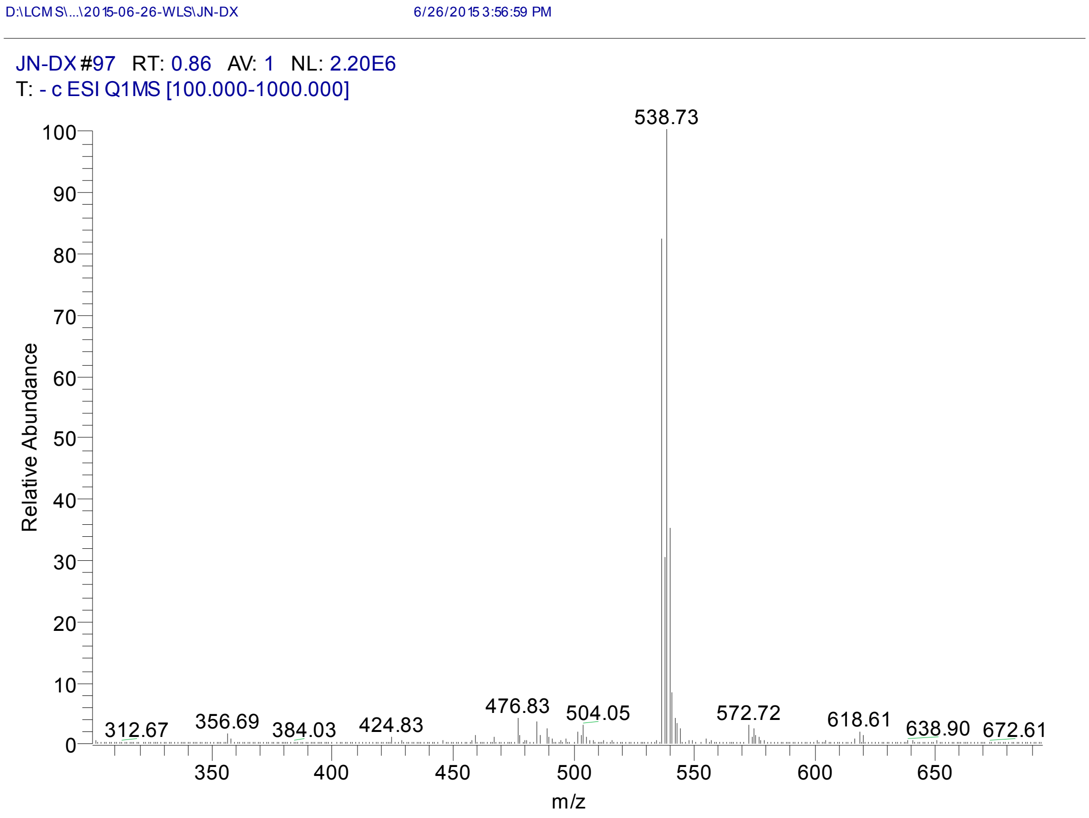

Figure S59. ESI-MS spectrum of the target compound (**4m**)

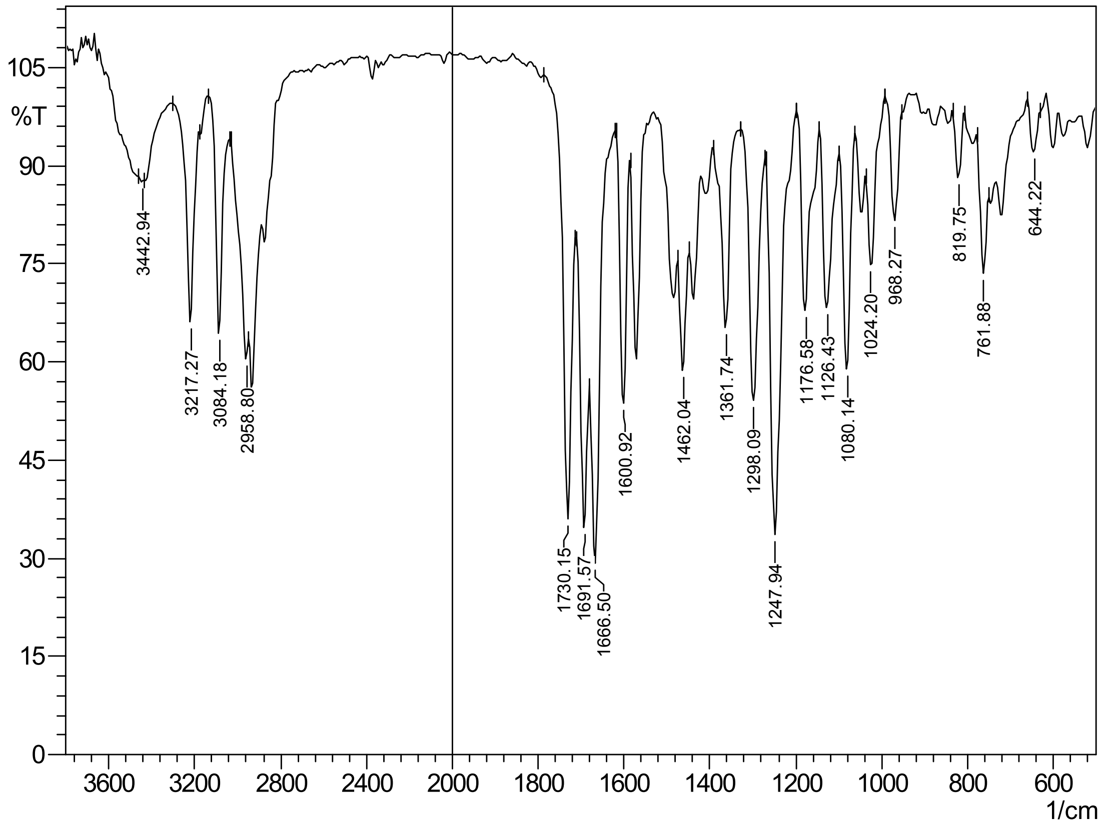

Figure S60. FTIR spectrum of the target compound (**4n**)

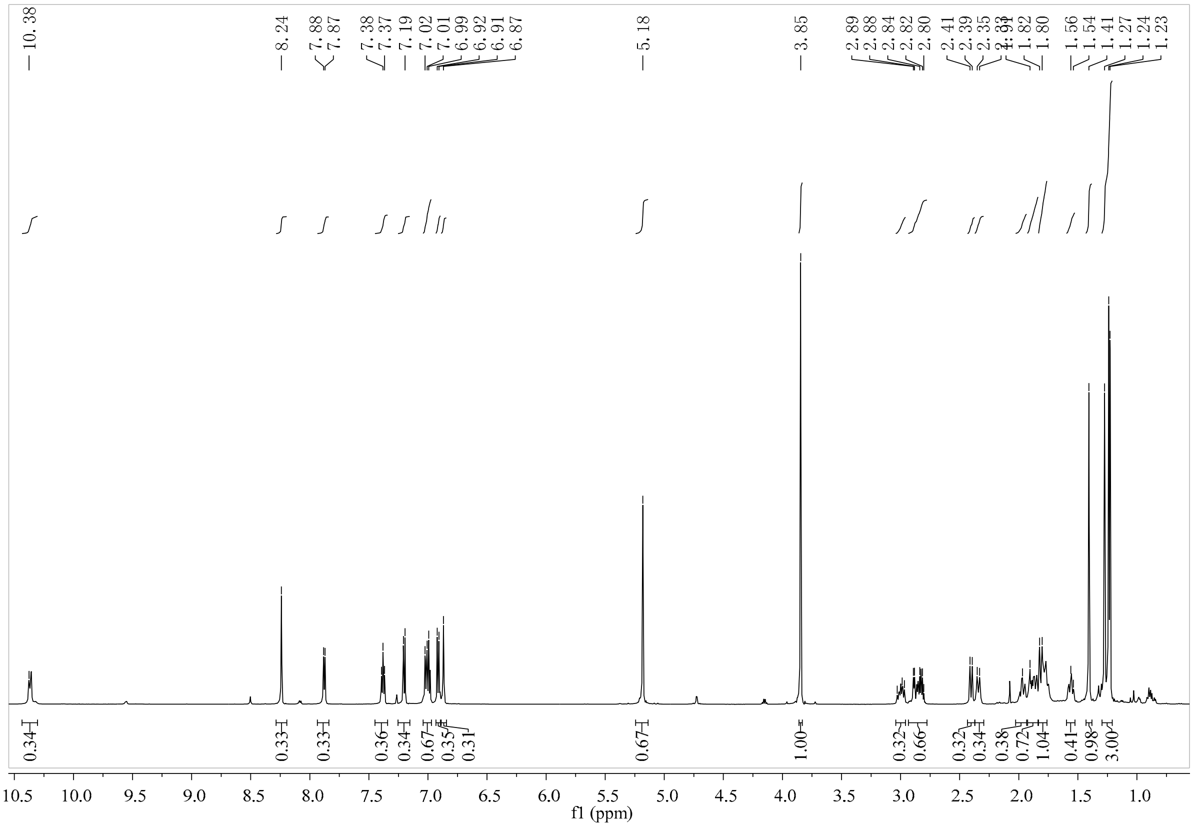

Figure S61. ^{1}H-NMR spectrum of the target compound (**4n**) in $CDCl_3$

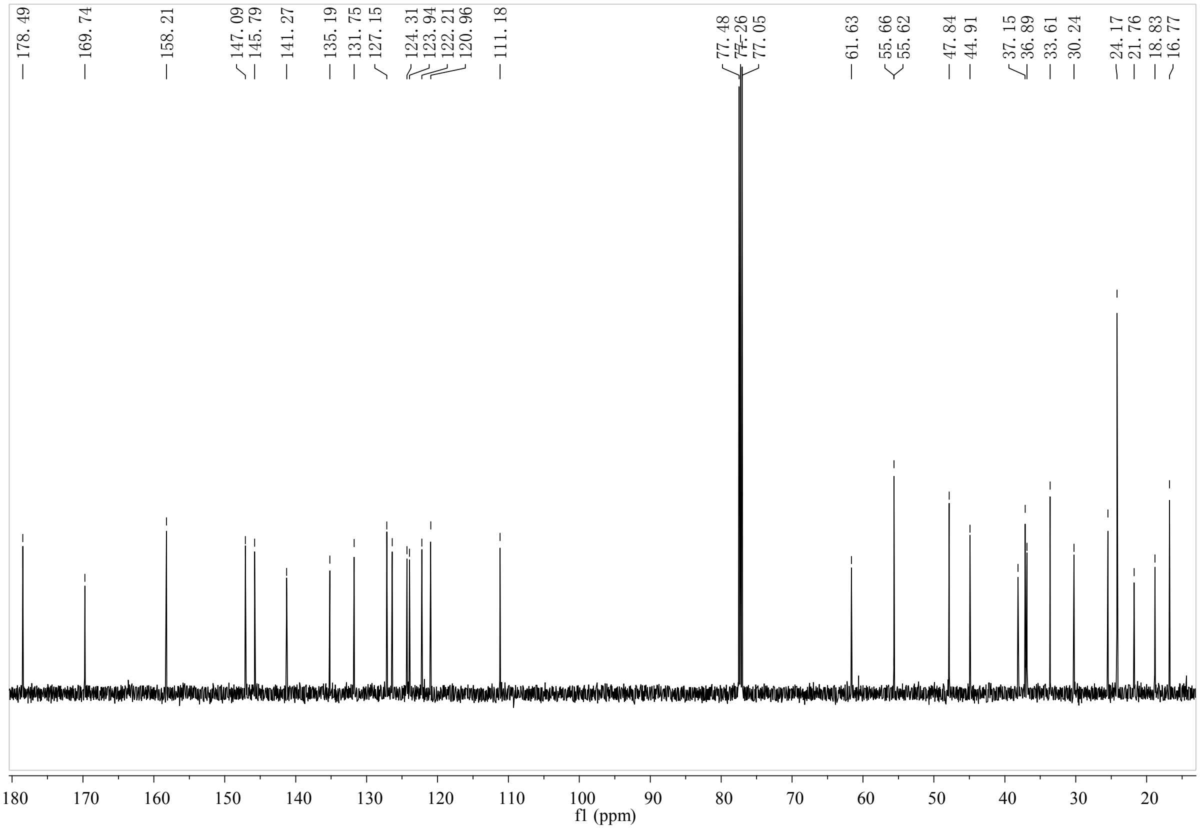

Figure S62. ^{13}C-NMR spectrum of the target compound (**4n**) in $CDCl_3$

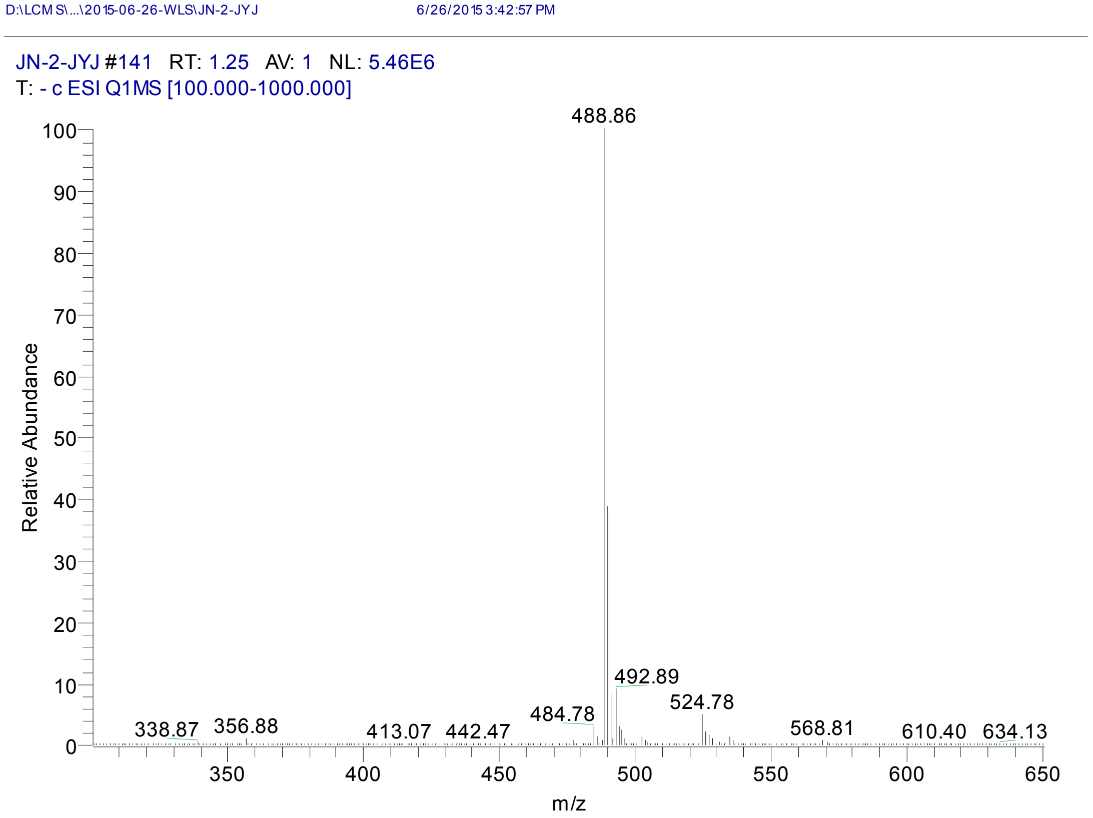

Figure S63. ESI-MS spectrum of the target compound (**4n**)

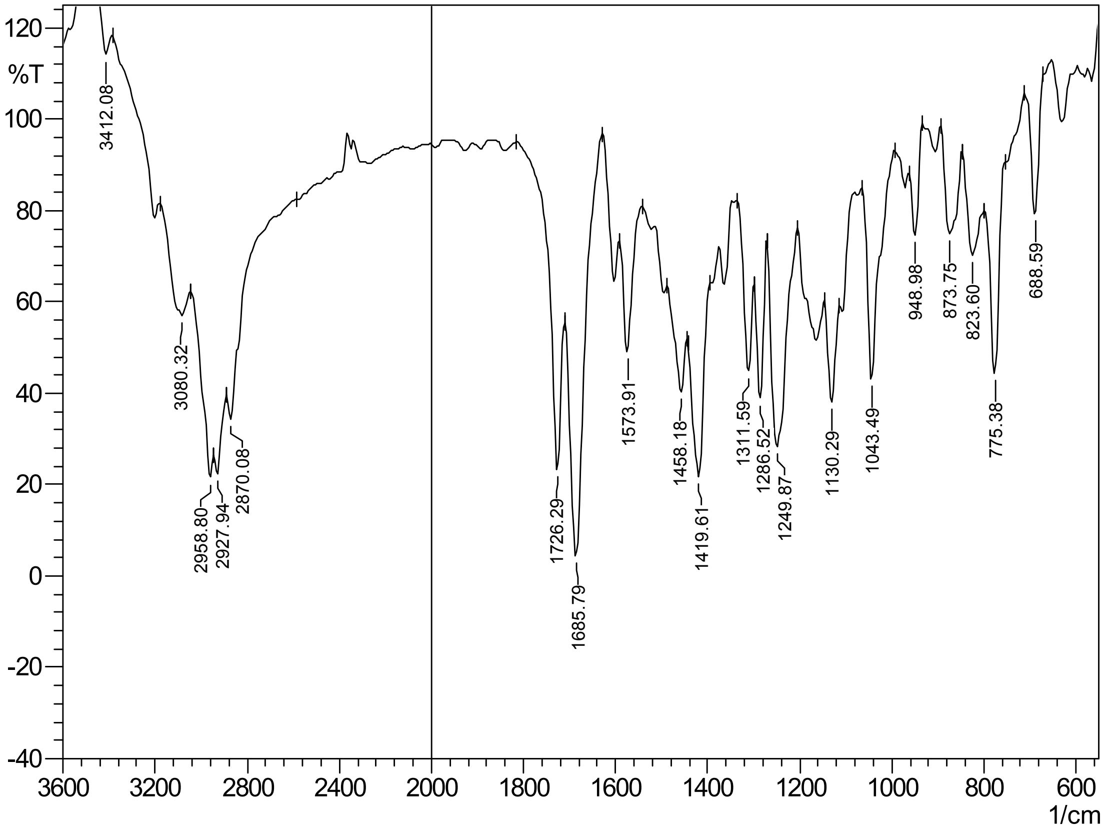

Figure S64. FTIR spectrum of the target compound (**4o**)

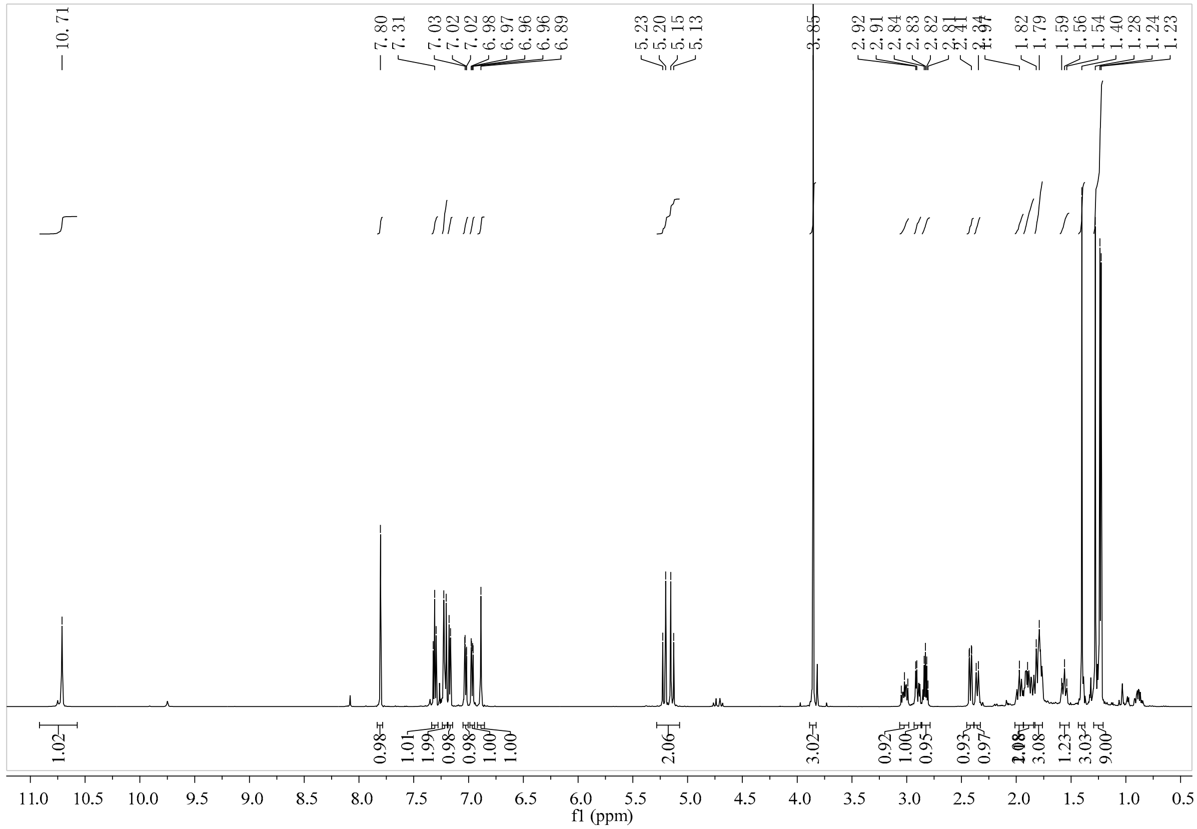

Figure S65. ^{1}H-NMR spectrum of the target compound (**4o**) in $CDCl_3$

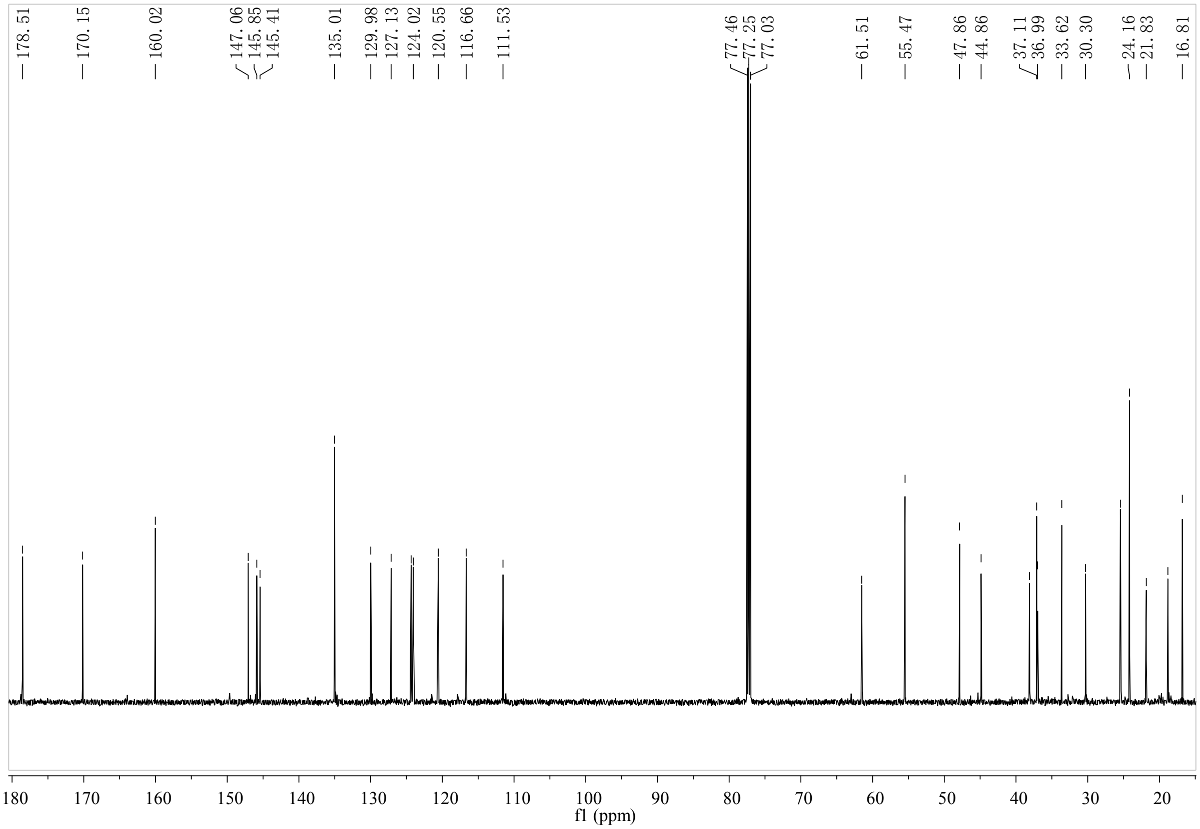

Figure S66. ^{13}C-NMR spectrum of the target compound (**4o**) in $CDCl_3$

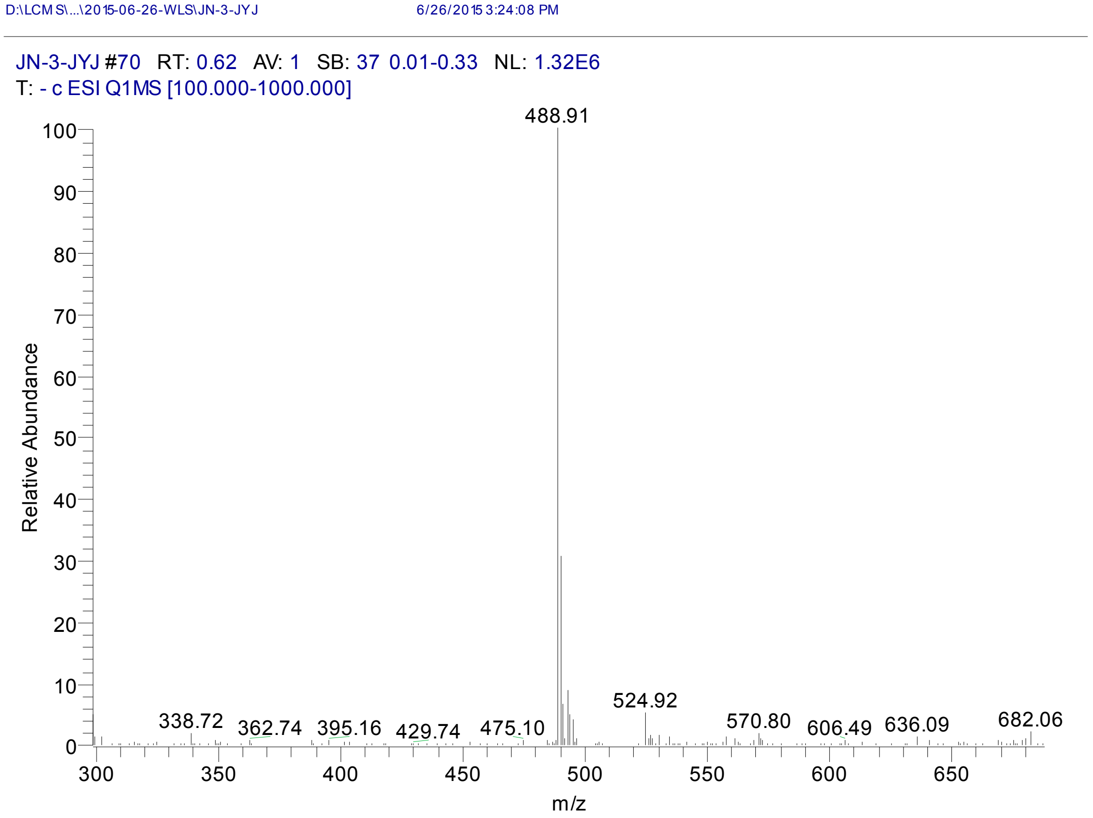

Figure S67. ESI-MS spectrum of the target compound (**4o**)

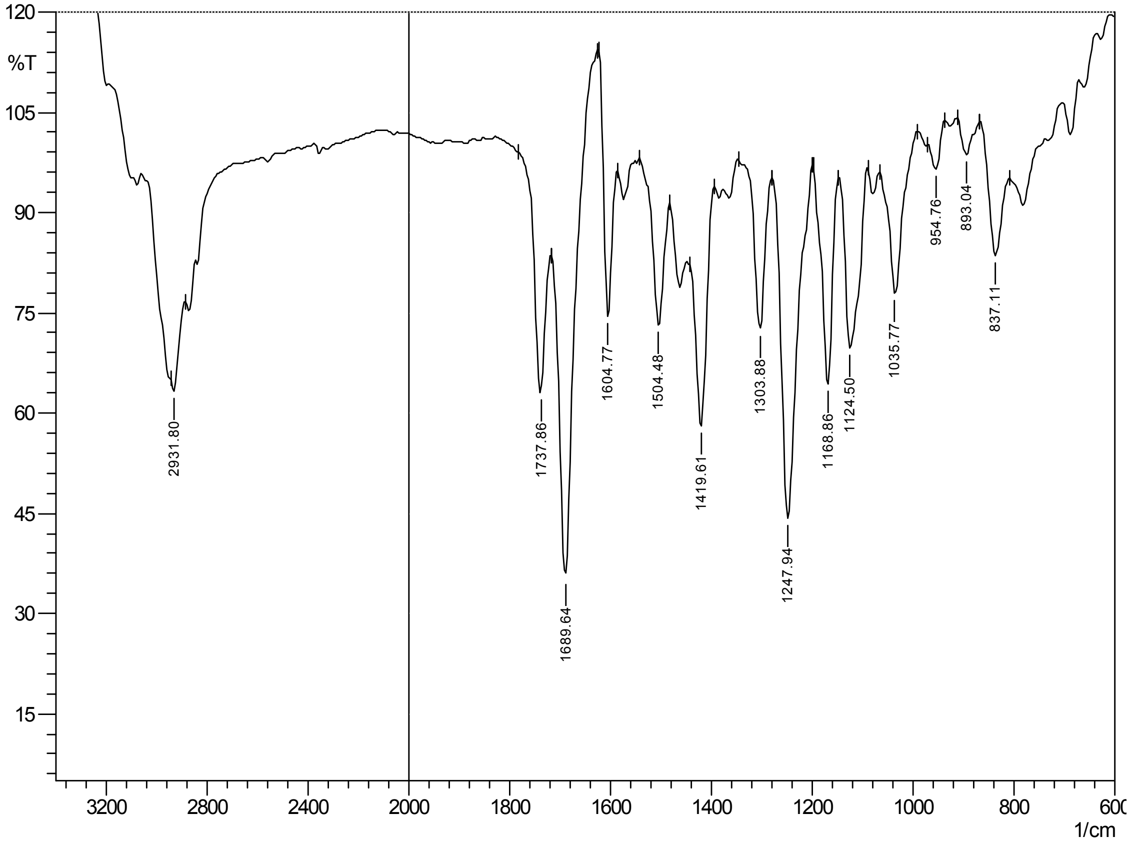

Figure S68. FTIR spectrum of the target compound (**4p**)

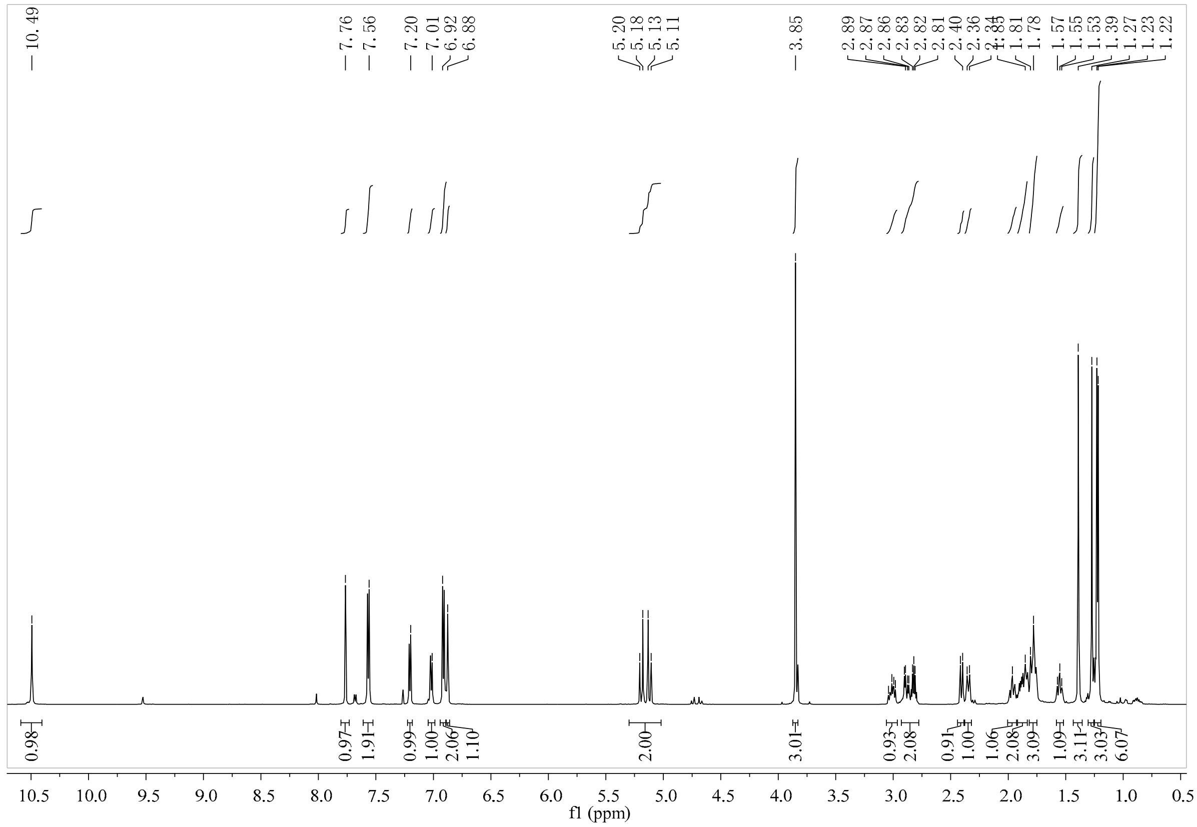

Figure S69. ^{1}H-NMR spectrum of the target compound (**4p**) in $CDCl_3$

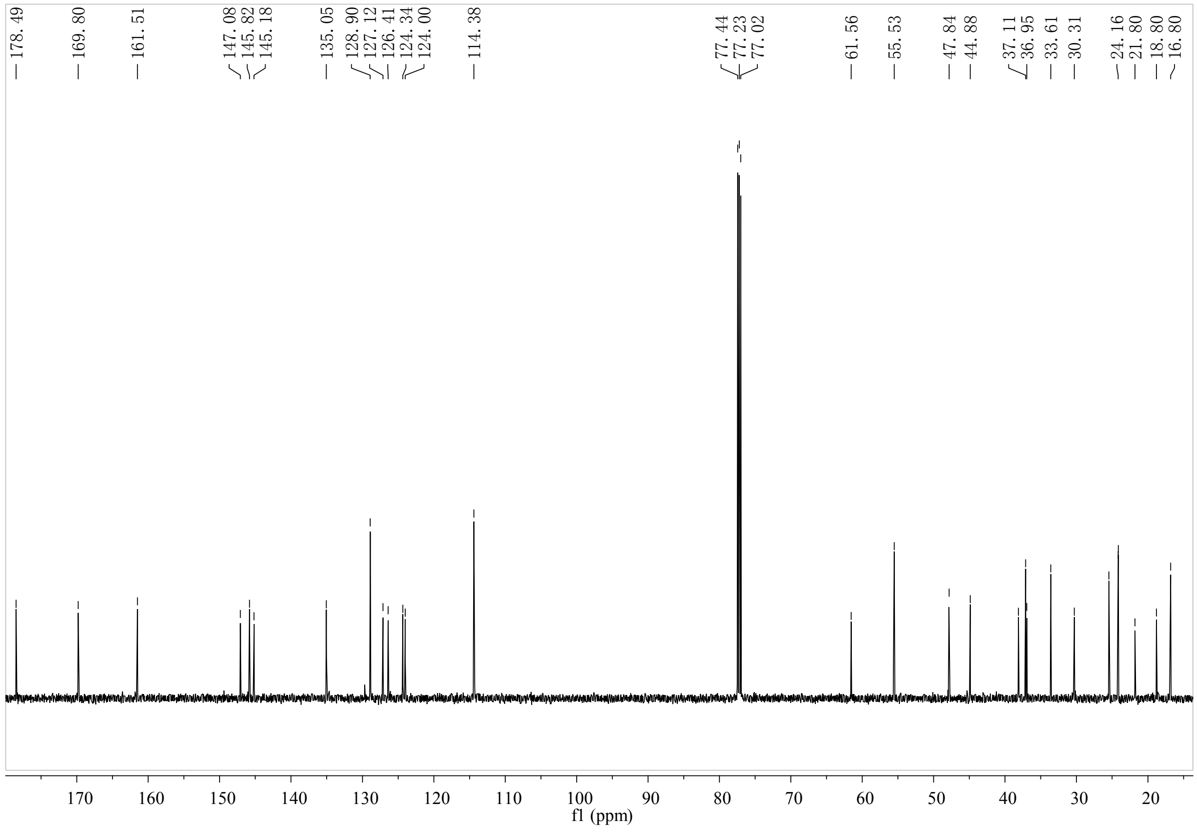

Figure S70. ^{13}C-NMR spectrum of the target compound (**4p**) in $CDCl_3$

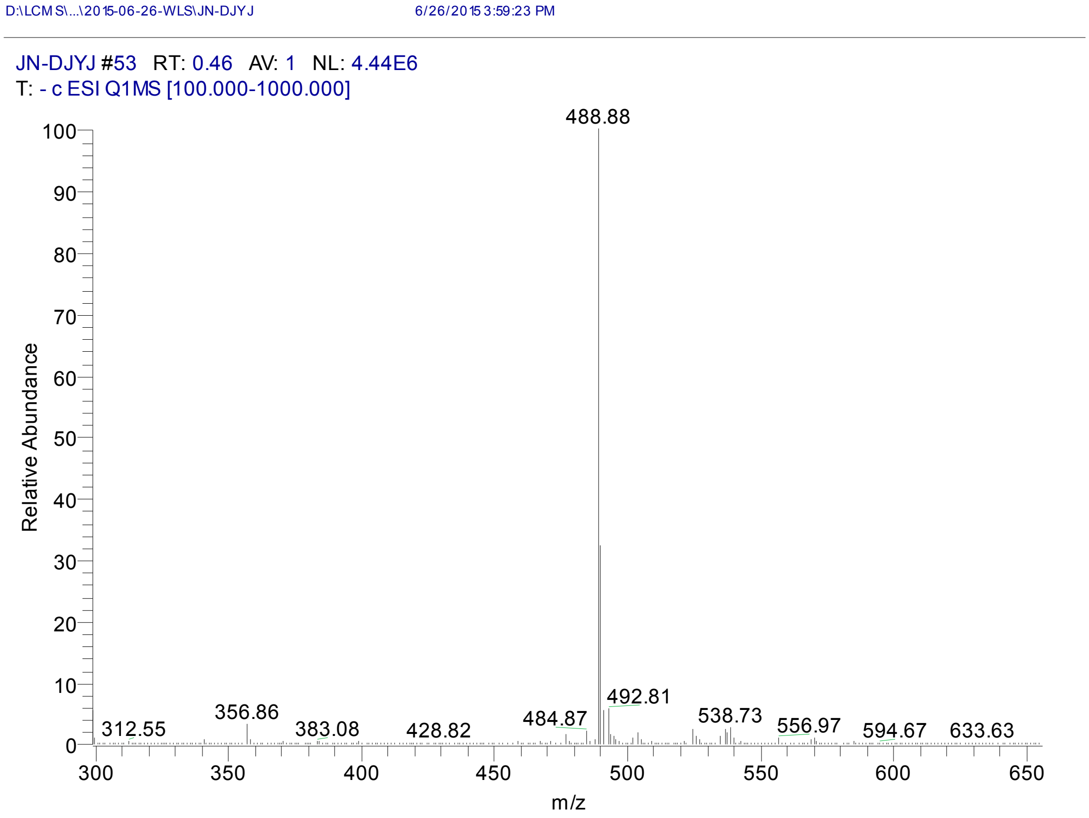

Figure S71. ESI-MS spectrum of the target compound (**4p**)

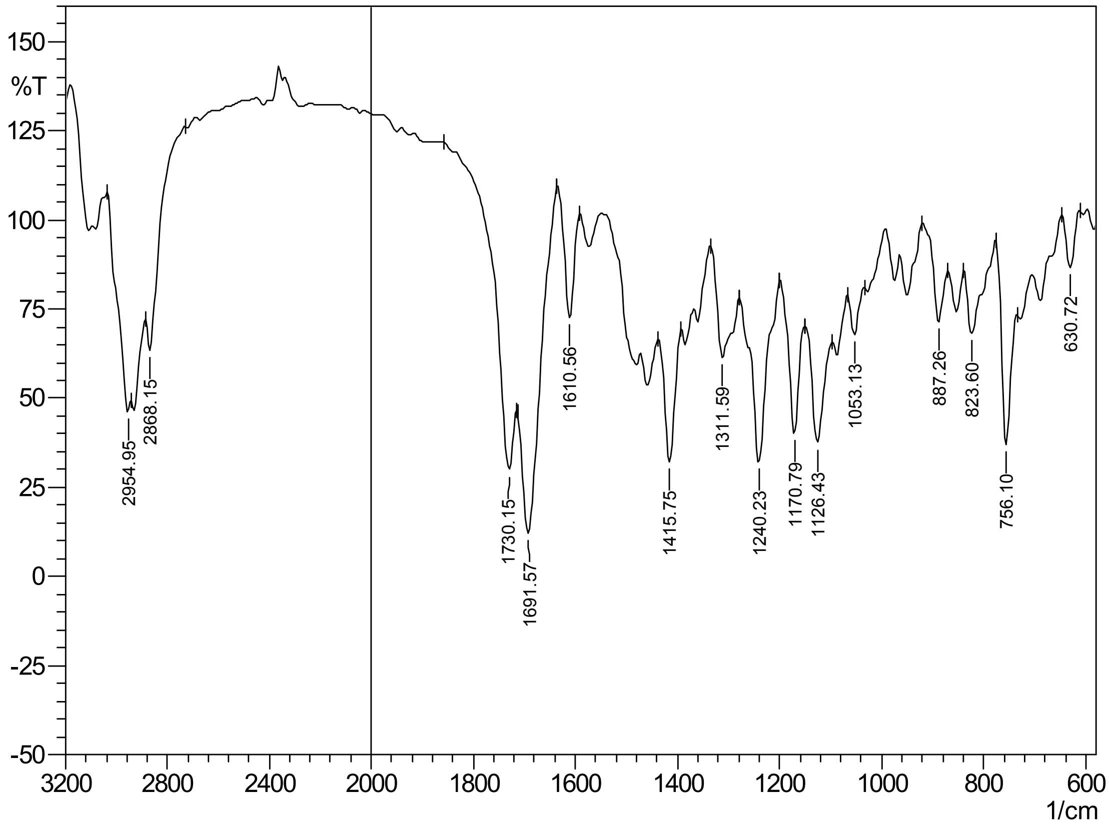

Figure S72. FTIR spectrum of the target compound (**4q**)

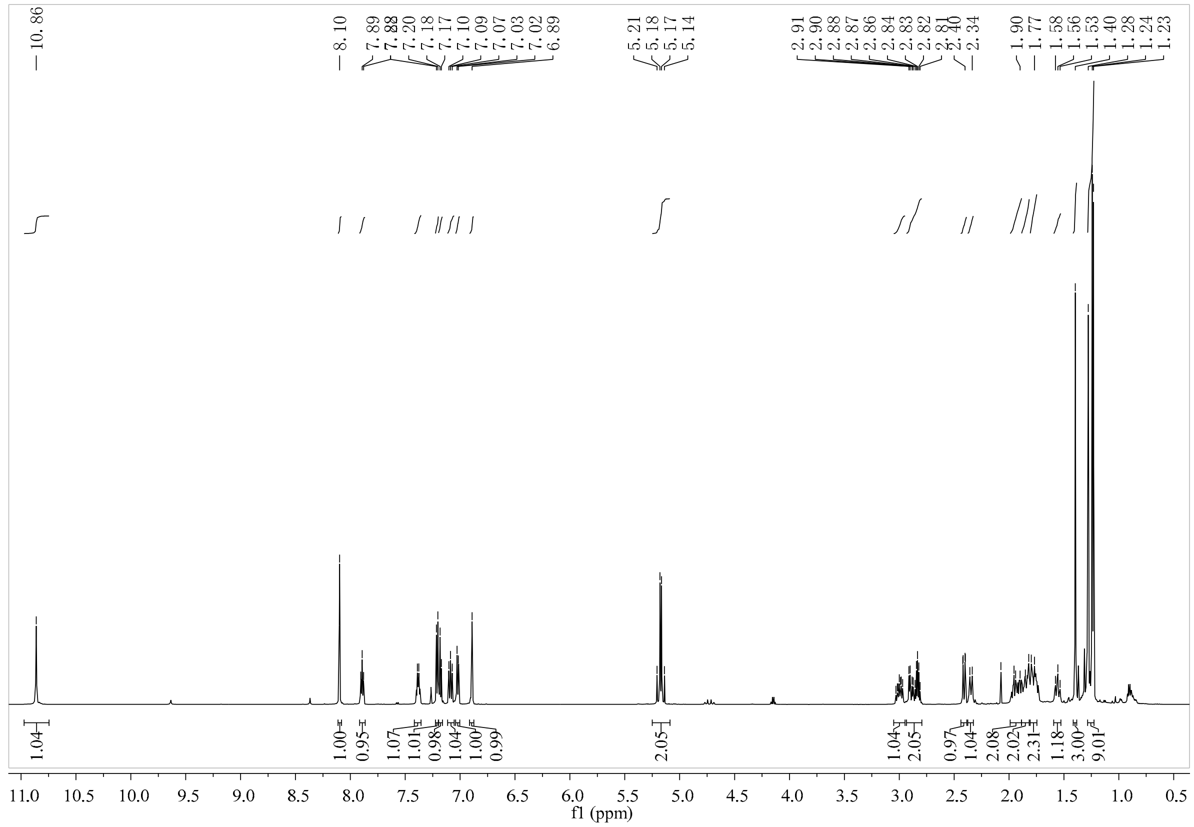

Figure S73. ^{1}H-NMR spectrum of the target compound (**4q**) in $CDCl_3$

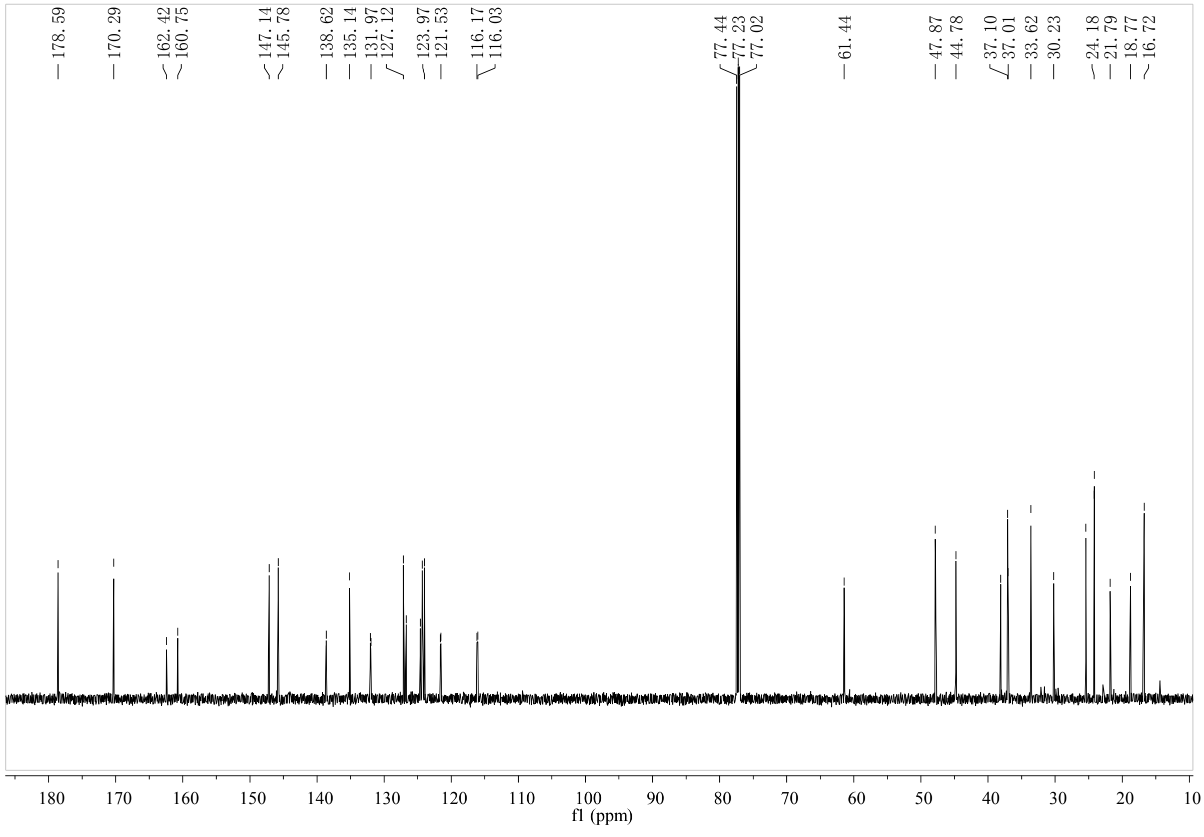

Figure S74. ^{13}C-NMR spectrum of the target compound (**4q**) in $CDCl_3$

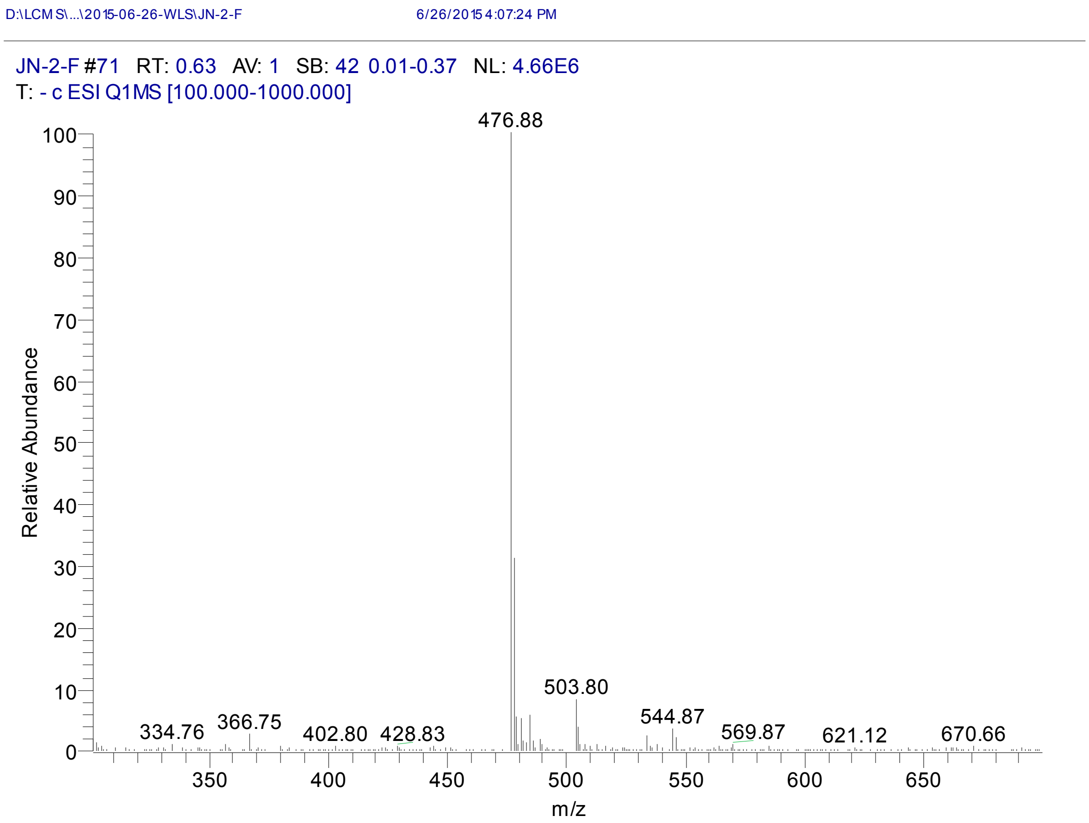

Figure S75. ESI-MS spectrum of the target compound (**4q**)

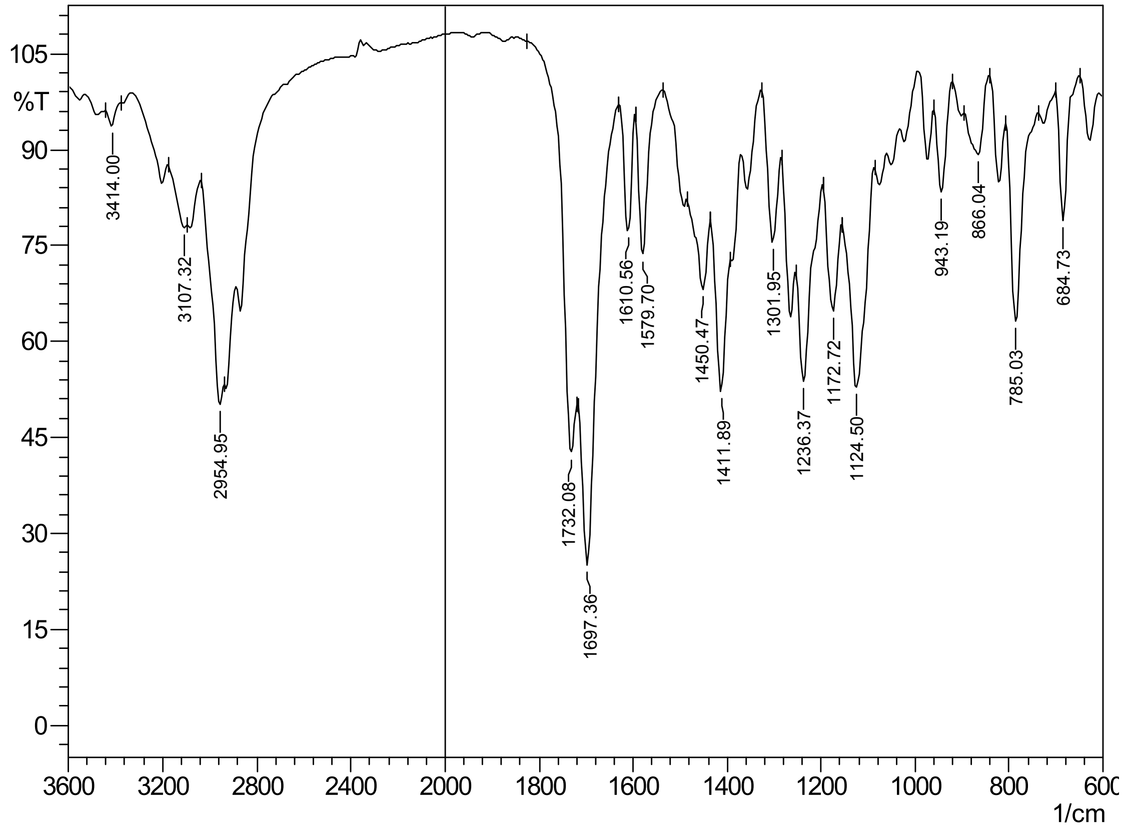

Figure S76. FTIR spectrum of the target compound (**4r**)

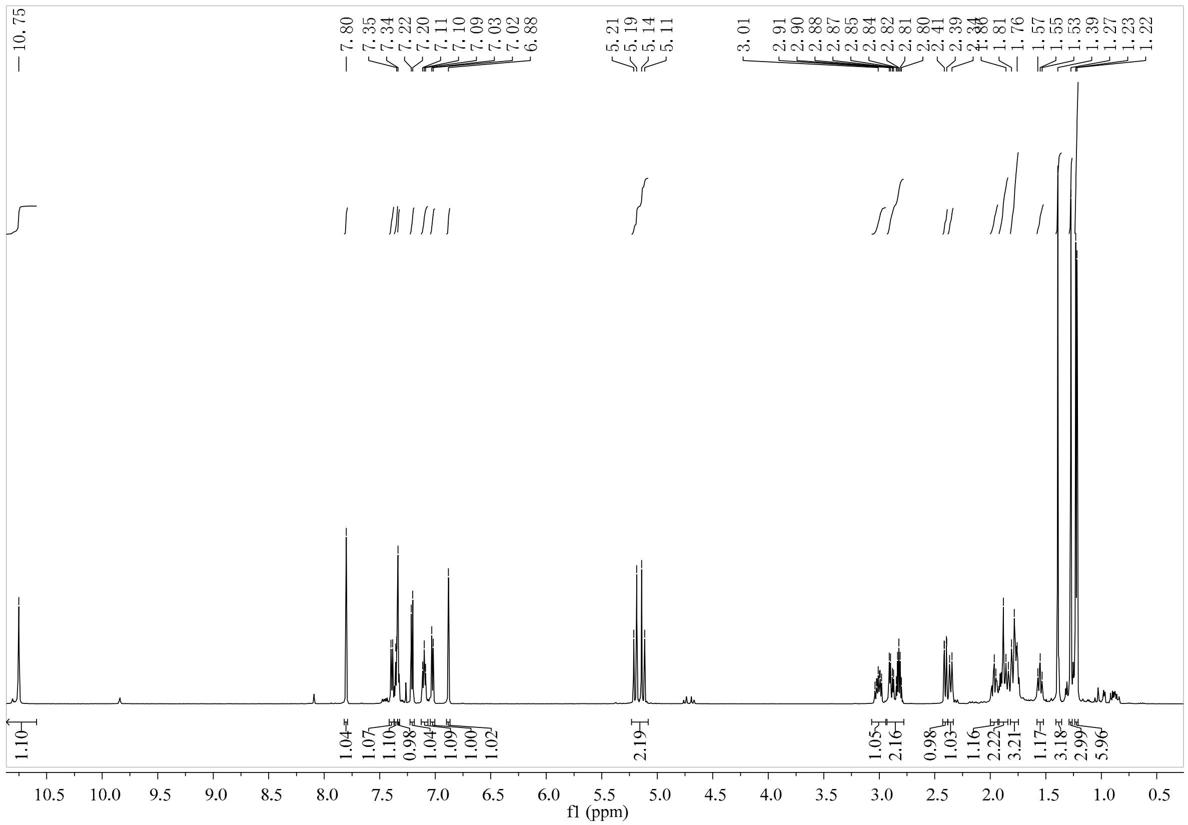

Figure S77. ^{1}H-NMR spectrum of the target compound (**4r**) in $CDCl_3$

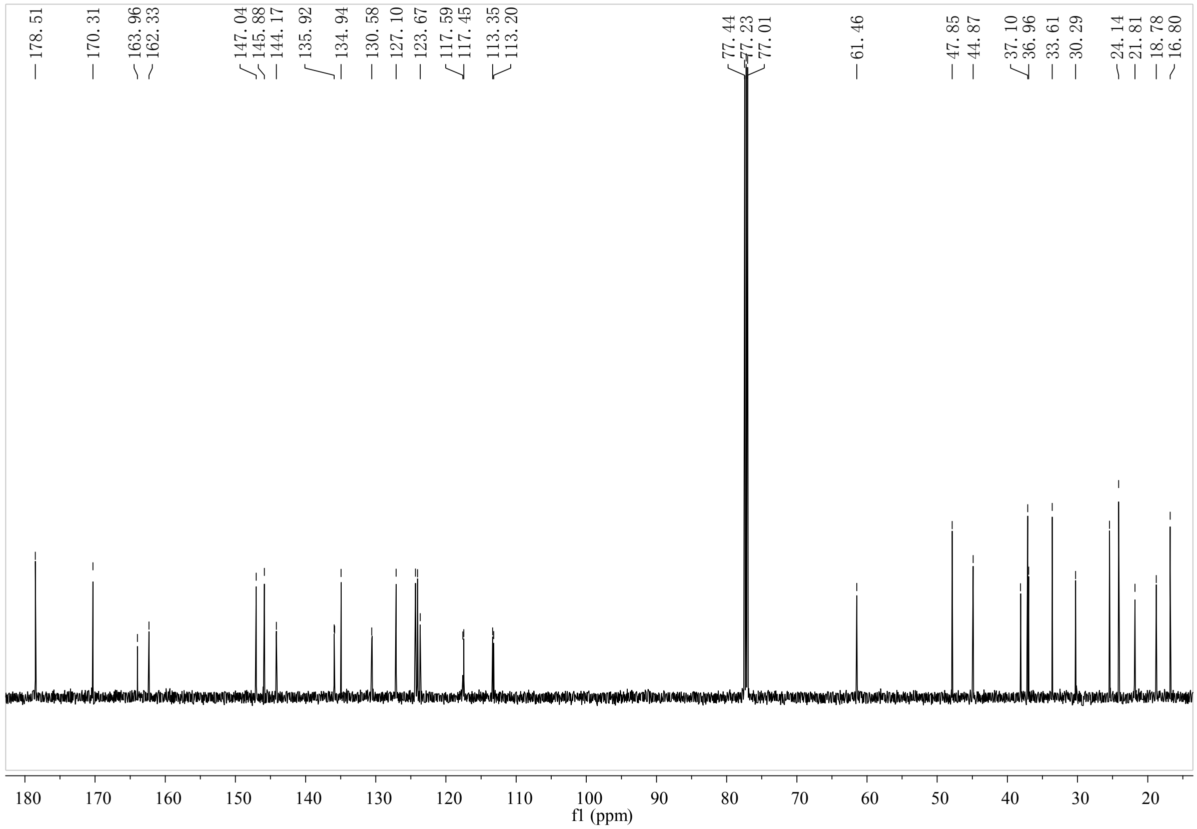

Figure S78. ^{13}C-NMR spectrum of the target compound (**4r**) in $CDCl_3$

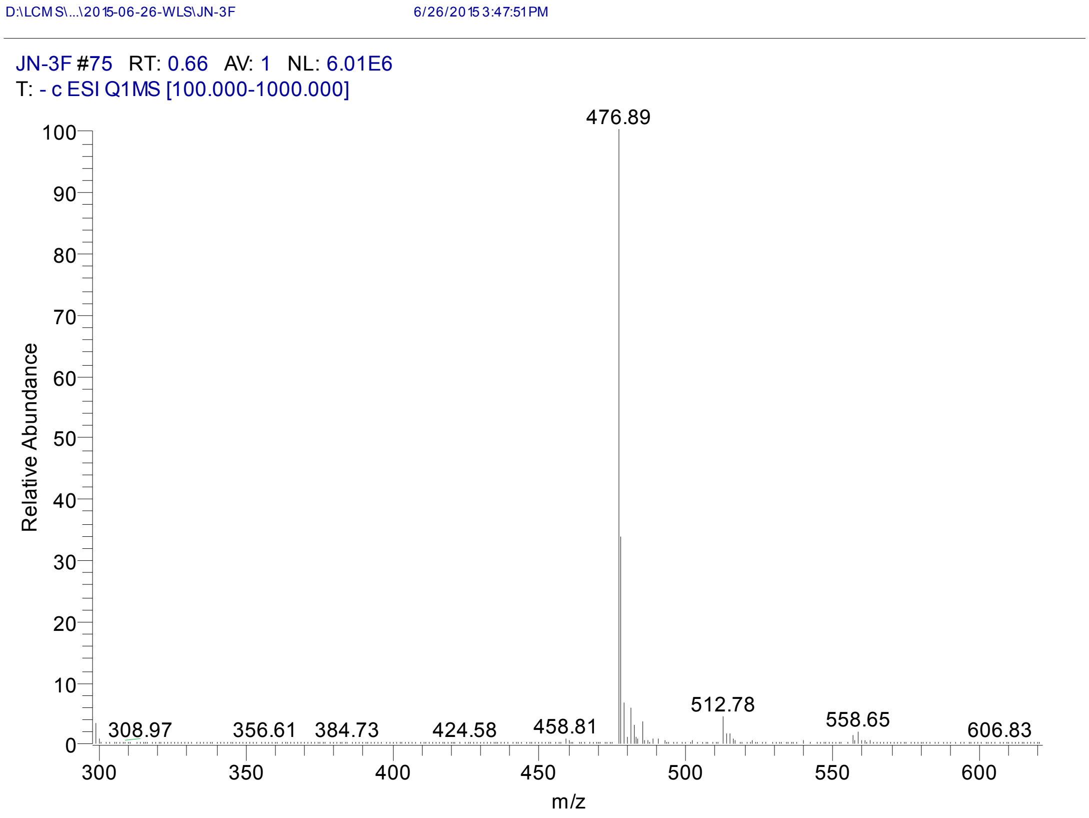

Figure S79. ESI-MS spectrum of the target compound (**4r**)

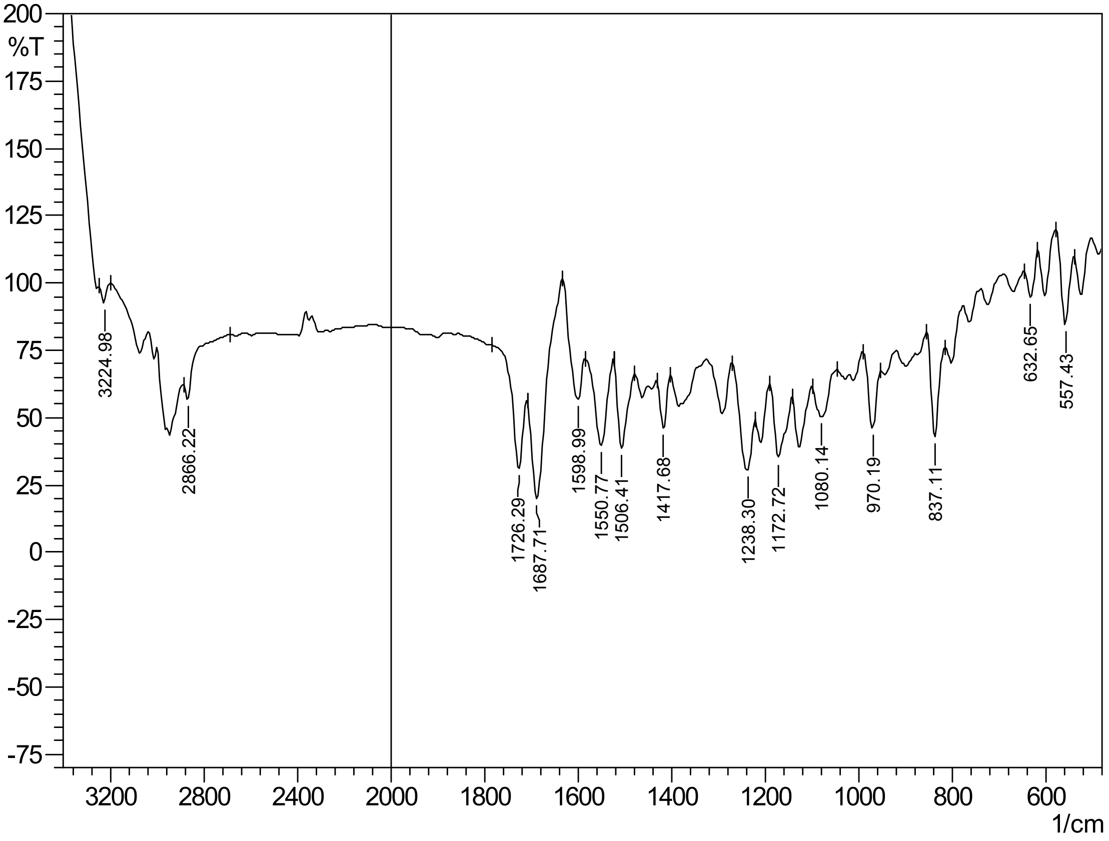

Figure S80. FTIR spectrum of the target compound (**4s**)

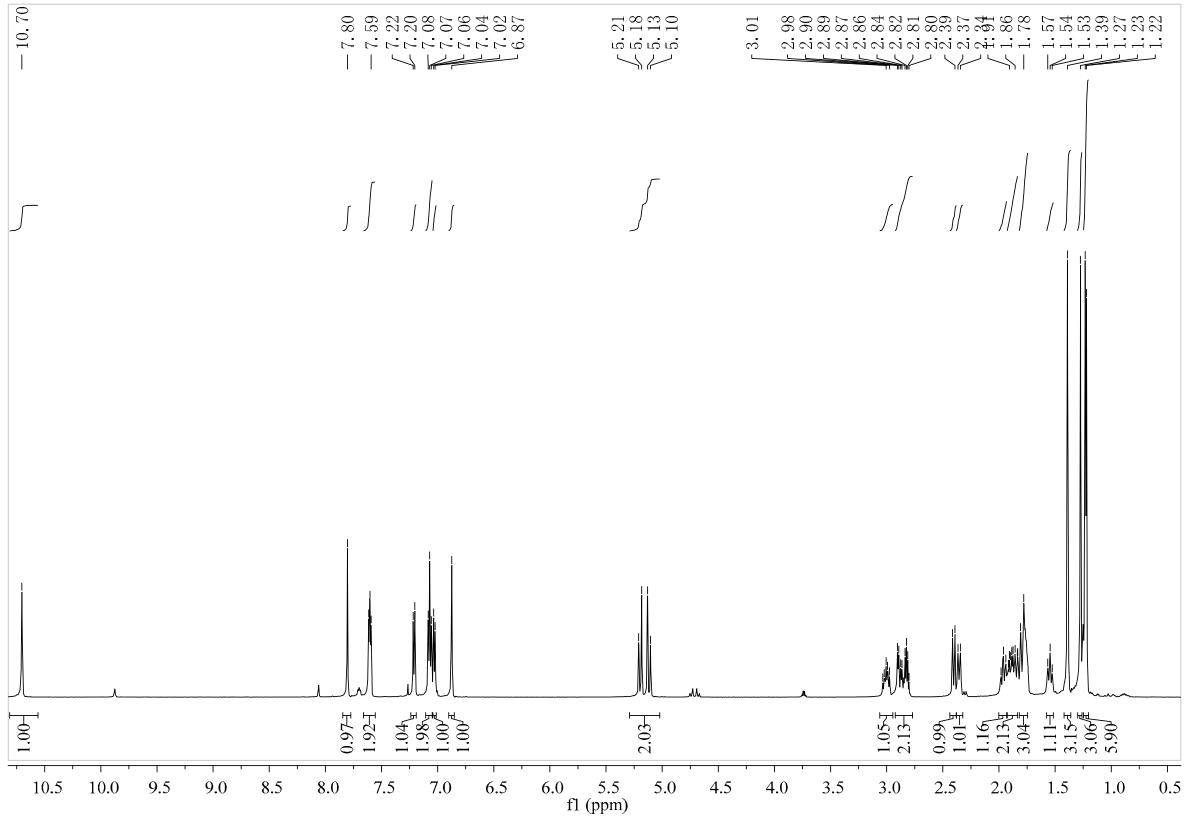

Figure S81. ^{1}H-NMR spectrum of the target compound (**4s**) in $CDCl_3$

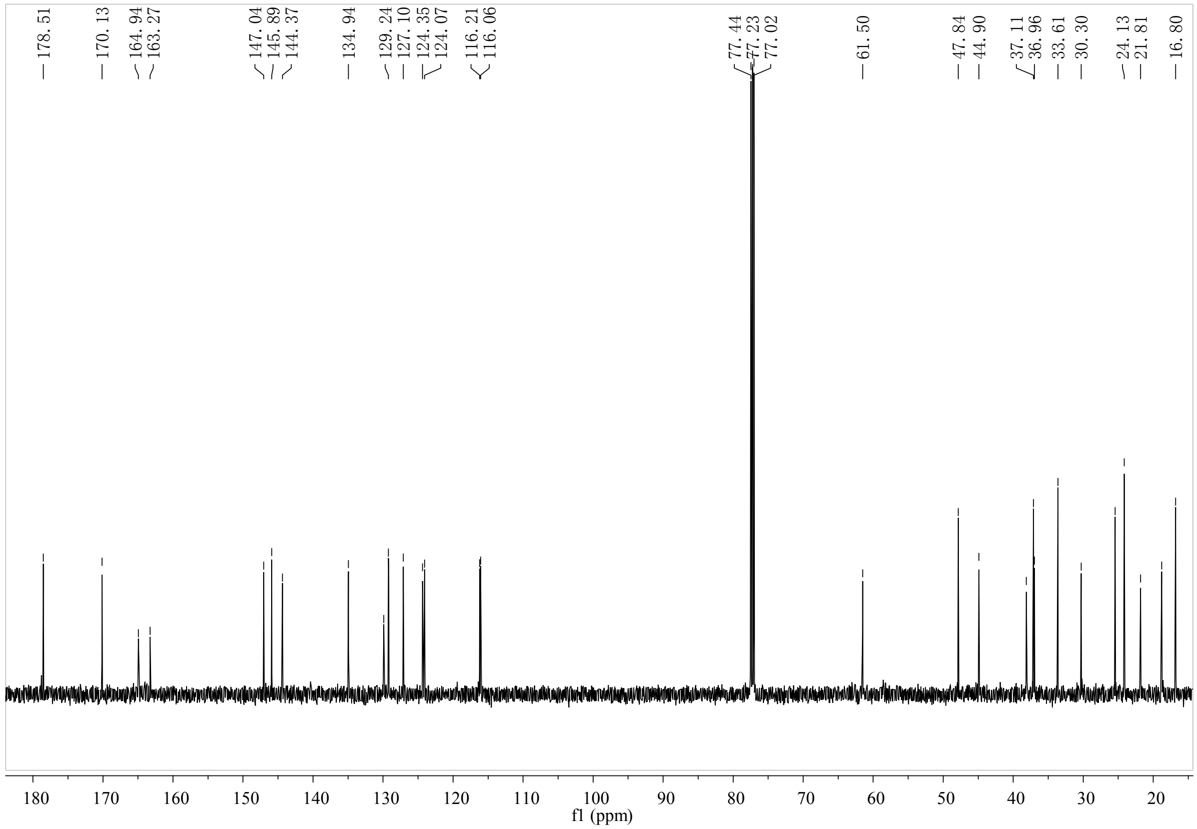

Figure S82. ^{13}C-NMR spectrum of the target compound (**4s**) in $CDCl_3$

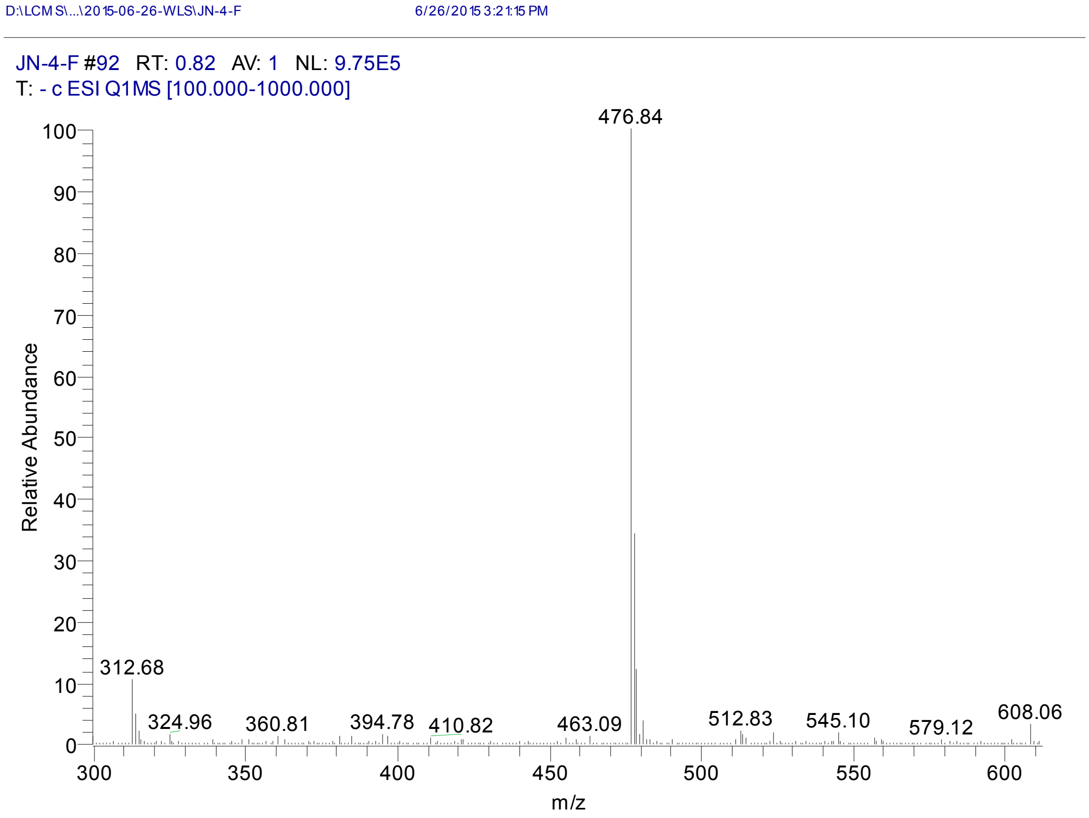

Figure S83. ESI-MS spectrum of the target compound (**4s**)

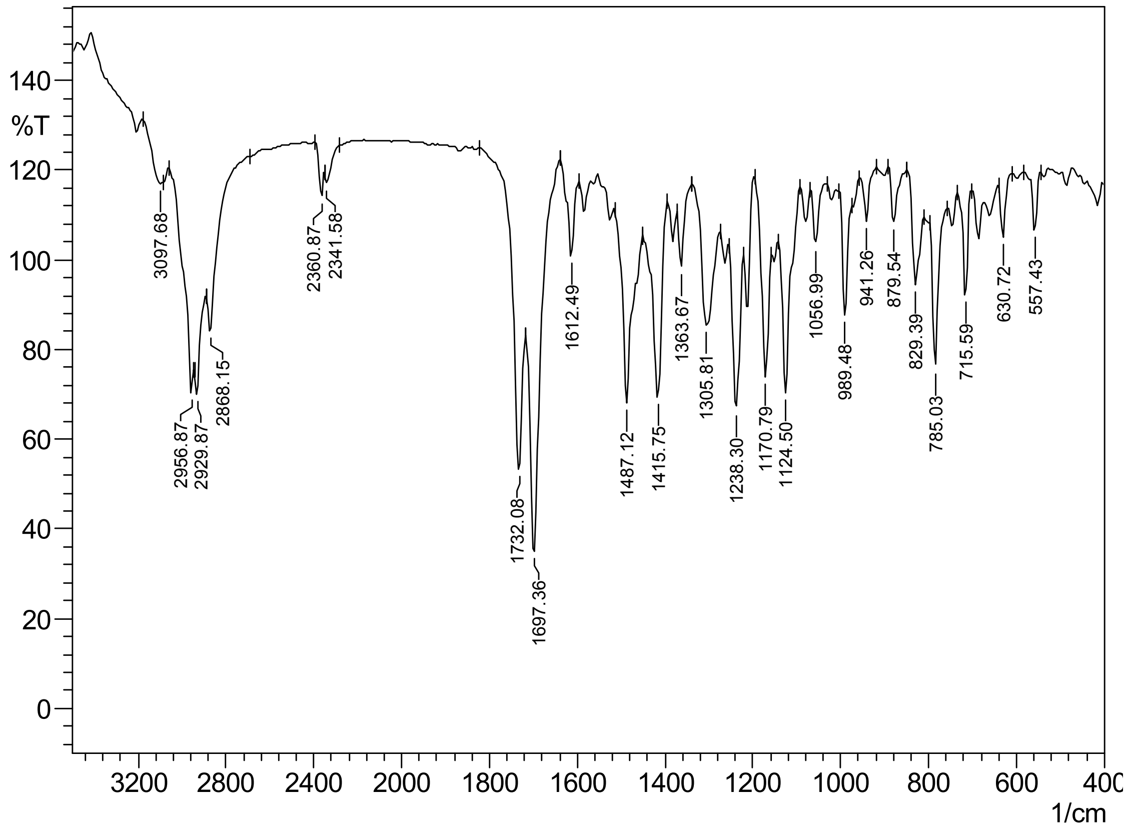

Figure S84. FTIR spectrum of the target compound (**4t**)

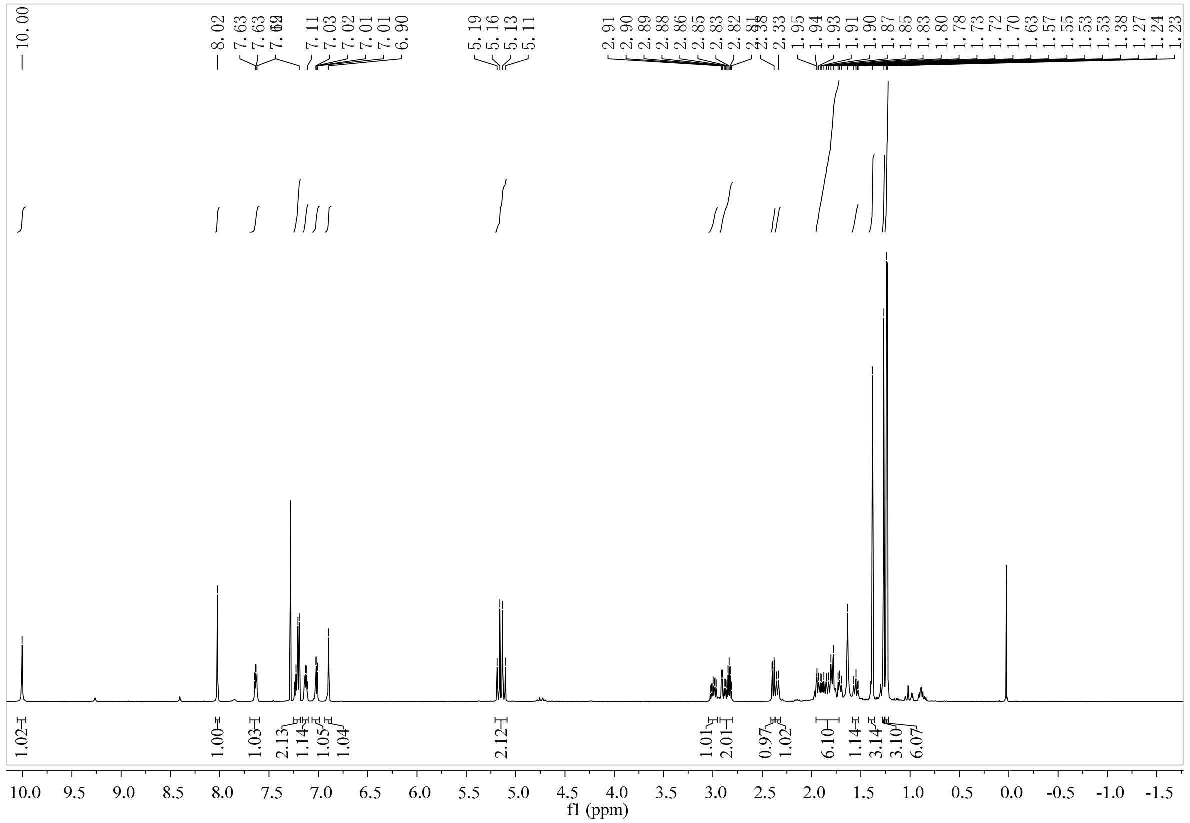

Figure S85. ^{1}H-NMR spectrum of the target compound (**4t**) in $CDCl_3$

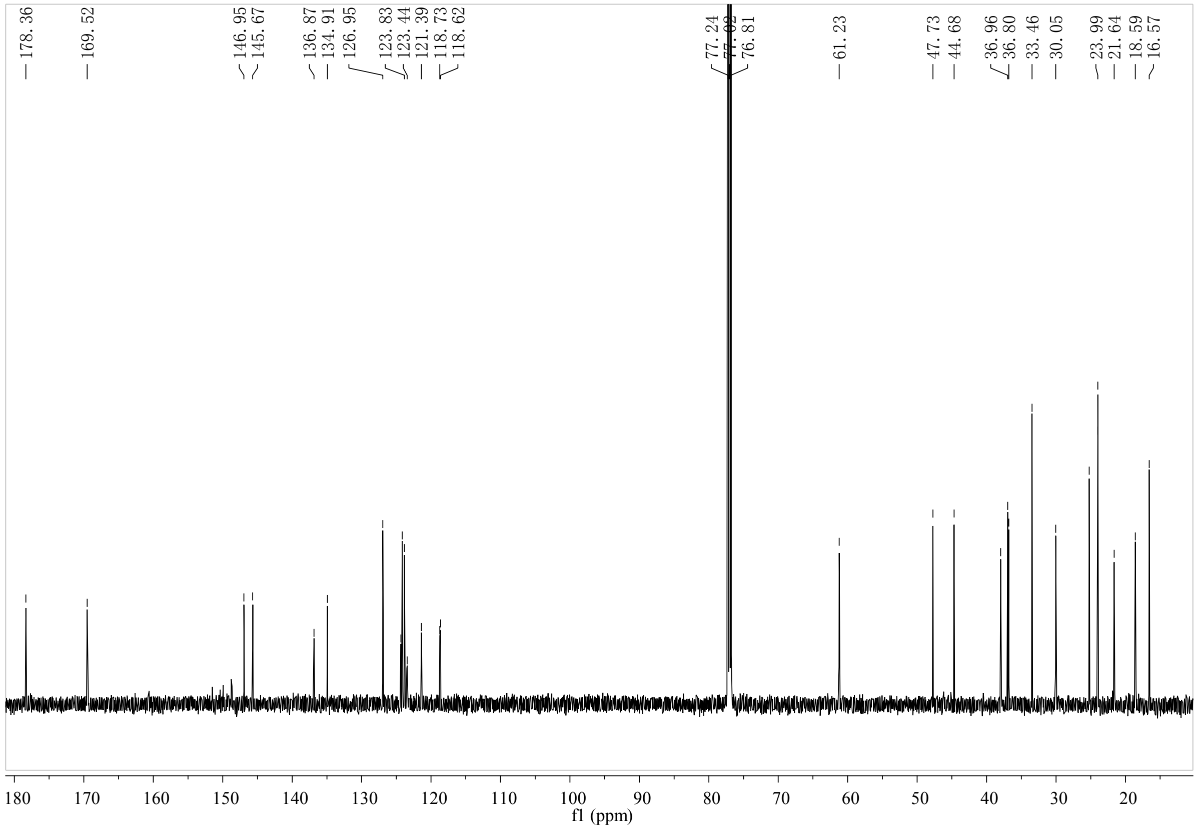

Figure S86. ^{13}C-NMR spectrum of the target compound (**4t**) in $CDCl_3$

<Spectrum>

Line#:1 R.Time:0.417(Scan#:26)
MassPeaks:14
RawMode:Single 0.417(26) BasePeak:495.1(543574)
BG Mode:None Segment 1 - Event 1

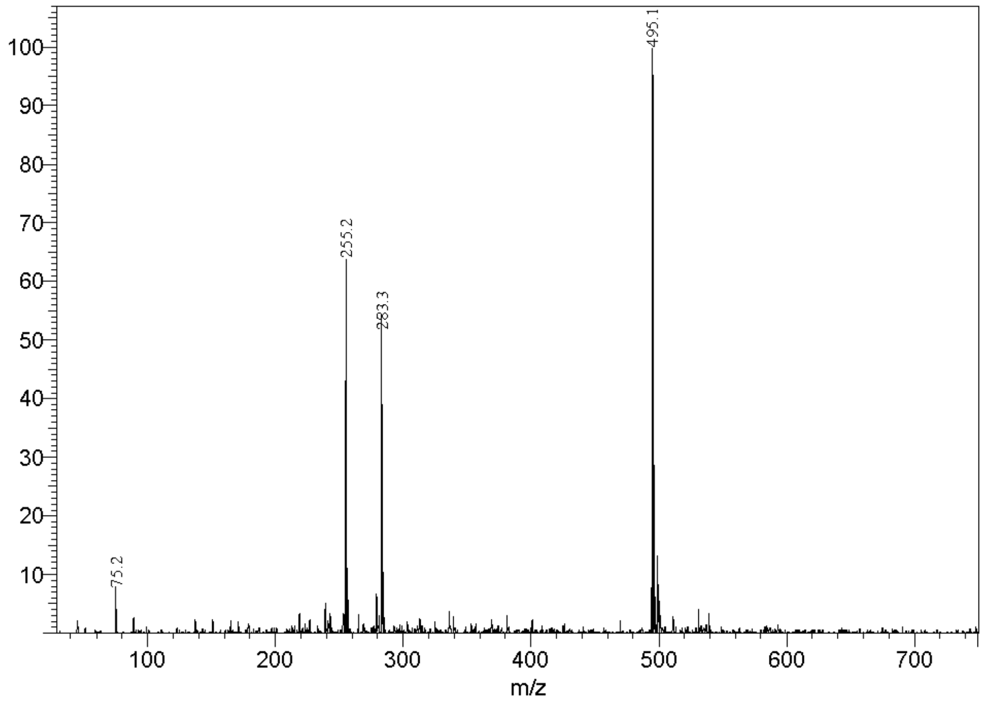

Figure S87. ESI-MS spectrum of the target compound (**4t**)

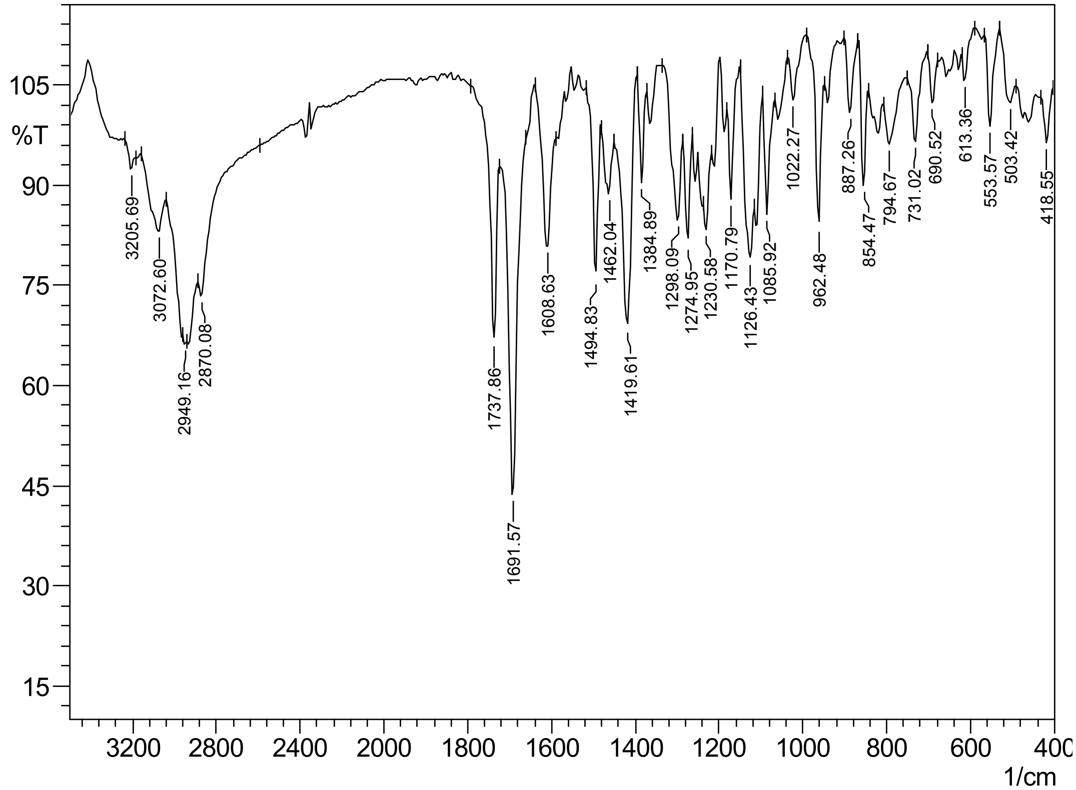

Figure S88. FTIR spectrum of the target compound (**4u**)

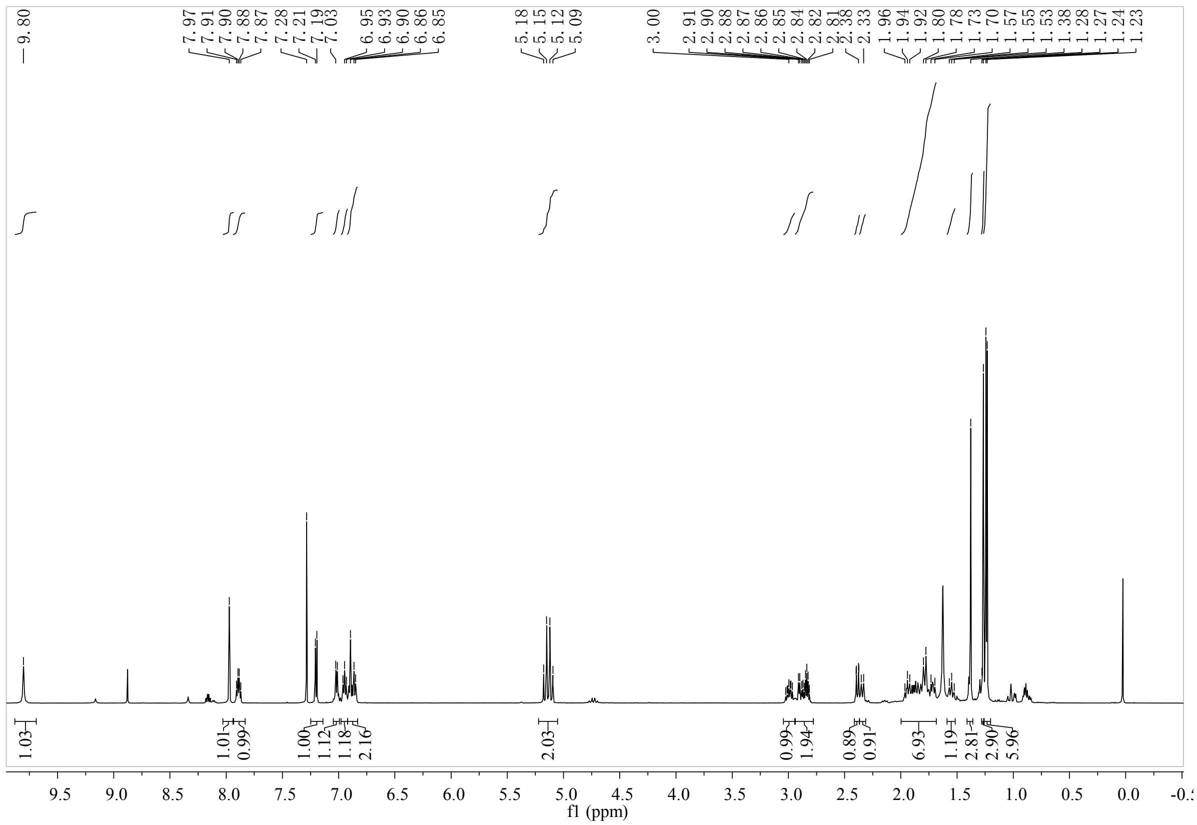

Figure S89. ^{1}H-NMR spectrum of the target compound (**4u**) in $CDCl_3$

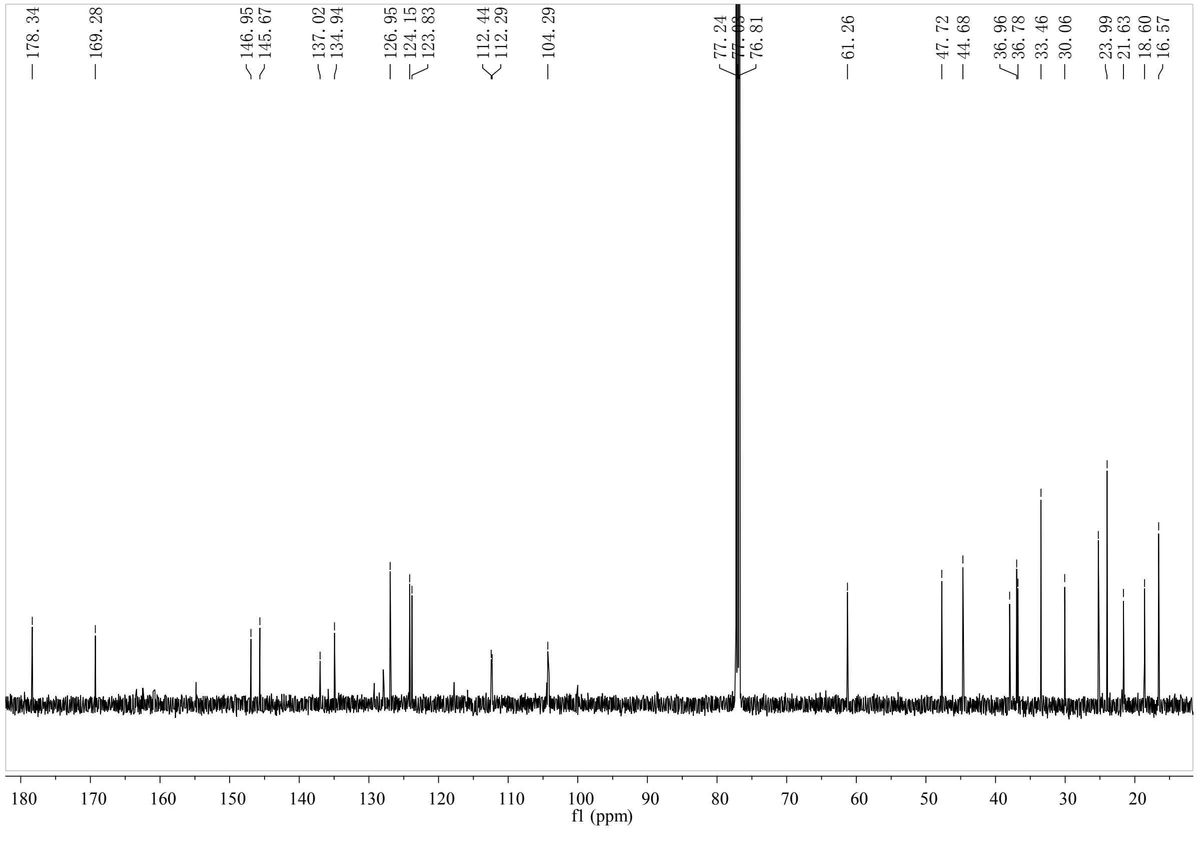

Figure S90. ^{13}C-NMR spectrum of the target compound (**4u**) in $CDCl_3$

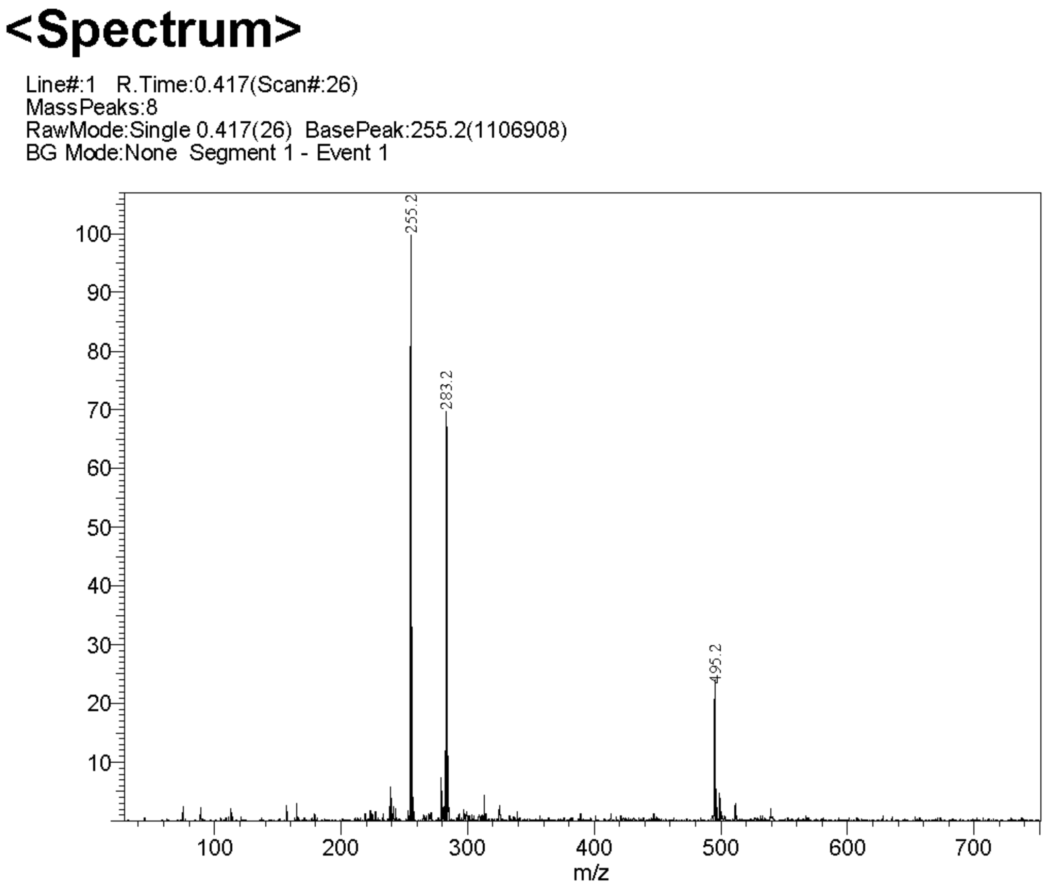

Figure S91. ESI-MS spectrum of the target compound (**4u**)

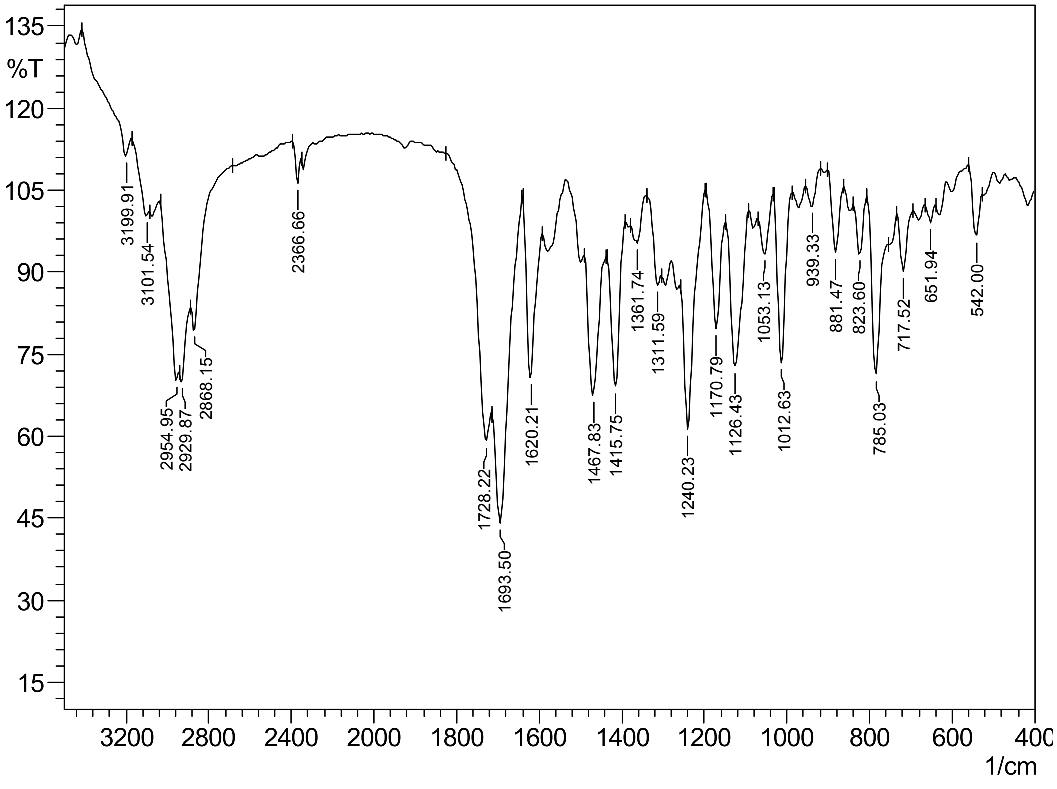

Figure S92. FTIR spectrum of the target compound (**4v**)

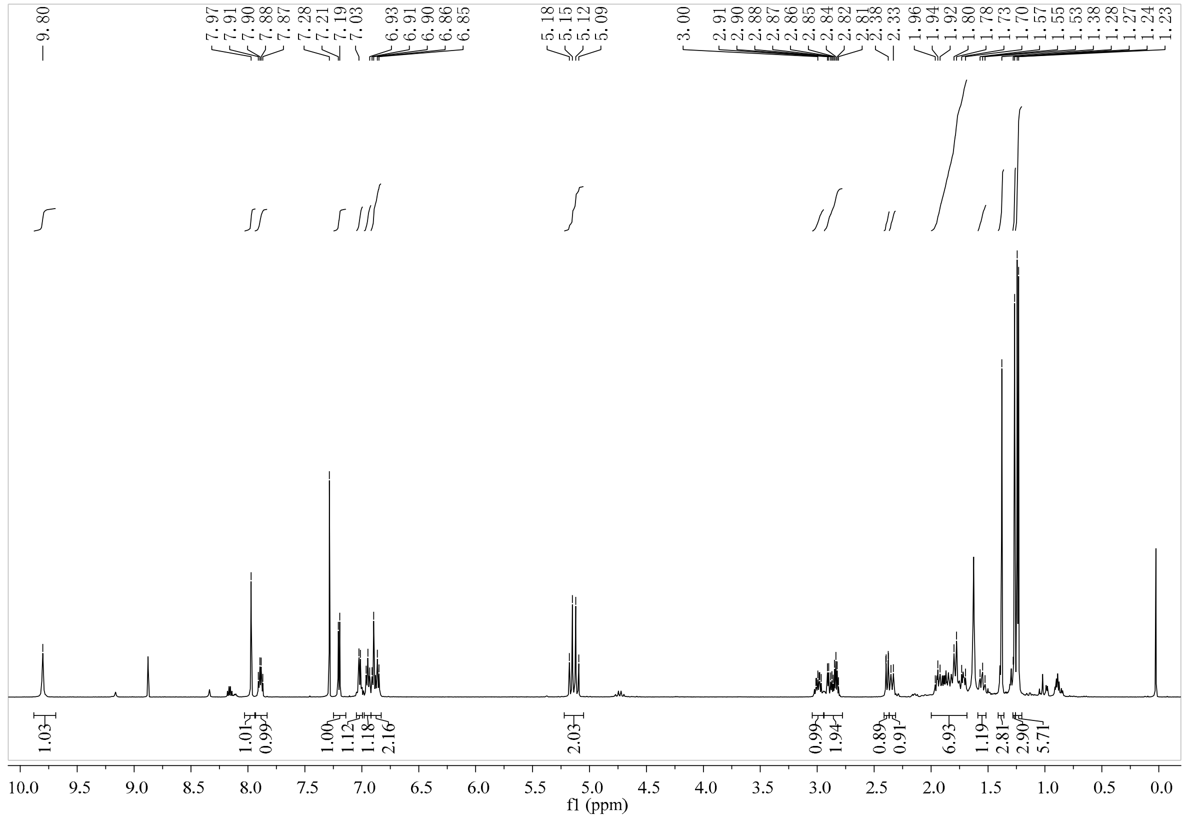

Figure S93. ^{1}H-NMR spectrum of the target compound (**4v**) in $CDCl_3$

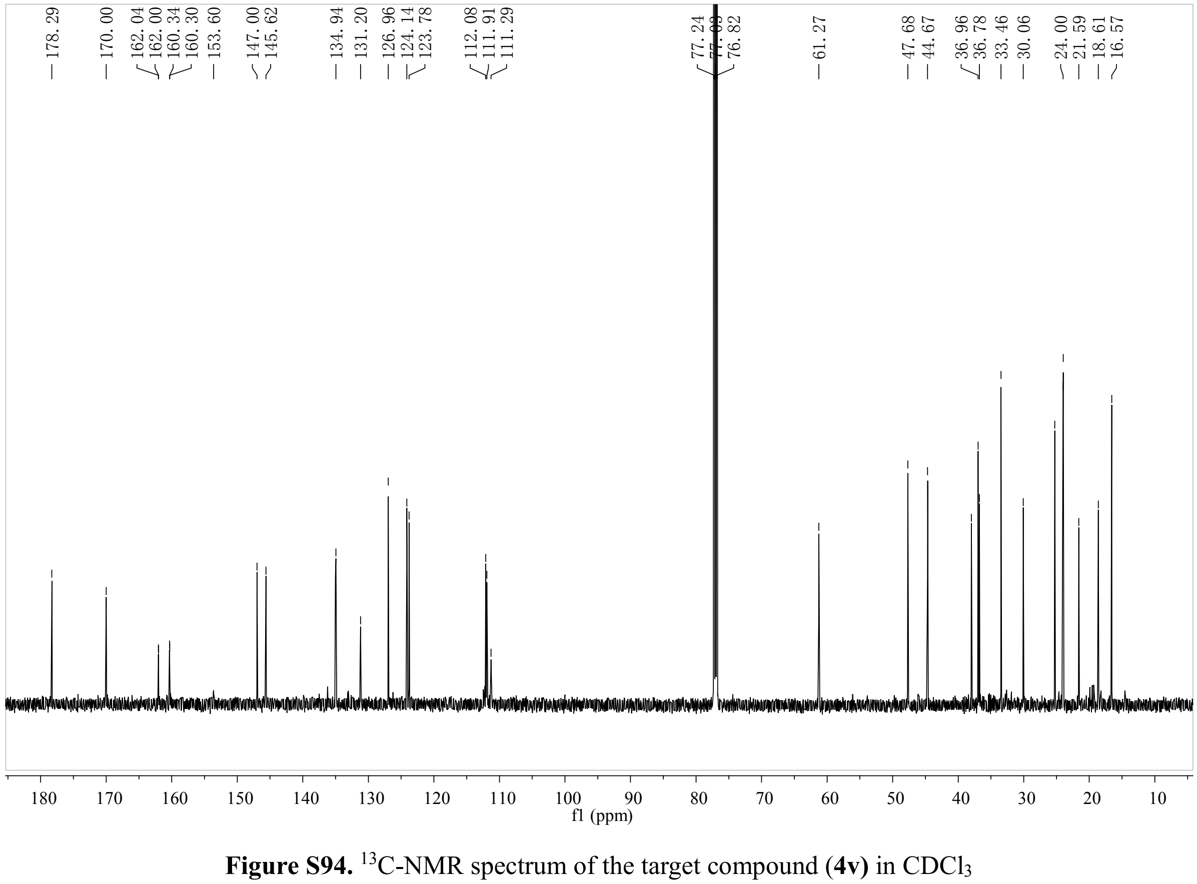

Figure S94. ^{13}C-NMR spectrum of the target compound (**4v**) in $CDCl_3$

<Spectrum>

Line#:1 R.Time:0.417(Scan#:26)
MassPeaks:13
RawMode:Single 0.417(26) BasePeak:495.1(417737)
BG Mode:None Segment 1 - Event 1

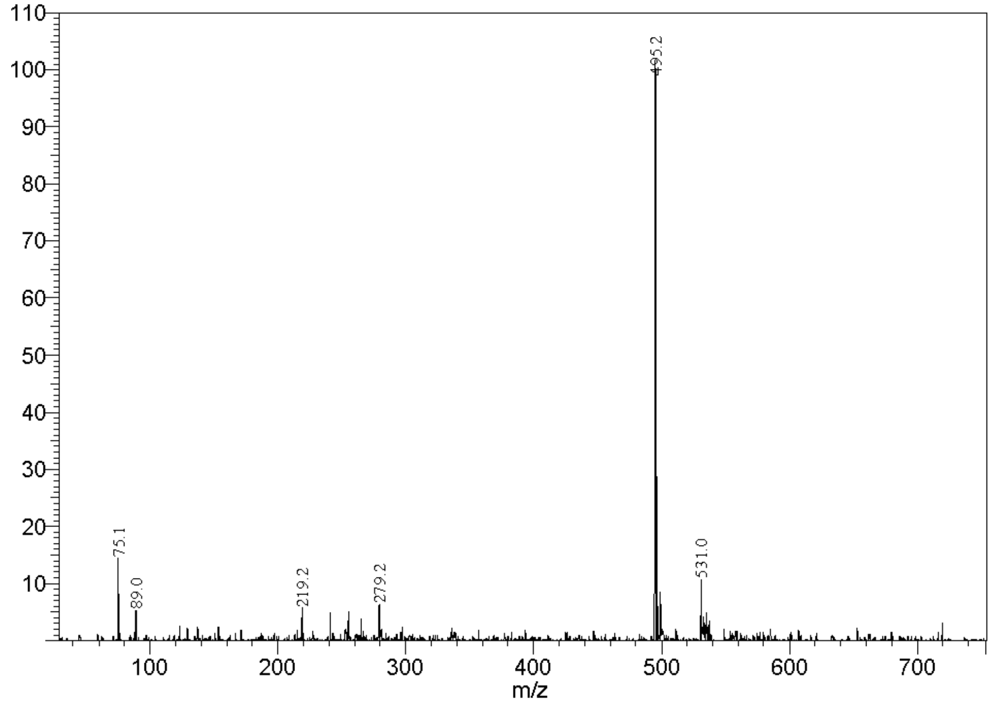

Figure S95. ESI-MS spectrum of the target compound (**4v**)

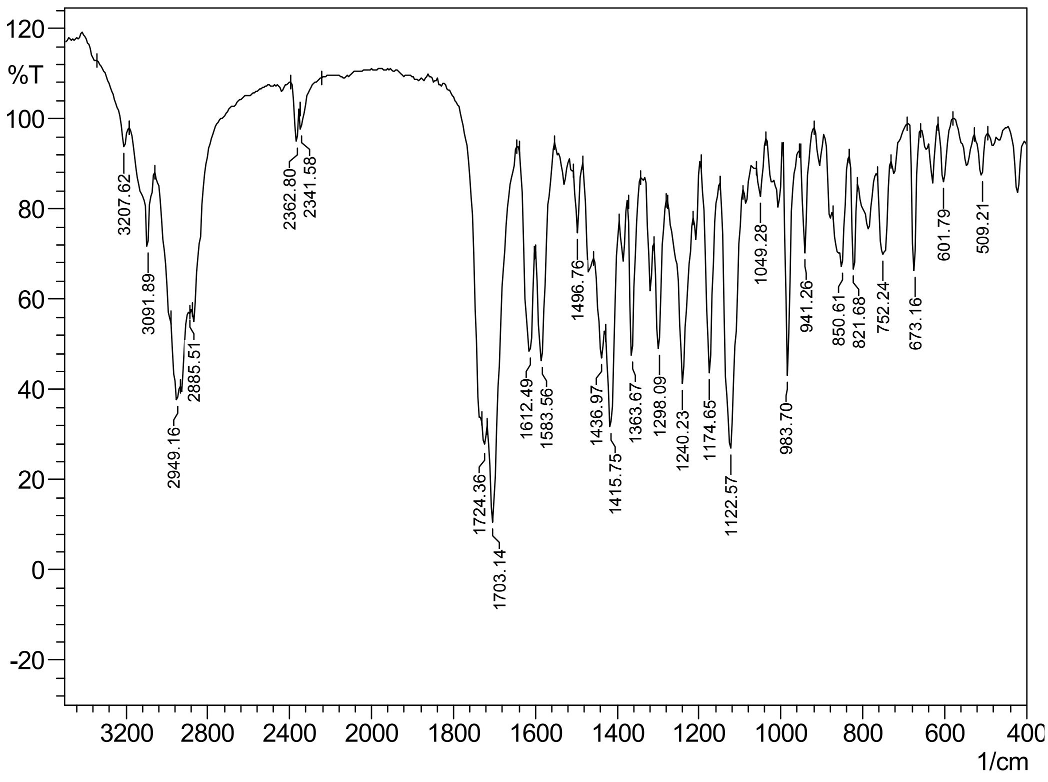

Figure S96. FTIR spectrum of the target compound (**4w**)

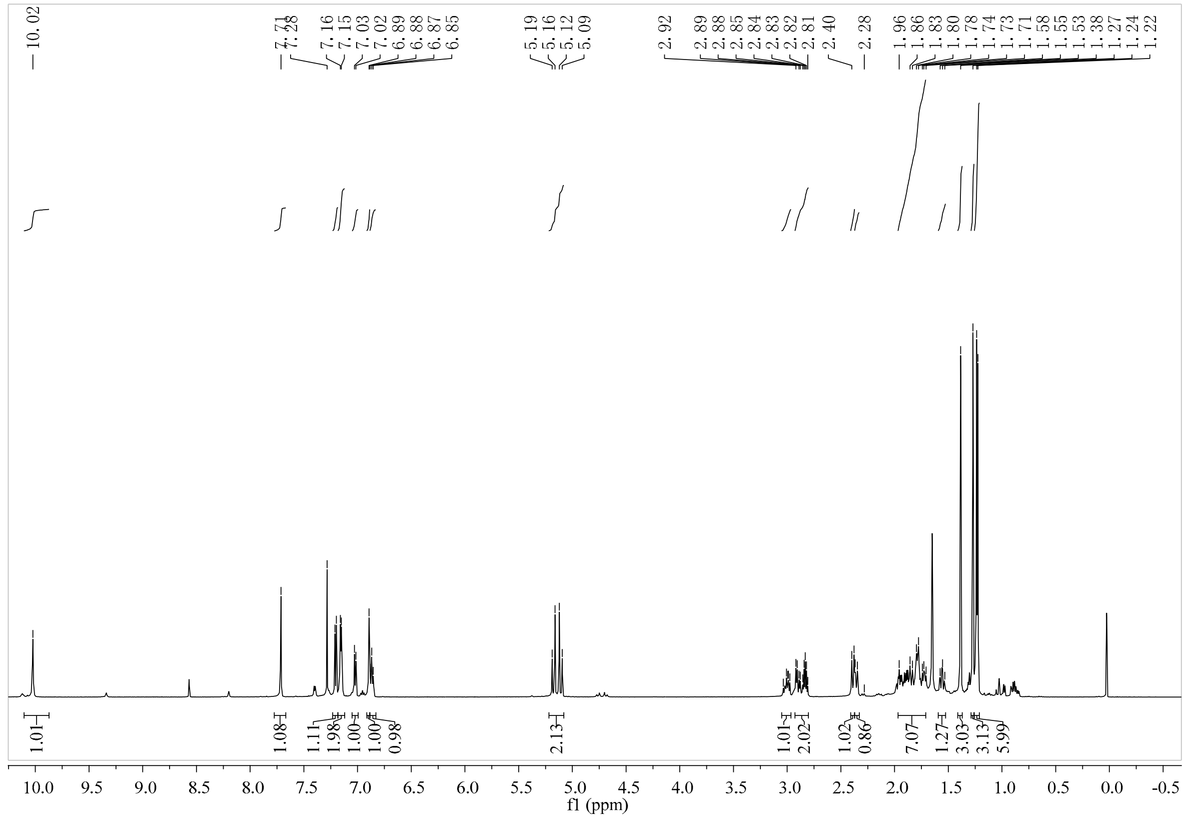

Figure S97. ^{1}H-NMR spectrum of the target compound (**4w**) in $CDCl_3$

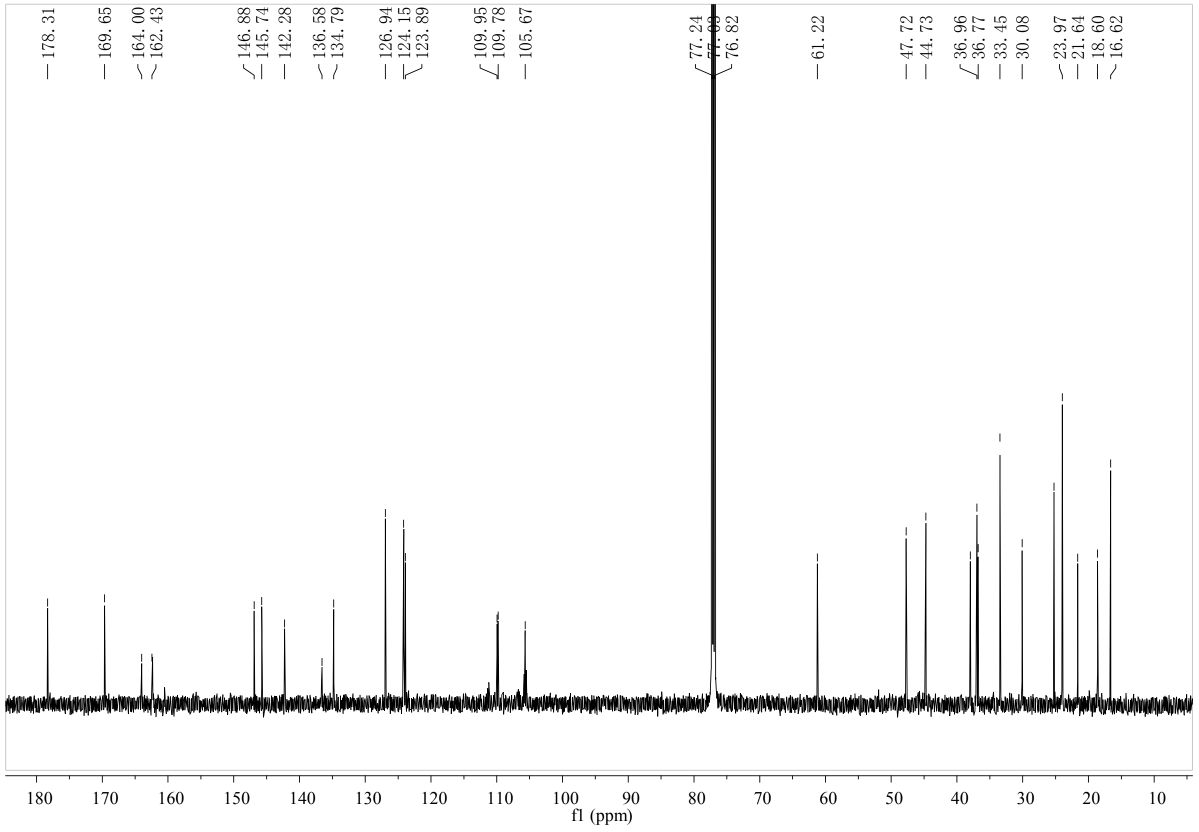

Figure S98. ^{13}C-NMR spectrum of the target compound (**4w**) in $CDCl_3$

<Spectrum>

Line#:1 R.Time:0.450(Scan#:28)
MassPeaks:13
RawMode:Single 0.450(28) BasePeak:495.1(354149)
BG Mode:None Segment 1 - Event 1

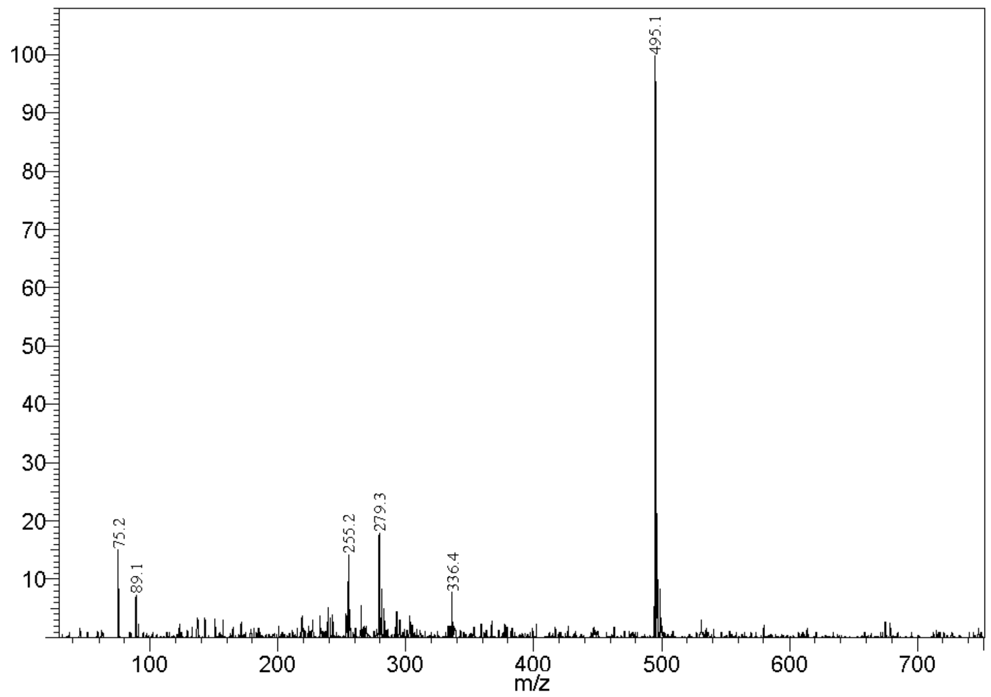

Figure S99. ESI-MS spectrum of the target compound (**4w**)

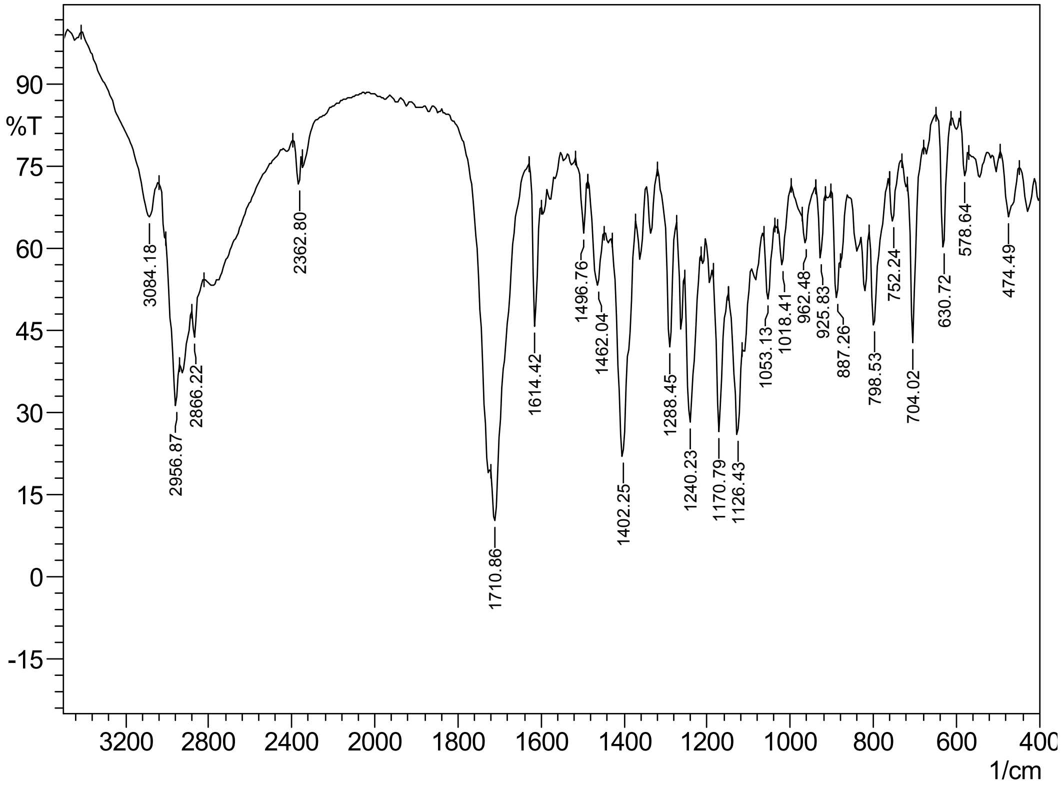

Figure S100. FTIR spectrum of the target compound (**4x**)

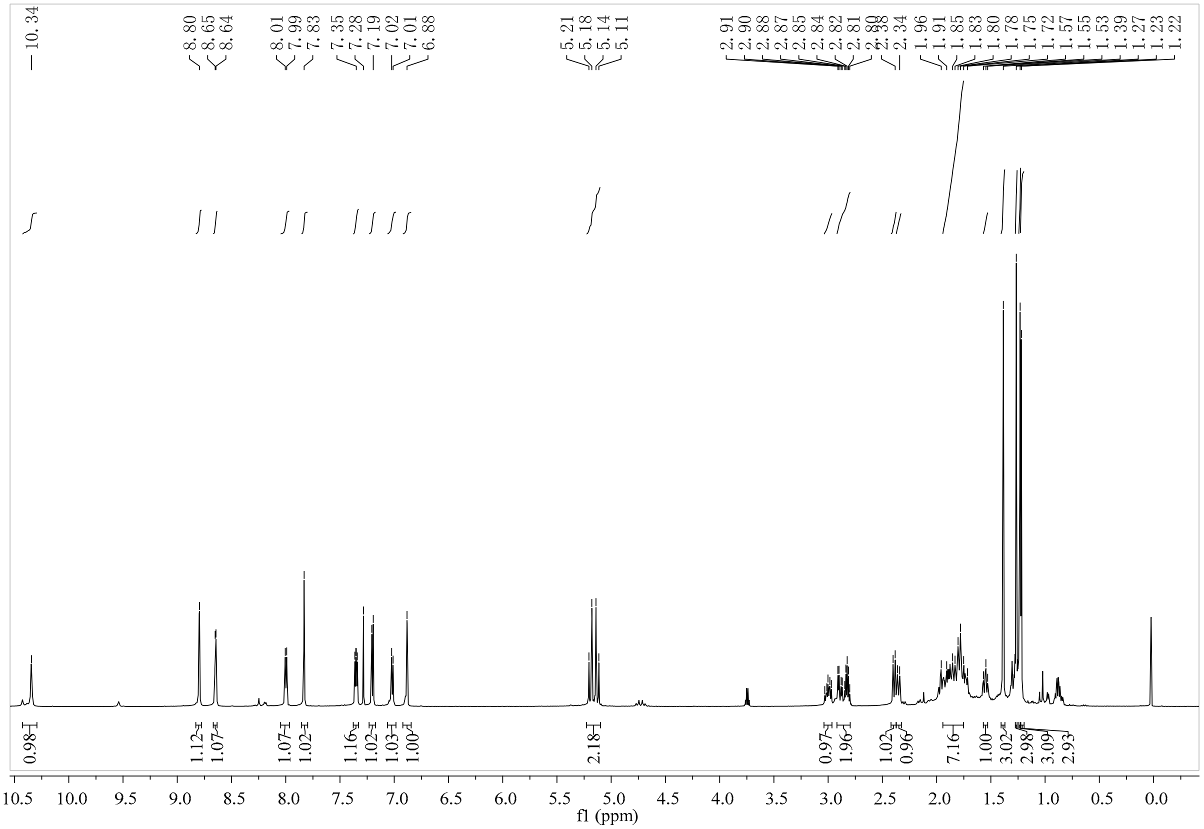

Figure S101. ^{1}H-NMR spectrum of the target compound (**4x**) in $CDCl_3$

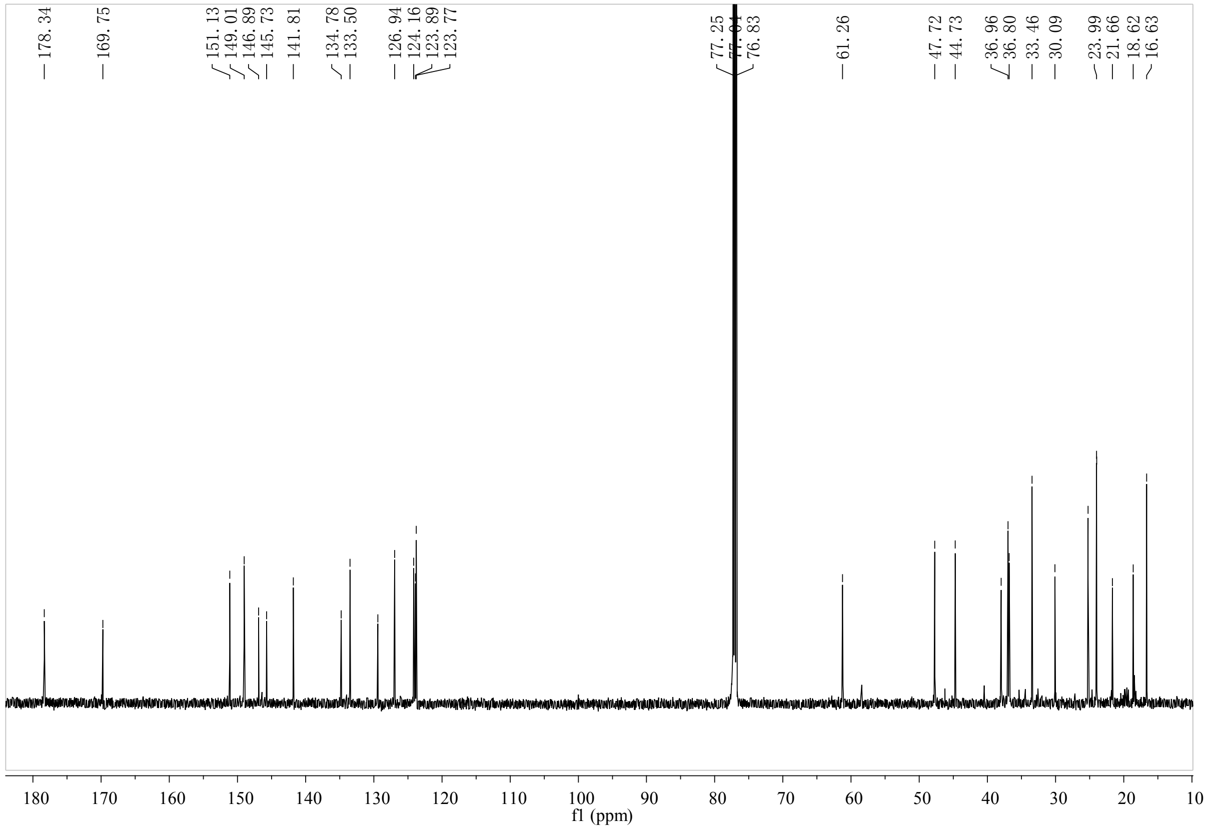

Figure S102. ^{13}C-NMR spectrum of the target compound (**4x**) in $CDCl_3$

<Spectrum>

Line#:1 R.Time:0.417(Scan#:26)
MassPeaks:15
RawMode:Single 0.417(26) BasePeak:460.2(332240)
BG Mode:None Segment 1 - Event 1

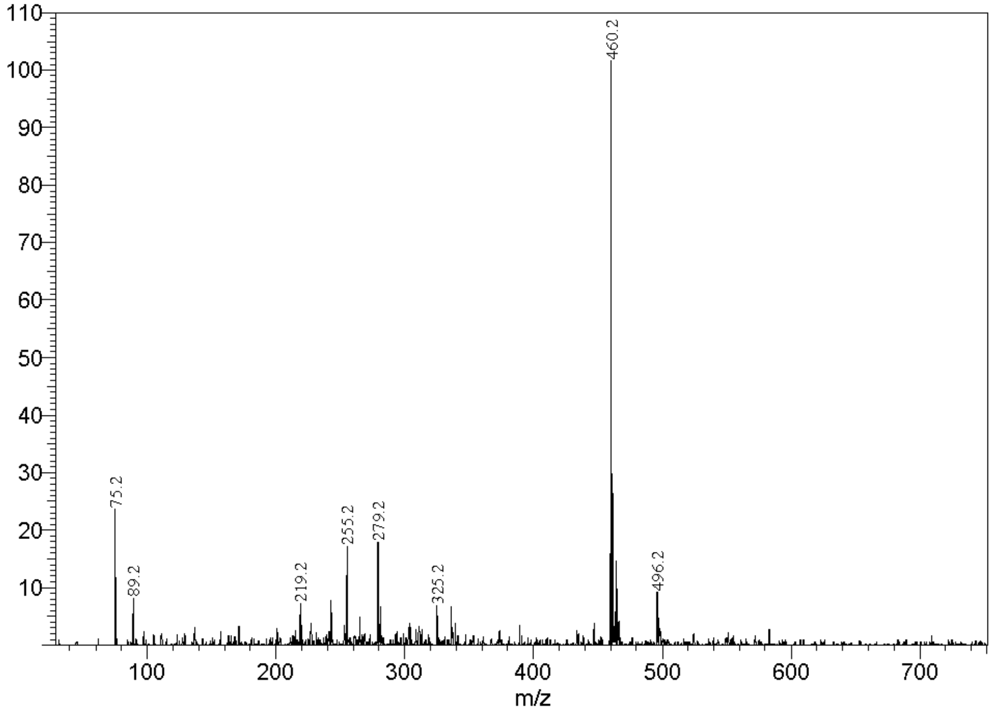

Figure S103. ESI-MS spectrum of the target compound (**4x**)
